# Optimizing Genomic Control in Hit Network-Target Set Model Associations with Lung Adenocarcinoma

**DOI:** 10.7150/jca.78138

**Published:** 2023-01-01

**Authors:** Bo Wang, Qiong Liu, Yanda Li, Lin Chen, Shuang Guan, Rong Li, Bing Li, Yanan Yu, Jun Liu, Yingying Zhang, Zhong Wang

**Affiliations:** 1 Institute of Basic Research in Clinical Medicine, China Academy of Chinese Medical Sciences, Dongzhimen, Beijing 100700, China.; 2 Postdoctoral Research Station, China Academy of Chinese Medical Sciences, Beijing 100700, China.; 3 The Open University of China 75 Fuxing Rd, Haidian District, Beijing 100039.; 4 The Second Affiliated Hospital of Zhejiang Chinese Medical University (ZhejiangXinhuaHospital), Zhejiang Chinese Medical University, Hangzhou 310053, China.; 5 Rongcheng Hospital of Traditional Chinese Medicine, Weihai 264399, China.; 6 Institute of Chinese Materia Medica, China Academy of Chinese Medical Sciences, Dongzhimen, Beijing 100700, China.; 7 Dongzhimen Hospital, Beijing University of Chinese Medicine, Haiyuncang, Beijing 100700, China.

**Keywords:** Network control, Hit network-target sets, Driver nodes, Core module, Core nodes

## Abstract

**Background:** Hit network-target sets (HNSs), compiled sets of different network nodes of the same type, are available and play a significant role in cancer development but are notoriously more difficult to select than a single target. This is due to a combination of challenges attributed to the differential of node interactions, node heterogeneity, and the limitations of node-hit information.

**Methods:** In this study, we constructed a lung adenocarcinoma regulatory network using TCGA data and obtained different HNSs of driver nodes (DNs), core modules (CMs) and core nodes (CNs) through three kinds of methods. Then, the optimized HNS (OHNS) was obtained by integrating CMs, CNs and DNs, and the performance of different HNSs was evaluated according to network structure importance, control capability, and clinical value.

**Results:** We found that the OHNS has two main advantages, the central location of the network and the ability to control the network, and it plays an important role in the disease network through its multifaceted capabilities. Three unique pathways were discovered in the OHNS, which is consistent with previous experiments. Additionally, 13 genes were predicted to play roles in risk prognosis, disease drivers, and cell perturbation effects of lung adenocarcinoma, of which 12 may be candidates for new drugs and biomarkers of lung adenocarcinoma.

**Conclusion:** This study can help us understand and control a network more effectively to determine the development trend of a disease, design effective multitarget drugs, and guide the therapeutic community to optimize appropriate strategies according to different research aims in cancer treatment.

## Introduction

Diseases place the body in an unbalanced state. The purpose of treating diseases is to correct this imbalance and adjust it to a healthy status. Effectively controlling the disease network and modifying the states of biological systems to desired states by manipulating signals is a research hotspot. Excellent control methods can provide key therapeutic targets for diseases, assist in the design of optimal compounds with the expected effects, and discover new indications for old drugs 1. Hit network-target sets (HNSs), sets of different network nodes compiled from the same available types, play a significant controlling role in disease development. Here, we define the “hit network target set” as a combination of multicomponent units that occupy the core of the network structure and have control over the network, which can provide important driving forces for system perturbation of the network. However, knowledge about HNSs is far from comprehensive, which hampers disease control and the discovery of new drugs. Recently, the continuous accumulation of omics and big data has provided new methods for network-based target research and prediction. The success of network-based drug discovery depends on the choice of drug targets. Therefore, determining the HNS in a network has become a key issue.

It is challenging to capture the most relevant features of HNSs. Researchers have conducted different exploratory studies regarding the importance of HNSs. For example, the structural properties of networks are mainly based on node centrality 2, 3, network core module nodes (CMs) for measuring the importance of node sets 4 and driver nodes (DNs) based on structural control theory 5, where the aim is to identify the key nodes in complex networks as drug targets. Module nodes allow the automated prediction of protein complexes from qualitative protein-protein interaction data and are thus able to help predict the function of unknown proteins. Core nodes (CNs), also called hub nodes, are used for ranking elements in a network by their network features to infer their importance in the network and can help us identify central elements of biological networks 6. DNs control targets in complex networks obtained by a full-fledged, abstract state-dependent dynamical model of diffusion dynamics in genomic networks and are often used as a way to study cancer 7.

Although great progress has been made regarding computational methods for the identification of HNSs based on network topologies, there are still several challenges that researchers have to address. For example, highly connected genes (hubs), because of their strong centrality, have a significant influence on the structure of the network, which is important for cell growth and survival 8. Taking such nodes as targets can indeed produce a large disturbance in the network, but the deletion of such genes will result in lethality or infertility since the organism cannot survive without them. It is necessary to find a method that can not only avoid hubs but also achieve a certain control over the network. Hu et al. 9 introduced the concept of driver nodes, defined as especially critical nodes that have a strong ability to influence the states of other nodes and a weakened susceptibility to the states of the other nodes. Moreover, injecting control inputs (drugs, signals from the environment or within the organism, etc.) to critical and high-frequency driver nodes can regulate the whole state of the disease, which indicates that critical and high-frequency driver nodes are potential drug targets 10.

Inspired by these findings, to improve the accuracy and effectiveness of disease control, a method needs to be chosen to achieve a balance that can not only attack the disease but also maintain the survival of the organism. In this study, we obtained the network HNS based on the multiangle characteristics of network topology and structure control theory. This method was evaluated from the perspective of network information transmission and structural integrity. It could improve the control of complex dynamical systems in general and biochemical regulation in particular. We expect that this will help to select appropriate drug targets and provide value for rational drug design.

## Materials and Methods

### Data source

The Cancer Genome Atlas (TCGA) database (http://cancergenome.nih.gov) contains over 20000 primary cancer and matched normal samples spanning 33 cancer types, providing an important resource for evaluating the biological relevance of cancer genomics discovery. This study aims to integrate sufficient data, including as much relevant important information as possible, so all the mRNA-Seq data were generated using lung adenocarcinoma (LUAD) tissues and normal tissues from 594 samples by TCGA (LUAD=535, normal=59).

### Calculation of differentially expressed genes

The limma package and biocLite edgeR from R software v3.5.3 (R Foundation for Statistical Computing, Vienna, Austria) were used to study the differential expression of mRNAs. The adjusted P value was analyzed to correct for false-positive results in TCGA. Adjusted P < 0.05 and fold change=1.5 were defined as the thresholds for screening differential expression of mRNAs 11.

### Construction of the gene regulatory network

We constructed a disease regulatory network by mapping the differentially expressed gene set to the human gene regulatory network. The human gene regulatory network was constructed by integrating annotations from three high-quality pathway databases: Reactome, KEGG, and the NCI-Nature Pathway Interaction Database, which were compiled from Hu et al. 9.

### Identification of driver nodes

The identification of driver nodes can be formulated as a maximum-cardinality bipartite matching problem in a bipartite graph corresponding to the original network. The details of the driver nodes and the identification algorithm can be found in 5. In this work, the Hopcroft-Karp algorithm 12 was utilized to solve the maximum-cardinality matching problem. Control centrality was developed to quantify the ability of a node to control a network, which equals the dimension of the controllable subspace, and the algorithm used to calculate the control centrality was proposed in 13.

### Core gene identification

Maximal clique centrality (MCC) 14 captures more essential proteins in the top-ranked list in both high-degree and low-degree genes. Given a node v, the MCC of v is defined as MCC(v) = ∑C∈S(v)(|C| - 1)!, where S(v) is the collection of maximal cliques that contain v and (|C|-1)! is the product of all positive integers less than |C|. If there is no edge between the neighbors of node v, then MCC(v) is equal to its degree 6.

### Modular screening and stability

Three module-screening methods, connected components, the Markov cluster algorithm (MCL; parameters: number of iterations=16) 15, and molecular complex detection (MCODE; parameters: degree cutoff=2, K-core=2, and node score threshold=0.2) 4, were compared. The connected components are simple divisions based on connectivity 16. The MCL algorithm assigns genes into families based on precomputed sequence similarity information. MCODE is based on vertex weighting by local neighborhood density and outward traversal from locally dense seed genes to isolate dense regions according to the given parameters 4. The network structure entropies of these regions are calculated to balance the selective speculation 17. The network structure entropy is defined as follows:


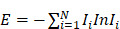

(1)

where *N* is the number of nodes in the network and *Ii* is the importance of node *i.* A smaller entropy value means a higher similarity between modular nodes, thereby determining the module stability.

### Core module identification using the multiple-modular-characteristic fusing approach

Core module genes were regarded as important, and the edges between these modules were derived from the intermolecular relations across modules. The approaches of multiple modular characteristic fusing (MMCF) 4, including degree, maximum neighborhood component (MNC), density of maximum neighborhood component (DMNC), maximal clique centrality (MCC), closeness (Clo), eccentricity (EC), radiality (Rad), bottleneck (BN), stress (Str), betweenness centrality (BC), and edge percolated component (EPC), were used for this search process 18-23. After ranking the results of these 11 methods, the rank-sum ratio was used for comprehensive quantitative evaluation.

### Performance assessment of the HNS

We removed each CN, CM and DN from the network and then observed the change in the characteristic path length and giant component. To better compare the change trends of the characteristic path length and giant component, we randomly selected genes from the CNs, DNs, CMs, RNs (random nodes in the whole network) and OHNS (the combination of the CMs and the top-50 control centrality nodes among the DNs). The cardinal number started at 5, and 5 genes were randomly added each time for a total of 10 times. For each gene removed, we recorded the size of the graph's remaining giant component and characteristic path length.

#### (i) Characteristic path length (L)


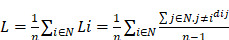

(2)

where i and j are the different nodes in the network, Li is the average distance between node *i* and all other nodes, and *dij* is the distance between nodes *i* and *j* 24.

#### (ii) Giant component (GC)

The giant component is the largest connected component in a network, and the fraction was calculated by dividing the number of nodes in the giant component by the total number of nodes in the network 7.

#### (iii) Calculation of the F-measure

To provide comprehensive comparisons of HNSs, the key cancer genes annotated in the list of drug targets and biomarker genes for lung cancer (Comparative Toxicogenomics Database, http://ctdbase.org/) were adopted to assess the F-measure, considering both the precision and the recall of the predicted HNS using the formula:


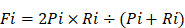

(3)

where *Pi* denotes the fraction of correctly predicted DNs, CNs or CMs among the predicted DNs, CNs or CMs and Ri denotes the fraction of correctly predicted DNs, CNs or CMs among all the key cancer genes.

#### (iv) Perturbation effects

We used genome-scale CRISPR‒Cas9 knockout data from the Cancer Dependency Map (https://depmap.org/portal/). The necessary genes for perturbation of 197 non-small cell lung cancer cell lines among the CMs, CNs and DNs were collected. A lower Chronos score indicates a higher likelihood that the gene of interest is essential in a given cell line. A score of 0 indicates that a gene is not essential; correspondingly, -1 is comparable to the median of all panessential genes.

### Functional enrichment analysis

Metascape (http://metascape.org) is a reliable, intuitive tool for gene annotation and gene list enrichment analysis 25. In our study, Metascape version 3.5 was used for functional enrichment analysis, with species restricted to *Homo sapiens*. Gene Ontology (GO) enrichment analysis and Kyoto Encyclopedia of Genes and Genomes (KEGG) pathway enrichment analysis were able to describe the biological features of HNSs. Cytoscape ClueGO 26 was used to find the genes linking different pathways. Modified Fisher's exact test and Benjamini's test were reutilized for calculating and correcting P values (P < 0.05). Biological processes (BPs), cellular components (CCs), and molecular functions (MFs) were included in the GO enrichment analysis.

### Statistics

Statistical analyses were performed using SPSS 23.0, and a P value of < 0.05 was considered statistically significant. The chi-square test was used to analyze the (in- and out-) degree distribution ratio of multiple groups in a network. The Pearson correlation coefficient was used to analyze the correlation between the (in- and out-) degree, characteristic path length and giant components of the HNSs.

### Risk prognostic assessment

RNA-sequencing expression (level 3) profiles and corresponding clinical information for LUAD were downloaded from the TCGA dataset (https://portal.gdc.cancer.gov/). For the Kaplan-Meier curves, p values and hazard ratios with 95% confidence intervals were generated by log-rank tests and univariate Cox proportional hazard regression. All the analysis methods and R packages were implemented in R (Foundation for Statistical Computing 2020) version 4.0.3. p < 0.05 was considered statistically significant.

### Data retrieval for replication and validation analyses

Validation and replication of the HNS was performed using datasets (GSE27262) in the Gene Expression Omnibus (GEO). Databases were downloaded in their raw states to maximize platform and annotation information, and then the data were normalized. The raw data were downloaded as MINiML files. The limma package in R software was used to study differentially expressed mRNAs. The adjusted P value was analyzed to correct the false positive results in the GEO datasets. “Adjusted P < 0.05 and Log (Fold Change) >1 or Log (Fold Change) < -1” were defined as the thresholds for the differential expression of mRNAs.

## Results

### Identification of the HNS

From TCGA, we obtained 8803 differentially expressed genes, including 7342 upregulated genes and 1461 downregulated genes. After removing single nodes, we obtained a regulatory network comprising 834 nodes (genes) and 6035 directed edges (regulatory links). By using the MCC algorithm, we obtained the core genes, and the top 100 are shown in Supplementary Table S1. We used the Hopcroft-Karp algorithm to obtain 396 driver nodes. Finally, 201 driver nodes were selected according to the control centrality (see Methods, Supplementary Table S2). The MCODE, MCL and connected components were used to divide the disease network modules, and modules 28, 39, and 15 were obtained. The entropy of the MCODE, MCL and connected components was 5.105, 5.986, and 6.018, respectively (Supplementary Table S3).

According to the minimum entropy criterion, compared with two other methods (connected components and MCL), MCODE demonstrated strikingly consistent stability in each group. With the MMCF comprehensive ranking, we finally obtained No. 2 as the core module, which contained 50 genes (Supplementary Table S1) and 369 edges.

### The network distribution of HNSs shows the characteristics of clustering and scattering

**Figure 1:**
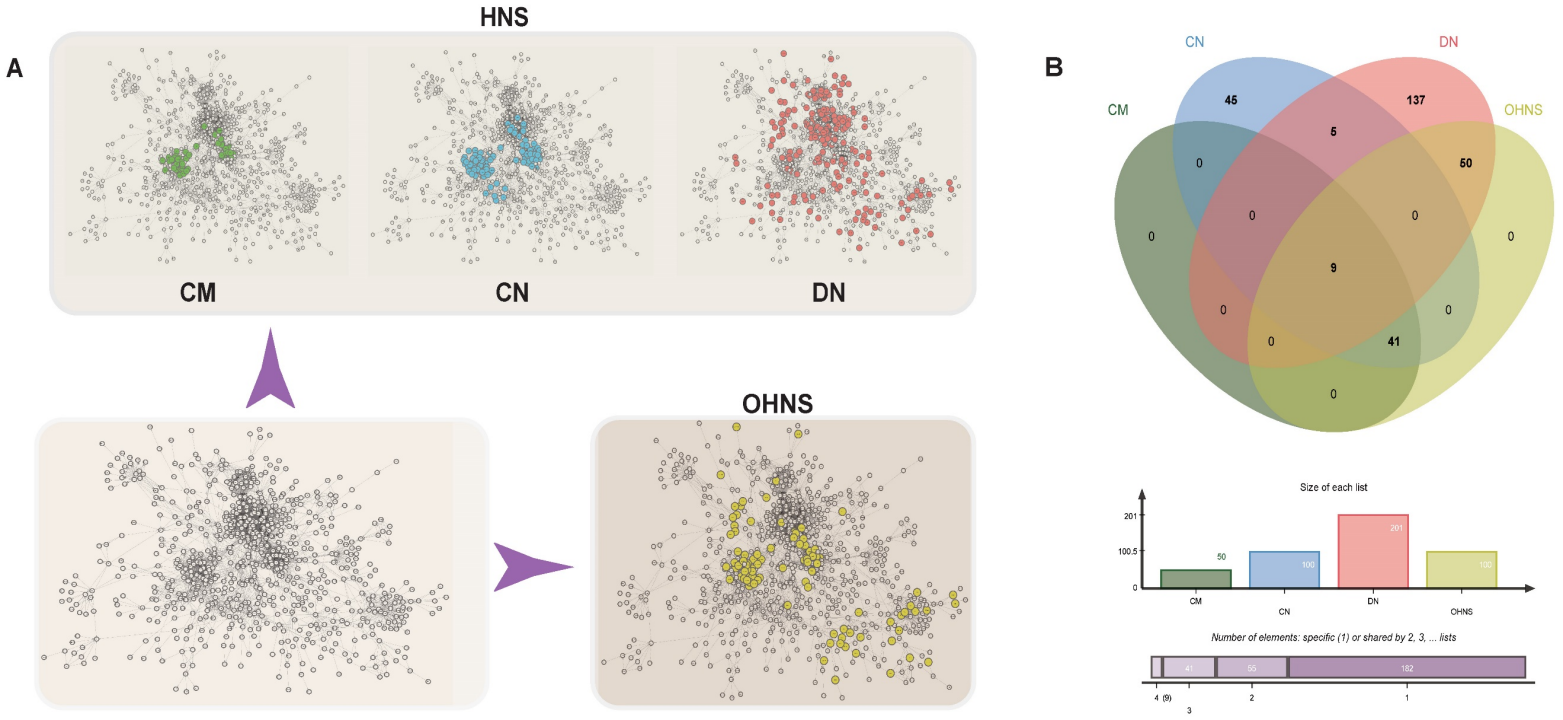
** Distribution map of the HNS. A.** Green represents CMs, blue represents CNs, pink represents DNs, and yellow represents the OHNS. **B.** The Venn diagram represents the relationships in the HNS. Charts showing the list size and the intersection size repartition are located below the diagram.

After removing the single nodes, we obtained a regulatory network (Fig. 1A). In this network, the distributions of CMs, CNs and DNs were very different. The CMs and CNs had more overlap (Fig. 1B), and they were more centralized in the network, while the DNs were more scattered.

### Driver nodes show out-degree-dominant characteristics

**Figure 2:**
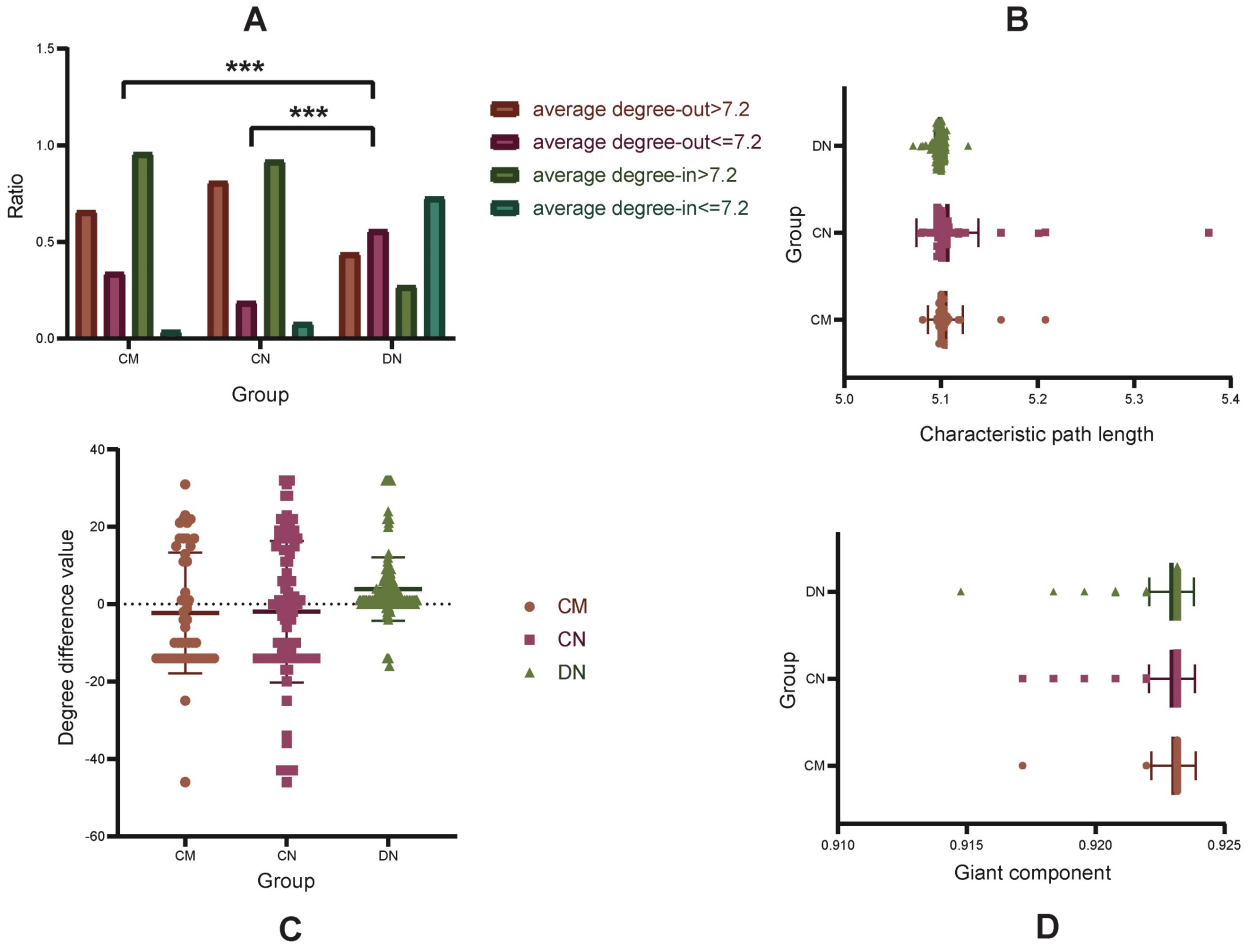
** Network analysis of CMs, CNs, and DNs. A.** The distribution probability of 4 levels of the HNS. **B.** Comparison of the changes in the characteristic path length after the nodes in the CM, CN, and DN sets are deleted. **C.** Comparison of the degree difference values in the CMs, CNs, and DNs.** D.** Comparison of the changes in the giant component after the nodes in the CM, CN, and DN sets are deleted.

To show the (out- and in-) degree differences between nodes in the HNS, we chose to take an average network degree of 7.2 as the baseline and set up four standards: average out-degree > 7.2, average out-degree ≤ 7.2, average in-degree > 7.2, and average in-degree ≤ 7.2 (Fig. 2A). Through comparative analysis, it was found that the driver nodes had a smaller in-degree (27% average in-degree > 7.2), which was significantly different from the module nodes and the core nodes (p<0.05). Moreover, by comparing the differences in the values of the out-degrees and in-degrees of the three node sets, it was shown that the percent of nodes with a degree difference ≥ 0 was 47% for CNs, 38% for CMs, and 97% for DNs (Supplementary Table S1, Fig. 2C).

### The characteristic path lengths and giant components of HNSs reveal the advantages of multitarget combinations

We deleted single-gene and random polygene combinations and used the characteristic path length and giant components to evaluate the importance and robustness of the deleted genes for the network. The results showed that after deleting the genes (deleting only one node at a time) in the three HNSs, only a few nodes can cause disturbances in the network (Fig. 2B, 2D). It is worth noting that only one of these perturbed genes was consistent with a list of lung cancer drug targets and biomarkers, so these genes may become new candidate targets for lung adenocarcinoma.

**Figure 3:**
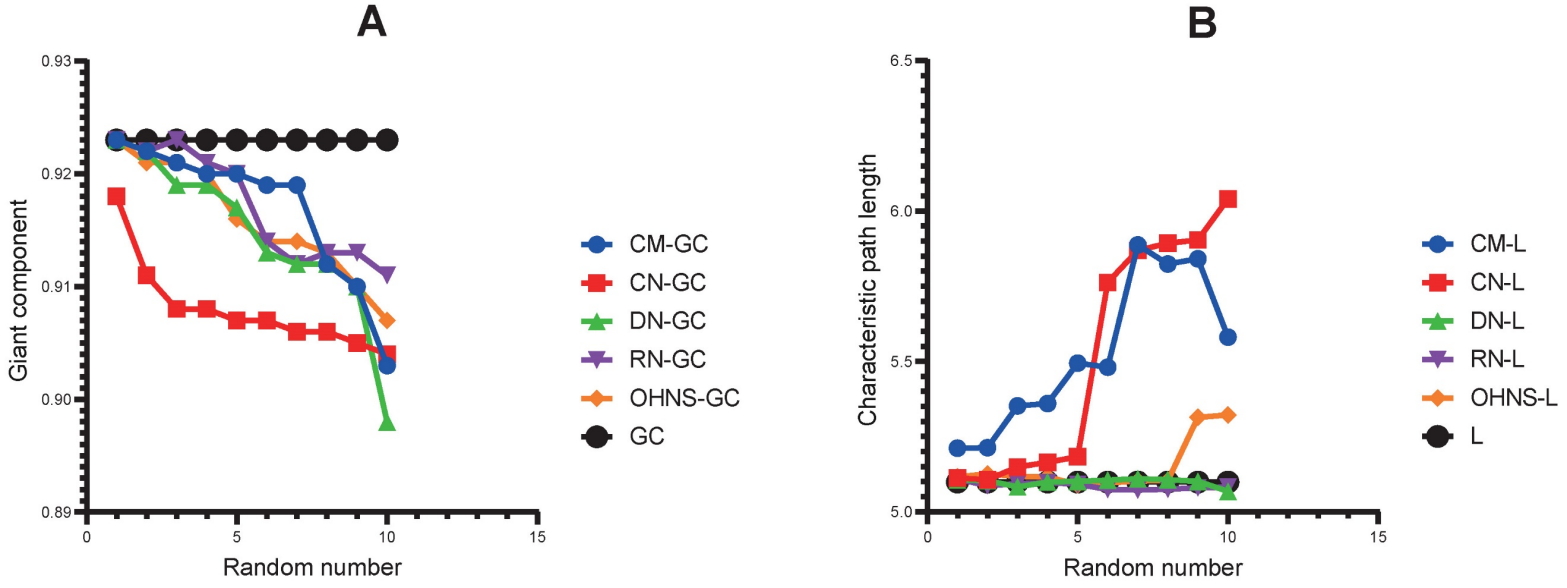
Performance comparisons of the CMs, CNs, DNs and OHNS of the network. **A.** The disturbance of the CMs, CNs, DNs, OHNS and RNs on the network; the smaller the giant component is, the greater the disturbance of the network.** B.** The importance of the CMs, CNs, DNs, OHNS and RNs in the network; the larger the characteristic path length is, the greater the importance.

As shown in Fig. 3, after deleting the random polygene combinations in turn (see Methods), the three different node sets are more obvious than the sets of random nodes. In the change trend of L (Fig. 3A), the L of the remaining network increases gradually when removing CMs (from 5.212 to 5.581) and CNs (from 5.112 to 6.041), which indicates that the CMs and the CNs play an important role in information transmission between the network nodes; after removing random DN combinations in turn, there is no change in the remaining network L.

The change trend of the GC is different from that of L; when the combinations of random nodes are deleted in turn, the GC of the remaining network shows a gradual shrinking trend. The GC of the remaining network without CNs is smaller than that of the other nodes. After removing the CM set (8^th^ iteration) and the DN set (10^th^ iteration), the GC of the remaining network has a very obvious and rapid shrinking trend (from 0.919 to 0.912 when removing the CMs, and from 0.910 to 0.898 when removing the DNs), which may be caused by the synergy of the node sets between networks (Fig. 3B).

### The characteristic path lengths and giant components are specific in a network

**Figure 4:**
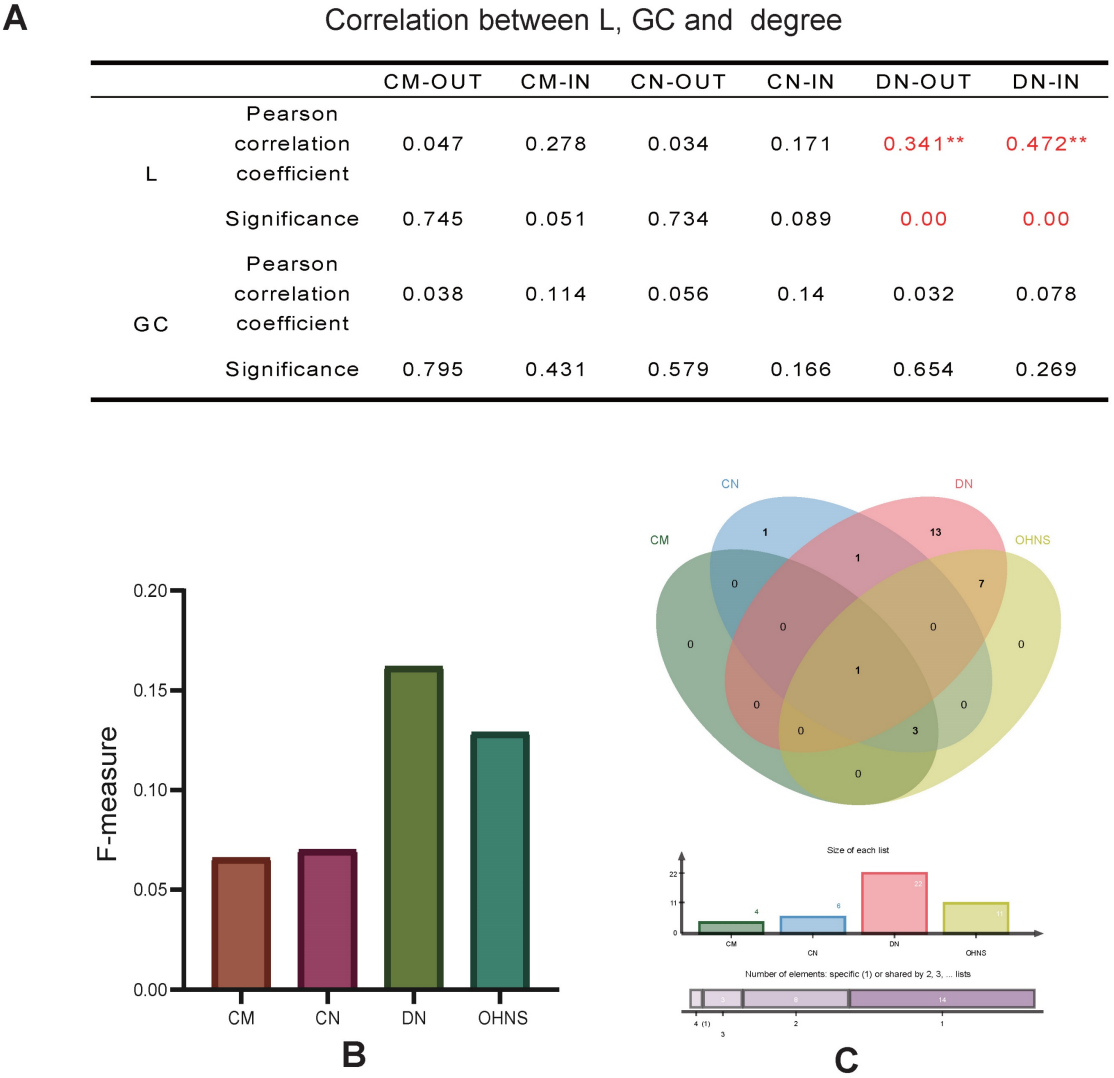
** Correlation and F-measure. A.** The correlation between the (out- and in-) degrees, the characteristic path length and the giant component in the CMs, CNs, and DNs. **B.** The F-measure of genes overlapping with the CMs, CNs, DNs and OHNS in the CTD list. **C.** Comparison of the overlapping genes with the CMs, CNs, DNs and OHNS in the CTD list.

To study whether the (in- and out-) degrees of different node sets are an influencing factor of the L and GC, we calculated the Pearson correlation coefficient between them. The results show that there is only a significant weak correlation between the (in- and out-) degrees of the driver nodes and L (Fig. 4A).

### The OHNS has better target prediction ability in terms of the F-measure and perturbation effect

The F-measure was used to evaluate the target prediction ability of the HNS in the list of drug targets and biomarker genes for lung cancer. We mapped lung cancer biomarkers and therapeutic targets from the CTD database to the CMs, CNs, and DNs and obtained 4, 6, and 22 markers and therapeutic targets, respectively. These genes are all supported by different studies. The DNs are more effective for discovering drug targets and biomarkers than the CMs and CNs (Fig. 4B). The results may also depend on the advantage of the absolute number of DNs. Consistent with the above observation, the mapping results of the CNs and CMs overlap. It is worth noting that the F-measure of the OHNS (see Methods) is 0.129, which is close to that of the DNs (Fig. 4B). However, only one pair of mapping results of the DNs and CMs overlap (Fig. 4C), so merging them can increase the hit probability of drug targets and biomarkers.

Using genome-scale CRISPR-Cas9 knockout data, we calculated the Chronos dependency scores of CMs, CNs, and DNs in 197 non-small cell lung cancer cell lines. Only 13 genes (CDC45, CDK1, CHEK1, ESPL1, MCM2, MCM4, MCM6, ORC1, ORC6, PKMYT1, PLK1 and PLK4) among the DNs had obvious perturbation effects on 197 non-small cell lung cancer cell lines (Supplementary Table S4). Interestingly, these 13 genes are also present in the OHNS.

### The OHNS enriched 3 unique pathways

**Figure 5:**
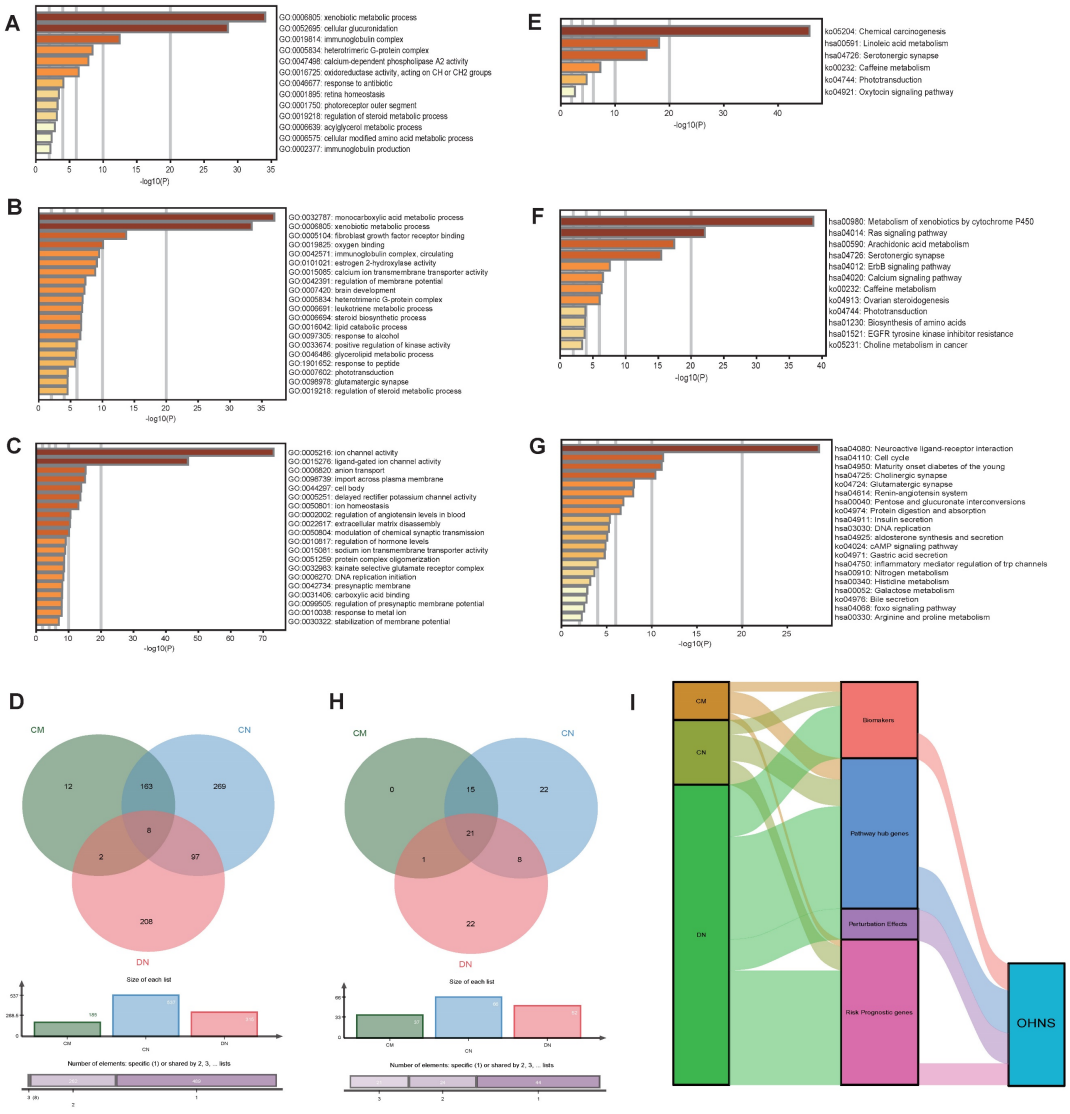
** Analysis of the CMs, CNs, DNs and OHNS. A-D.** The GO biological functions of CMs, CNs, and DNs by enrichment analysis. e-h. KEGG analysis of CMs, CNs, and DNs by enrichment analysis. A-C. Enrichment analysis of the top 20 GO biological functions in the CMs, CNs, and DNs.** E-G.** Enrichment analysis of the top 20 KEGG pathways in the CMs, CNs, and DNs. d. Biological process comparison of CMs, CNs, and DNs. **H.** Pathway comparison of CMs, CNs, and DNs. **I.** Comparison of the distributions of the CMs, CNs, DNs, and OHNS in terms of biomarkers, pathway hub genes, risk-prognostic genes, and perturbation effects. The width of the extended branch in the figure corresponds to the size of the data flow.

**Figure 6:**
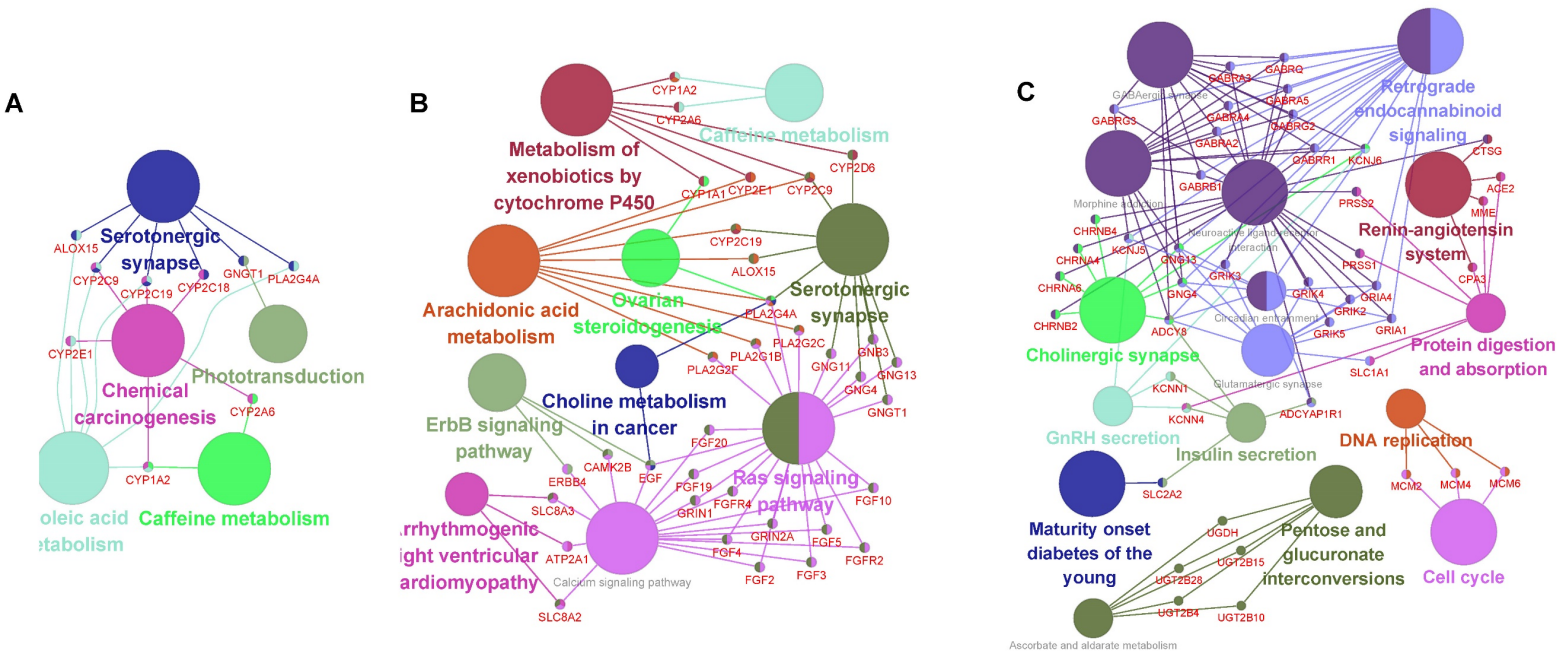
** Pathway link network of CMs, CNs and DNs.** The large nodes in the network represent the enriched pathways, the small nodes represent the genes between the linked pathways, the connected edges represent the common genes between the pathways, and the colors of the nodes represent the enrichment classification. A. Relationship between the enriched pathways and genes in the CMs. B. Relationship between the enriched pathways and genes in the CNs. C. Relationship between the enriched pathways and genes in the DNs.

Metascape was utilized to analyze the functions of the HNS. A total of 185, 537, and 315 GO functional enrichments (Fig. 5A-C) and 37, 66, and 52 KEGG pathways (Fig. 5E-G) were detected in the CMs, CNs, and DNs, respectively. In the results, there were 8 functional overlaps and 21 pathway overlaps of CMs, CNs and DNs. Interestingly, although the CMs and CNs overlap, they have different biological functions, which may be due to the number of genes not shared between them (Fig. 5D). We also used Cytoscape-ClueGO to create a pathway network and visualized the link genes between different pathways. The results showed that the CMs, CNs, and DNs had 9, 33, and 43 pathway hub genes, respectively (Fig. 6). However, in the KEGG pathway enrichment, the pathway to which the CNs are enriched contains the pathways of all CMs (Fig. 5H). There were 53 pathways enriched in the OHNS (Supplementary Figure S1). After comparison with the CMs and DNs, 3 unique pathways were discovered in the OHNS: the p53 signaling pathway, gap connection, and HTLV-I infection.

### Survival analysis further suggests the importance of 13 perturbed genes

The prognostic factors in the whole genome were observed, and the prognostic significance was evaluated by the single-factor Cox and log rank tests according to the expression level of a single gene. The results showed that there were a total of 2416 risk-prognosis-related genes, of which 3, 11, and 48 were found among the CMs, CNs, and DNs, respectively (Supplementary Table S5). Interestingly, we found that of the 48 DN risk prognostic genes, 13 were fully consistent with perturbed genes.

### Replication and validation analyses

By replicating this method to process the GEO data, a total of 3170 differentially expressed genes in lung adenocarcinoma were obtained. We constructed a set of 933 nodes and 4336 directed edges. After analyzing the network, we found that the degree distribution of CMs, CNs, and DNs in the GEO network, the degree difference, the F-measure and the L and GC scores after deleting a single node were almost identical to the trends of TCGA (Supplementary Figure S2, Table S6).

## Discussion

Choosing an effective HNS at the network level is a popular topic in the postgenome era, and it can help us obtain information about the key components of a disease network. An increasing number of studies about methods have been proposed due to the good theory and application value of identifying HNSs. These methods can be considered from two perspectives: detecting the core structure of the network and considering the ability to control the network. Given the important role that nodes with a high degree (hubs) have in maintaining the structural integrity of networks against failures and attacks 27, in spreading phenomena 28 and in synchronization 29, it is natural to expect that controlling the hubs is essential to controlling disease networks. Some hub nodes are not suitable as drug targets since they result in lethality or infertility. Predicting drug targets based on network control theory is a popular field, but completely controlling disease networks is currently difficult in the real world. Additionally, researchers do not fully understand the core connotations and characteristics of different types of HNS. They have only explored them from a certain angle, which has both advantages and limitations. To overcome these difficulties, we compare these approaches from different perspectives, such as core structure, control forces and clinical value, and show their respective characteristics.

The results show that DNs have advantages in risk prognosis, pathway hub genes, biomarkers, and perturbation effects. Therefore, we infer that DNs are likely to play a controlling role through the coordination of these four aspects (Fig. 5I). Considering the core structure and control capabilities of the network structure, we generated the OHNS. The F-measure shows that the OHNS increased in comparison with the CMs and CNs, and it changed in almost the same way as the DNs, which tells us that a combination of multiple calculation methods may increase the probability of disease control. This may be because the OHNS is based on the conservative evolution of core genes and plays a controlling role in the network. Thirteen genes in the OHNS were predicted to play roles in the risk prognosis, biomarkers, and perturbation effects of lung adenocarcinoma. Since RFC4 is a known drug target, the remaining 12 may be new biomarker candidates of drugs for lung adenocarcinoma. OHNS enrichment analysis showed the same results. The pathways enriched by the OHNS were not completely absorbed by the CM, CN and DN pathways. Instead, 3 unique pathways emerged. These may be signaling pathways in the regulatory network of lung adenocarcinoma that take into account both the structural centrality and control ability. Sun et al. 30 found that p53 signaling pathway inhibition by pifithrin-α abrogated tumor-suppressive effects in lung cancer. Maynard et al. 31, using 49 clinical biopsies obtained from 30 patients before and during targeted therapy, found that lung cancer progression was associated with upregulated gap-junction pathways. Recently, Flávia et al. 32 described a case of a small cell lung epidermoid carcinoma in a patient who developed HAM from HTLV-1 infection. This was the first case of this type of lung cancer since it was reported by Matsuzaki et al. 33 in 1990.

Cheng et al. 34 proposed a network-based approach to reveal that clinical drug combinations can have better efficacy according to the target distribution of two drugs in the protein interaction network. Gates et al. 35, in an effective graph study, explained why a combination of drugs could drive cancer proliferation to zero in this model while a single drug could not, and they proposed that only a combination of interventions was capable of fully resolving the state of the proliferation variables. Multitarget drugs and drug combinations are more promising for achieving sustainable clinical outcomes and therefore are more likely to activate a cascade of multiple pathways to robustly perturb disease phenotypes. A full understanding of the meaning or characteristics of HNSs calculated by different methods can prompt us to choose the appropriate method and help explore the space of gene combinations more effectively to identify synergistic gene interactions based on network topology 36. Therefore, it is reasonable to use more than one kind of method from multiple perspectives, considering not only the core position of the network but also the control ability of the network.

Notably, this research method is not limited to lung adenocarcinoma; it has certain flexibility in setting parameters, and different methods can be selected and combined according to research requirements so that researchers can change the method according to their own goals. At the same time, this research idea requires a certain amount of data to calculate differential genes and build regulatory networks. Therefore, it is not suitable for the study of sparse networks due to the small amount of data. However, the relevant conclusions regarding the OHNS method in this work can provide strategies and ideas for other disease treatments and provide more insights into complex diseases from multiple perspectives.

## Conclusions

Taken together, we proposed the idea of OHNS target combination based on the core structure and control ability of the lung adenocarcinoma regulatory network and obtained 3 unique lung adenocarcinoma-related pathways. Additionally, 13 genes were predicted to play roles in the risk prognosis, disease drivers, and cell perturbation effects of lung adenocarcinoma, of which 12 may be candidates for new drugs and biomarkers of lung adenocarcinoma. Although further experimental studies are needed, our study shows that the OHNS contains multiple disease biomarkers and therapeutic targets that can guide the therapeutic community to optimize appropriate strategies according to different cancer treatment targets, providing new avenues for disease intervention and drug discovery.

## Supplementary Material

Supplementary tables.
