# Supplementary material for: Optimizing Genomic Control in Hit Network-Target Set Model Associations with Lung Adenocarcinoma
Source: J Cancer. 2023 Jan 1;14(1):129–39. doi: 10.7150/jca.78138 (PMC9809338; doi:10.7150/jca.78138)
Supplement: Supplementary file 1 — Supplementary tables. [file jcav14p0129s1.pdf]

Supplementary Table S1.In-degree, out-degree and difference between in-degree and out-degree of three HNS											
**CM**	**degree-out**	**degree-in**	**difference value**	**CN**	**degree-out**	**degree-in**	**difference value**	**DN**	**degree-out**	**degree-in**	**difference value**
ALOX15	17	14	3	ACTN2	8	51	-43	ACE	1	0	1
AOC3	6	12	-6	ALOX15	17	14	3	ACE2	1	0	1
CACNA2D2	77	46	31	AOC3	6	12	-6	ACY3	5	0	5
CAMK2B	10	56	-46	ATP2A1	1	37	-36	ADAM12	1	0	1
CNGA1	47	36	11	CACNA2D2	77	46	31	ADCY8	8	4	4
CNGB1	47	36	11	CAMK2B	10	56	-46	ADCYAP1R1	1	0	1
CYP1A1	36	21	15	CNGA1	47	36	11	AGMAT	2	0	2
CYP1A2	36	21	15	CNGB1	47	36	11	AKR1B10	2	0	2
CYP2A6	8	9	-1	CYP1A1	36	21	15	ALOX5	4	1	3
CYP2C18	41	24	17	CYP1A2	36	21	15	ANO2	8	7	1
CYP2C19	41	24	17	CYP2A6	8	9	-1	ANO7	8	7	1
CYP2C9	41	24	17	CYP2B6	18	17	1	AOC1	2	2	0
CYP2D6	21	8	13	CYP2C18	41	24	17	ASNS	3	0	3
CYP2E1	36	38	-2	CYP2C19	41	24	17	ASPA	5	0	5
CYP3A7	36	19	17	CYP2C9	41	24	17	BDNF	2	2	0
GNB3	36	14	22	CYP2D6	21	8	13	CA5A	4	1	3
GNG11	26	5	21	CYP2E1	36	38	-2	CA7	3	1	2
GNG13	27	5	22	CYP2F1	12	26	-14	CA9	4	7	-3

GNG4	35	14	21	CYP3A7	36	19	17	CACNA1E	38	6	32
GNGT1	35	12	23	CYP4B1	8	8	0	CACNB4	38	6	32
GSTA3	5	9	-4	CYP4F11	8	8	0	CATSPERB	38	6	32
GSTM5	5	9	-4	CYP4F2	8	8	0	CATSPERD	38	6	32
IGHG1	11	25	-14	CYP4F3	8	8	0	CDC25C	4	3	1
IGHG2	11	25	-14	CYP4F8	8	8	0	CDC45	1	1	0
IGHG3	11	25	-14	EGF	25	6	19	CDK1	31	9	22
IGHG4	11	25	-14	ERBB4	21	7	14	CDX2	1	0	1
IGKC	16	26	-10	EREG	22	2	20	CHEK1	6	0	6
IGKV1-5	16	26	-10	FGF10	14	25	-11	CHEK2	3	1	2
IGKV4-1	16	26	-10	FGF19	19	23	-4	CHRNA2	7	6	1
IGLC2	16	26	-10	FGF2	13	25	-12	CHRNA4	16	15	1
IGLC3	16	26	-10	FGF20	13	25	-12	CHRNA5	7	6	1
IGLC6	16	26	-10	FGF3	12	25	-13	CHRNA6	7	6	1
PLA2G1B	11	10	1	FGF4	13	25	-12	CHRNA9	7	6	1
PLA2G2C	11	10	1	FGF5	12	25	-13	CHRNB2	16	15	1
PLA2G2F	11	10	1	FGFR2	11	31	-20	CHRNB4	16	15	1
PLA2G4A	10	35	-25	FGFR4	11	28	-17	CHRND	9	9	0
UGT1A1	3	17	-14	GNB3	36	14	22	CHRNG	9	9	0
UGT1A10	3	17	-14	GNG11	26	5	21	CMA1	2	0	2
UGT1A3	3	17	-14	GNG13	27	5	22	CNGA3	2	1	1
UGT1A6	3	17	-14	GNG4	35	14	21	CNGB1	47	36	11
UGT1A7	3	17	-14	GNGT1	35	12	23	CNGB3	2	1	1
UGT1A8	3	17	-14	GPT	12	14	-2	CPA3	1	0	1
UGT2A1	3	17	-14	GPT2	12	14	-2	CPB2	1	0	1

UGT2A3	3	17	-14	GRIN1	8	51	-43	CRP	12	0	12
UGT2B10	3	17	-14	GRIN2A	8	51	-43	CTSG	5	0	5
UGT2B11	3	17	-14	GRIN2D	8	51	-43	CXCL13	2	0	2
UGT2B15	3	17	-14	GSTA3	5	9	-4	CYP2A6	8	9	-1
UGT2B28	3	17	-14	GSTM5	5	9	-4	DAO	5	0	5
UGT2B4	3	17	-14	HBEGF	22	4	18	E2F7	3	0	3
XDH	0	10	-10	HSD17B2	26	9	17	EDNRB	2	0	2
				HSD17B6	26	9	17	EGLN3	1	0	1
				HSD3B1	32	13	19	ENPP3	5	0	5
				HSD3B2	32	13	19	EPHB1	2	4	-2
				HTR3A	89	57	32	ESPL1	24	0	24
				HTR3B	89	57	32	FEN1	1	0	1
				IGHG1	11	25	-14	FFAR4	1	0	1
				IGHG2	11	25	-14	FOSB	3	0	3
				IGHG3	11	25	-14	FOXA3	7	1	6
				IGHG4	11	25	-14	FTCD	1	1	0
				IGKC	16	26	-10	GABRA2	8	7	1
				IGKV1-5	16	26	-10	GABRA3	8	7	1
				IGKV4-1	16	26	-10	GABRA4	8	7	1
				IGLC2	16	26	-10	GABRA5	8	7	1
				IGLC3	16	26	-10	GABRB1	8	7	1
				IGLC6	16	26	-10	GABRG2	8	7	1
				IL4I1	12	10	2	GABRG3	8	7	1
				KL	19	36	-17	GABRQ	8	7	1
				NEFL	8	51	-43	GABRR1	8	7	1

				NME1	20	14	6	GATA1	7	0	7
				NRG1	24	2	22	GATA2	1	0	1
				NRG3	22	2	20	GCGR	4	0	4
				PC	8	9	-1	GLRA3	8	7	1
				PCK1	10	13	-3	GLRA4	8	7	1
				PIP5K1B	45	17	28	GNG13	27	5	22
				PIP5KL1	45	17	28	GNG4	35	14	21
				PKLR	17	13	4	GRIA1	26	17	9
				PLA2G1B	11	10	1	GRIA4	26	17	9
				PLA2G2C	11	10	1	GRIK2	16	15	1
				PLA2G2F	11	10	1	GRIK3	7	6	1
				PLA2G4A	10	35	-25	GRIK4	7	6	1
				SLC24A2	57	49	8	GRIK5	7	6	1
				SLC24A4	54	48	6	GSTM5	5	9	-4
				SLC8A2	52	46	6	GUCA1A	7	0	7
				SLC8A3	52	46	6	GUCY2C	7	1	6
				STX1A	51	37	14	HCN3	9	11	-2
				UGT1A1	3	17	-14	HES6	2	0	2
				UGT1A10	3	17	-14	HNF1A	8	0	8
				UGT1A3	3	17	-14	HNF4A	6	1	5
				UGT1A6	3	17	-14	IGHD	4	3	1
				UGT1A7	3	17	-14	IL12RB2	2	0	2
				UGT1A8	3	17	-14	IL4I1	12	10	2
				UGT2A1	3	17	-14	INHA	2	0	2
				UGT2A3	3	17	-14	INHBE	2	0	2

				UGT2B10	3	17	-14	KAT2A	2	0	2
				UGT2B11	3	17	-14	KCNA7	9	9	0
				UGT2B15	3	17	-14	KCNAB1	9	9	0
				UGT2B28	3	17	-14	KCNC1	9	9	0
				UGT2B4	3	17	-14	KCNC2	9	9	0
				WASF3	0	34	-34	KCND2	9	9	0
				XDH	0	10	-10	KCNF1	9	9	0
								KCNG1	9	9	0
								KCNG2	9	9	0
								KCNH1	9	9	0
								KCNH2	9	9	0
								KCNH7	9	9	0
								KCNH8	9	9	0
								KCNJ1	9	9	0
								KCNJ10	9	9	0
								KCNJ4	9	9	0
								KCNJ5	9	9	0
								KCNJ6	9	9	0
								KCNK10	9	9	0
								KCNK2	9	9	0
								KCNK3	9	9	0
								KCNK9	9	9	0
								KCNMB2	9	10	-1
								KCNN1	9	9	0
								KCNN4	9	9	0

								KCNQ2	9	9	0
								KCNQ3	9	9	0
								KCNQ5	9	9	0
								KCNV1	9	9	0
								KIF20A	5	1	4
								LCT	9	0	9
								LTC4S	2	2	0
								MCM10	1	0	1
								MCM2	1	2	-1
								MCM4	1	1	0
								MCM6	1	1	0
								MME	1	0	1
								MMP10	3	1	2
								MMP11	1	0	1
								MMP13	5	2	3
								MMP3	11	1	10
								MMP7	5	2	3
								MS4A2	2	0	2
								MTMR7	2	0	2
								MYBL2	4	0	4
								NEK2	1	1	0
								NEUROD1	4	1	3
								NEUROG3	3	2	1
								NPR1	7	1	6
								NR1H4	16	9	7

								NRG1	24	2	22
								NRG3	22	2	20
								ONECUT1	3	1	2
								ORC1	1	3	-2
								ORC6	1	1	0
								PAFAH1B3	5	0	5
								PCK1	10	13	-3
								PCSK1	3	0	3
								PF4	3	0	3
								PKLR	17	13	4
								PKMYT1	6	1	5
								PLCZ1	4	2	2
								PLK1	19	10	9
								PLK4	1	0	1
								PPARG	4	0	4
								PRKCE	3	0	3
								PRSS1	7	0	7
								PRSS2	3	0	3
								PTGDS	1	0	1
								QPRT	3	0	3
								RFC4	6	0	6
								RGR	5	0	5
								S1PR1	1	0	1
								SGK2	1	0	1
								SH3GL2	1	0	1

								SI	10	0	10
								SLC13A5	15	9	6
								SLC17A1	15	6	9
								SLC1A1	20	14	6
								SLC2A1	4	3	1
								SLC2A2	4	3	1
								SLC34A3	15	6	9
								SLC5A9	19	11	8
								SLC7A11	2	0	2
								SLC9A2	12	6	6
								SLC9A4	12	6	6
								SRMS	8	0	8
								SYN2	2	0	2
								SYT1	6	22	-16
								TEX15	2	0	2
								TPH2	2	0	2
								TPSAB1	1	0	1
								TRPA1	38	6	32
								TRPC5	38	6	32
								TRPM1	38	6	32
								TRPM8	38	6	32
								TRPV2	38	6	32
								TRPV3	38	6	32
								TTYH1	8	7	1
								TUBB4A	1	0	1

								UGDH	13	0	13
								UGT2B10	3	17	-14
								UGT2B15	3	17	-14
								UGT2B28	3	17	-14
								UGT2B4	3	17	-14
								VTN	1	0	1
								WNK2	1	0	1
								WNK3	1	0	1

Supplementary Table S2. Use HopcroftCKarp algorithm to get 396 drive nodes, and finally select 201 drive nodes based on control centrality		
ControlCentrality	MDS	name
239	TRUE	IL12RB2
239	TRUE	MS4A2
239	TRUE	CHEK1
238	TRUE	PKMYT1
238	TRUE	CDC25C
238	TRUE	TEX15
238	TRUE	E2F7
238	TRUE	MYBL2
238	TRUE	KAT2A
237	TRUE	NEK2
237	TRUE	ESPL1
237	TRUE	TUBB4A
237	TRUE	PLK4
236	TRUE	CDK1
236	TRUE	KIF20A
236	TRUE	CHEK2
235	TRUE	PLK1
220	TRUE	HNF1A
220	TRUE	PRSS1
220	TRUE	RFC4
219	TRUE	HNF4A
219	TRUE	GATA2
219	TRUE	MMP3
219	TRUE	SLC7A11
218	TRUE	ONECUT1
218	TRUE	HES6
218	TRUE	MMP7
218	TRUE	CTSG
218	TRUE	MMP10
218	TRUE	FOSB
218	TRUE	PF4
218	TRUE	TPSAB1
218	TRUE	EGLN3
218	TRUE	PRKCE
218	TRUE	MMP11
218	TRUE	CMA1
218	TRUE	ASNS
218	TRUE	PRSS2
217	TRUE	NEUROG3

217	TRUE	ORC6
217	TRUE	MCM6
217	TRUE	ADCY8
217	TRUE	MCM4
217	TRUE	MCM2
217	TRUE	MCM10
217	TRUE	ADCYAP1R1
217	TRUE	SYN2
217	TRUE	ANO7
217	TRUE	ORC1
217	TRUE	CDC45
217	TRUE	ANO2
217	TRUE	GCGR
217	TRUE	ADAM12
217	TRUE	TTYH1
217	TRUE	GLRA4
217	TRUE	GLRA3
217	TRUE	GABRR1
217	TRUE	GABRQ
217	TRUE	GABRG3
217	TRUE	GABRG2
217	TRUE	GABRB1
217	TRUE	GABRA5
217	TRUE	GABRA4
217	TRUE	GABRA3
217	TRUE	GABRA2
217	TRUE	SI
217	TRUE	NPR1
217	TRUE	ACE
217	TRUE	ACE2
217	TRUE	MMP13
217	TRUE	GUCY2C
217	TRUE	GUCA1A
217	TRUE	SRMS
217	TRUE	CPB2
217	TRUE	CPA3
217	TRUE	GATA1
217	TRUE	MME
217	TRUE	FEN1
217	TRUE	LCT
217	TRUE	RGR
217	TRUE	SH3GL2
217	TRUE	EDNRB

217	TRUE	DAO
217	TRUE	AKR1B10
216	TRUE	FOXA3
216	TRUE	SLC2A2
216	TRUE	SLC1A1
216	TRUE	SLC13A5
216	TRUE	NEUROD1
216	TRUE	NR1H4
216	TRUE	GRIK2
216	TRUE	GRIA4
216	TRUE	GRIA1
216	TRUE	CHRNG
216	TRUE	CHRND
216	TRUE	CHRNB4
216	TRUE	CHRNB2
216	TRUE	CHRNA4
216	TRUE	BDNF
216	TRUE	PAFAH1B3
216	TRUE	CA9
216	TRUE	SLC2A1
216	TRUE	EPHB1
216	TRUE	GNG4
216	TRUE	GNG13
216	TRUE	SLC5A9
216	TRUE	FTCD
216	TRUE	PPARG
216	TRUE	CDX2
216	TRUE	ACY3
216	TRUE	VTN
216	TRUE	ASPA
216	TRUE	MTMR7
216	TRUE	CRP
216	TRUE	IGHD
216	TRUE	TRPV3
216	TRUE	ENPP3
216	TRUE	TRPV2
216	TRUE	TRPM8
216	TRUE	TRPM1
216	TRUE	TRPC5
216	TRUE	TRPA1
216	TRUE	UGDH
216	TRUE	GRIK5
216	TRUE	GRIK4

216	TRUE	GRIK3
216	TRUE	CHRNA9
216	TRUE	CHRNA6
216	TRUE	CHRNA5
216	TRUE	CHRNA2
216	TRUE	CATSPERD
216	TRUE	CATSPERB
216	TRUE	CA7
216	TRUE	CA5A
216	TRUE	CACNB4
216	TRUE	CACNA1E
216	TRUE	HCN3
216	TRUE	KCNMB2
216	TRUE	SLC9A4
216	TRUE	SLC9A2
216	TRUE	SLC34A3
216	TRUE	FFAR4
216	TRUE	SLC17A1
216	TRUE	PCSK1
216	TRUE	KCNV1
216	TRUE	KCNQ5
216	TRUE	KCNQ3
216	TRUE	KCNQ2
216	TRUE	KCNN4
216	TRUE	KCNN1
216	TRUE	KCNK9
216	TRUE	KCNK3
216	TRUE	KCNK2
216	TRUE	KCNK10
216	TRUE	KCNJ6
216	TRUE	KCNJ5
216	TRUE	KCNJ4
216	TRUE	KCNJ1
216	TRUE	PLCZ1
216	TRUE	NRG3
216	TRUE	NRG1
216	TRUE	KCNJ10
216	TRUE	KCNH8
216	TRUE	KCNH7
216	TRUE	AGMAT
216	TRUE	KCNH2
216	TRUE	KCNH1
216	TRUE	KCNG2

216	TRUE	KCNG1
216	TRUE	KCNF1
216	TRUE	KCND2
216	TRUE	KCNC2
216	TRUE	KCNC1
216	TRUE	KCNAB1
216	TRUE	KCNA7
216	TRUE	INHBE
216	TRUE	INHA
216	TRUE	QPRT
216	TRUE	WNK3
216	TRUE	WNK2
216	TRUE	SGK2
216	TRUE	TPH2
216	TRUE	S1PR1
215	TRUE	IL4I1
215	TRUE	PCK1
215	TRUE	PKLR
215	TRUE	CNGB1
215	TRUE	SYT1
215	TRUE	CXCL13
215	TRUE	AOC1
215	TRUE	CYP2A6
215	TRUE	GSTM5
215	TRUE	UGT2B28
215	TRUE	UGT2B4
215	TRUE	UGT2B15
215	TRUE	UGT2B10
215	TRUE	ALOX5
215	TRUE	CNGB3
215	TRUE	CNGA3
214	TRUE	LTC4S
214	TRUE	PTGDS
6	TRUE	THBD
6	TRUE	SERPINE2
6	TRUE	SERPINA5
5	TRUE	AURKB
5	TRUE	NKX2-2
5	TRUE	B3GALT5
5	TRUE	AWAT2
5	TRUE	CPT1B
5	TRUE	CPN1
5	TRUE	PCSK2

4	TRUE	EPHA8
4	TRUE	EPHA6
4	TRUE	EPHA5
4	TRUE	GALNTL6
4	TRUE	GALNTL5
4	TRUE	EPHA10
4	TRUE	GALNT7
4	TRUE	GALNT6
4	TRUE	CLPS
4	TRUE	GALNT4
4	TRUE	GALNT14
4	TRUE	GALNT13
4	TRUE	RSPO1
3	TRUE	MOGAT3
3	TRUE	DKK4
3	TRUE	DKK2
3	TRUE	CAV1
3	TRUE	PYCR1
3	TRUE	HOXB13
3	TRUE	UBE2T
3	TRUE	PNLIPRP3
3	TRUE	PNLIPRP1
3	TRUE	FHL2
3	TRUE	KNG1
3	TRUE	LIPF
3	TRUE	EGR1
3	TRUE	SEMA3A
3	TRUE	SPDEF
3	TRUE	KREMEN2
3	TRUE	RXRG
3	TRUE	CMTM2
3	TRUE	IL23A
3	TRUE	TLL2
3	TRUE	TLL1
3	TRUE	HMOX1
2	TRUE	RACGAP1
2	TRUE	ATP6V1C2
2	TRUE	ATP6V0A4
2	TRUE	GH2
2	TRUE	ECT2
2	TRUE	CYP27A1
2	TRUE	PLAU
2	TRUE	DLC1

2	TRUE	CYP27B1
2	TRUE	DEPDC7
2	TRUE	DEPDC1B
2	TRUE	SLCO1B3
2	TRUE	SLCO1B1
2	TRUE	SLCO1A2
2	TRUE	GCNT3
2	TRUE	TGFBR2
2	TRUE	ADAMTS14
2	TRUE	CFD
2	TRUE	GCLC
2	TRUE	WIF1
2	TRUE	HPSE2
2	TRUE	PIF1
2	TRUE	ABCC3
2	TRUE	STARD8
2	TRUE	STARD13
2	TRUE	A2M
2	TRUE	LDLR
2	TRUE	ST6GALNAC5
2	TRUE	ST6GALNAC3
2	TRUE	PLOD2
2	TRUE	GDA
2	TRUE	MND1
2	TRUE	SLC39A8
2	TRUE	SLC39A5
2	TRUE	SLC39A4
2	TRUE	ISG15
2	TRUE	MMP19
2	TRUE	ARHGEF16
2	TRUE	ARHGAP6
2	TRUE	POU4F1
2	TRUE	LRAT
2	TRUE	ARHGAP44
2	TRUE	PDIA2
2	TRUE	ARHGAP40
2	TRUE	PCOLCE2
2	TRUE	IL6
2	TRUE	ARHGAP36
2	TRUE	PTGES
2	TRUE	ARHGAP31
2	TRUE	KLK13
2	TRUE	NDST4

2	TRUE	NDST1
2	TRUE	HS6ST2
2	TRUE	B3GNT4
2	TRUE	HS3ST1
2	TRUE	CHST9
2	TRUE	CHST4
2	TRUE	B3GNT3
2	TRUE	TEK
2	TRUE	ARHGAP11A
2	TRUE	B3GAT1
2	TRUE	CIT
2	TRUE	MCF2
2	TRUE	APOF
2	TRUE	FOXH1
2	TRUE	PRSS3
2	TRUE	DIO2
2	TRUE	ABCA4
2	TRUE	DGKI
2	TRUE	DGKB
2	TRUE	ISL1
1	TRUE	C4BPB
1	TRUE	HNF4G
1	TRUE	NCAPH
1	TRUE	NDC80
1	TRUE	NCAPG
1	TRUE	KIF2C
1	TRUE	ITGA2
1	TRUE	TNNT1
1	TRUE	TNNI3
1	TRUE	TNNC2
1	TRUE	TNNC1
1	TRUE	TMOD1
1	TRUE	SYT5
1	TRUE	SORBS1
1	TRUE	ENTPD2
1	TRUE	BUB1B
1	TRUE	CLSPN
1	TRUE	TPX2
1	TRUE	SPC24
1	TRUE	CENPE
1	TRUE	UBE2C
1	TRUE	NCAPG2
1	TRUE	CDCA5

1	TRUE	CDC20
1	TRUE	ZWINT
1	TRUE	SPC25
1	TRUE	SKA1
1	TRUE	NUF2
1	TRUE	MAD2L1
1	TRUE	KNTC1
1	TRUE	ERCC6L
1	TRUE	CENPM
1	TRUE	CENPK
1	TRUE	TIMP1
1	TRUE	NTS
1	TRUE	LBP
1	TRUE	KRT5
1	TRUE	KRT17
1	TRUE	IL13
1	TRUE	NKX6-1
1	TRUE	SOX2
1	TRUE	TWIST1
1	TRUE	NPC1L1
1	TRUE	AQP9
1	TRUE	AQP10
1	TRUE	ABCA9
1	TRUE	ABCA3
1	TRUE	EZH2
1	TRUE	MUCL1
1	TRUE	MUC6
1	TRUE	COL5A2
1	TRUE	COL5A1
1	TRUE	COL3A1
1	TRUE	COL2A1
1	TRUE	COL24A1
1	TRUE	COL1A1
1	TRUE	CKM
1	TRUE	RGS9BP
1	TRUE	RGS9
1	TRUE	TACC3
1	TRUE	HMGA2
1	TRUE	APOC3
1	TRUE	GP9
1	TRUE	SYCP2
1	TRUE	SMC1B
1	TRUE	COL9A3

1	TRUE	COL25A1
1	TRUE	COL22A1
1	TRUE	COL20A1
1	TRUE	COL17A1
1	TRUE	C1QB
1	TRUE	RPE65
1	TRUE	RAPGEF4
1	TRUE	CBR3
1	TRUE	ELOVL2
1	TRUE	CRABP2
1	TRUE	F2RL3
1	TRUE	SULT4A1
1	TRUE	PPP1R3A

**Supplementary Table S3.** **Use MCODE, MCL and Connected Components to divide disease network modules and calculate entropy results**			
**Module**	**Entropy**		**Result**
mcl	5.98614		Clusters: 39 Average size: 20.923 Maximum size: 673 Minimum size: 2
mcode	5.10523		Clusters: 28 Average size: 8.143 Maximum size: 40 Minimum size: 3
cc	6.01836		Clusters: 15 Average size: 55.6 Maximum size: 770 Minimum size: 2

Supplementary Table S4. Obvious perturbation effects on 197 non-small cell lung cancer cell lines

depmap_id	cell_line_display_name	lineage_2	CDC45	CDK1	CHEK1	ESPL1	MCM2	MCM4	MCM6	ORC1	ORC6	PKMYT1	PLK1	PLK4	RFC4
ACH-002089	201T	NSCLC													
ACH-000757	A427	NSCLC	-2.149717985	-1.755794504	-1.823076406	-1.843102458	-1.454993497	-1.243026464	-1.312406747	-1.854627854	-1.737964386	-1.673903551	-2.52350676	-0.842483337	-0.96677792
ACH-000681	A549	NSCLC	-2.020249938	-2.097607576	-2.077016828	-2.226273139	-1.535209878	-1.237220521	-1.404240785	-1.113913535	-0.779907026	-1.166070079	-2.336719144	-1.242406204	-1.257758635
ACH-000528	ABC1	NSCLC	-2.198676196	-1.901820403	-2.214025861	-1.900585284	-1.704847694	-1.448656764	-1.477682857	-1.430796397	-1.817699153	-0.101211165	-2.41465789	-1.485613621	-1.129304419
ACH-000086	ACCMESO1	Mesothelioma	-2.218935862	-1.813052353	-2.0217332	-1.69078543	-2.155685137	-1.079247013	-1.339358323	-1.173315912	-1.695250291	-1.437049603	-2.309050295	-1.257600017	-1.460485275
ACH-000603	BEN	NSCLC													
ACH-000826	CAL12T	NSCLC	-2.475332064	-2.153392371	-1.491641261	-1.690109498	-1.577179645	-1.389757633	-1.356613228	-1.576680772	-1.533390409	-1.160197819	-2.388689174	-1.026981983	-1.209320488
ACH-000511	CALU1	NSCLC	-2.481051164	-1.892567256	-2.0750456	-1.61270253	-1.745707303	-1.734125054	-1.524663128	-1.397714939	-1.804599871	-1.641881306	-2.338796594	-0.642258612	-1.426356382
ACH-000392	CALU3	NSCLC													
ACH-000264	CALU6	NSCLC	-2.301465896	-2.164953992	-2.445902417	-1.806258801	-1.921071371	-1.599881528	-1.836198235	-1.28303758	-2.065183225	-0.738437449	-2.234238663	-1.09782874	-1.622729317
ACH-000867	CHAGOK1	NSCLC	-2.352406396	-2.115420904	-2.204409863	-2.155342478	-1.532907663	-1.566952983	-1.706875493	-1.175932069	-1.899030408	-1.838921797	-2.197095019	-0.509461201	-1.486492133
ACH-001041	COLO699	NSCLC	-2.103543737	-1.489884052	-2.073934045	-1.972127873	-1.486973624	-1.092805533	-1.252000582	-1.390891242	-1.791549504	-0.555800965	-2.277347513	-1.207265547	-0.97672596
ACH-000161	CORL105	NSCLC	-2.389531018	-1.99102221	-1.723105033	-1.919892847	-1.904274848	-1.48539171	-1.425723039	-1.317729862	-1.561472787	-0.672790571	-2.389367032	-1.089808994	-0.894247856
ACH-000662	CORL23	NSCLC	-2.104653034	-1.946914066	-1.90922521	-2.198054601	-2.380424287	-2.085212628	-1.454715657	-1.743379597	-1.810049975	-1.218230745	-2.439210356	-0.775724349	-1.021550263
ACH-001489	CORL32	NSCLC													
ACH-001490	CORL321	Lung Carcinoma													
ACH-000925	DV90	NSCLC													
ACH-000563	EBC1	NSCLC	-2.291519262	-2.104567147	-2.001602364	-1.561893471	-1.84966621	-1.589896595	-1.287671395	-0.902564314	-0.973107678	-0.576238736	-2.424687793	-0.996391899	-1.362674641
ACH-000706	EKVX	NSCLC	-2.099651803	-2.042498406	-2.272979321	-1.681978384	-1.529405549	-1.249215974	-1.465654924	-1.08510419	-1.288006557	-0.351766753	-2.360144057	-1.467763831	-1.125249125
ACH-002101	EMCBAC1	NSCLC													
ACH-002102	EMCBAC2	NSCLC													

ACH-000585	EPLC272H	NSCLC	-2.311749575	-2.276751274	-2.350927095	-2.030861204	-1.52386687	-1.193582358	-1.457125595	-1.400990974	-1.140714832	-1.954719146	-2.393489238	-1.233018672	-0.847464274
ACH-001071	GLC82	NSCLC													
ACH-002122	GT3TKB	NSCLC													
ACH-002123	H2369	Mesothelioma													
ACH-002124	H2373	Mesothelioma													
ACH-002125	H2461	Mesothelioma													
ACH-002126	H2591	Mesothelioma													
ACH-002127	H2595	Mesothelioma													
ACH-002128	H2722	Mesothelioma													
ACH-002129	H2731	Mesothelioma													
ACH-002130	H2795	Mesothelioma													
ACH-002131	H2803	Mesothelioma													
ACH-002132	H2804	Mesothelioma													
ACH-002133	H2810	Mesothelioma													
ACH-002134	H2818	Mesothelioma													
ACH-002135	H2869	Mesothelioma													
ACH-002136	H290	Mesothelioma													
ACH-002138	H513	NSCLC													
ACH-000843	HARA	NSCLC	-2.180779568	-1.820019047	-2.155867069	-2.110028351	-1.527660238	-1.522543196	-1.363959592	-1.536711171	-1.663218416	-1.283901535	-2.273637458	-1.313835486	-0.864891829
ACH-000578	HCC1171	NSCLC													
ACH-000868	HCC1195	NSCLC													
ACH-000901	HCC1359	NSCLC	-2.263350184	-2.376115634	-2.446639413	-1.67421333	-1.912306561	-1.56144175	-1.924411782	-1.166375367	-1.869509822	-1.239060274	-2.341308681	-0.967259722	-1.607996274
ACH-000891	HCC1438	NSCLC													
ACH-000878	HCC15	NSCLC	-2.430919562	-1.868444555	-1.768524592	-1.86693383	-1.776675355	-1.722300371	-1.701794906	-1.09489005	-1.408853894	-0.819400987	-2.363458313	-0.959759493	-1.067034617
ACH-001078	HCC1588	NSCLC													

ACH-000712	HCC1833	NSCLC													
ACH-001079	HCC1897	NSCLC													
ACH-000314	HCC2108	NSCLC													
ACH-000731	HCC2279	NSCLC													
ACH-000143	HCC2429	NSCLC	-2.022532634	-1.977688849	-2.151554977	-1.926344189	-1.727169948	-1.194976903	-1.012397379	-1.364504358	-1.934101929	-0.635578088	-2.366004368	-1.310822292	-0.592686147
ACH-000975	HCC2450	NSCLC	-2.349237481	-2.117278112	-2.333560847	-1.969968589	-1.849636444	-1.659179608	-1.678999559	-1.312208978	-1.692872858	-1.994793571	-2.130214126	-0.654910473	-1.476196492
ACH-000690	HCC2814	NSCLC													
ACH-000150	HCC2935	NSCLC	-2.405306081	-2.145932974	-1.897098945	-1.596631457	-1.723189686	-1.363754543	-1.683674955	-1.037544741	-2.006903412	-1.302277997	-2.169103553	-0.768550319	-1.695270016
ACH-000575	HCC364	NSCLC													
ACH-000840	HCC366	NSCLC	-2.469845449	-1.836554776	-1.743798154	-2.314127968	-1.629544613	-1.297592771	-1.750930991	-1.170939964	-1.410306386	-1.175973281	-2.655040554	-0.558719549	-1.216582775
ACH-000066	HCC4006	NSCLC													
ACH-000667	HCC44	NSCLC	-2.396316453	-1.738110841	-2.239555644	-2.195517778	-2.203257641	-1.781196039	-1.690482474	-1.003518057	-1.423518652	-1.035924271	-2.32363762	-0.522649184	-1.173082636
ACH-000339	HCC461	NSCLC	-2.201993371	-2.181733349	-2.272809371	-1.882710796	-2.049479779	-1.623410802	-1.70686226	-1.188934594	-1.495904828	-0.96751556	-2.17703271	-1.122978418	-1.098716229
ACH-000872	HCC515	NSCLC	-2.359797733	-1.874313682	-1.945737461	-1.911798206	-1.538598067	-1.382679919	-1.350370269	-1.641602186	-1.507899273	-1.381738537	-2.256206114	-1.431100912	-1.306339556
ACH-000562	HCC78	NSCLC	-2.377672455	-2.238782664	-1.137414829	-1.890597012	-1.849938536	-1.468777229	-1.280224417	-1.509831008	-1.325073421	-1.002005449	-2.22296588	-1.170627472	-1.195849704
ACH-000012	HCC827	NSCLC	-2.231337089	-1.669859613	-2.073773063	-1.943372628	-1.526340346	-1.160708698	-1.249523304	-1.123192801	-0.789500813	-1.723844589	-1.905480759	-1.35590526	-1.443111918
ACH-002333	HCC827GR														
ACH-000029	HCC827GR5	NSCLC	-2.214496207	-2.094237238	-2.184819289	-1.854603323	-1.593171239	-1.411090854	-1.540889071	-1.520778722	-1.110172385	-1.611301587	-2.222274867	-1.283969236	-1.20599916
ACH-000454	HCC95	NSCLC	-2.281944792	-2.006269173	-1.788383087	-2.260723132	-1.533589519	-0.968533249	-1.061755627	-0.957538423	-1.483379985	-1.427188958	-2.615991436	-0.839415351	-0.624431716
ACH-001088	HLC1	NSCLC													
ACH-000861	HOP62	NSCLC	-2.18558189	-2.154886897	-2.151014728	-1.805815655	-1.83648625	-1.141882801	-1.328717002	-1.020515203	-1.562251698	-0.285644312	-2.348332705	-0.896417729	-1.000416497
ACH-000825	HOP92	NSCLC													
ACH-000672	IALM	NSCLC													
ACH-000569	ISTMES1	Mesothelioma													
ACH-000331	ISTMES2	Mesothelioma	-2.275140501	-2.113857041	-1.493454879	-1.834113635	-1.783927788	-1.57617707	-1.233333216	-1.17299251	-1.19977917	-1.191832201	-2.422326872	-1.158076893	-1.281944657

ACH-002337	JHU028	NSCLC													
ACH-000645	JL1	Mesothelioma	-2.296428319	-1.449244492	-1.49884704	-1.665697702	-1.936721797	-1.468432581	-1.585222727	-1.199249109	-1.075958575	-0.630223587	-1.987887757	-0.853562592	-0.751415531
ACH-000858	KNS62	NSCLC	-2.44474893	-1.990196108	-2.139423997	-2.043745341	-1.898872391	-1.450146782	-1.57772362	-1.269878302	-1.75453052	-1.203282549	-2.305631358	-0.893450139	-0.892219912
ACH-000705	LC1F	NSCLC													
ACH-002156	LC1SQ	NSCLC	-2.376794552	-1.995899291	-2.324240167	-1.990756692	-2.009599547	-1.514198609	-1.798708469	-1.33505715	-1.656628737	-1.802997185	-2.628012784	-0.866690498	-0.778041488
ACH-001113	LC1SQSF	NSCLC													
ACH-002157	LC2AD	NSCLC													
ACH-000627	LCLC103H	NSCLC	-2.188619284	-2.022880085	-2.0602351	-1.994384048	-1.637162706	-1.210125345	-1.285240423	-1.465635478	-1.309377834	-1.33165356	-2.361324474	-0.806496337	-0.894705233
ACH-000596	LCLC97TM1	NSCLC	-2.327957889	-2.132283603	-2.09232602	-2.015919566	-1.855700584	-1.803385445	-1.752496851	-1.215831065	-1.342720147	-1.599019017	-1.994382285	-1.428615965	-1.205158523
ACH-000769	LK2	NSCLC	-2.210657746	-2.147651493	-1.915997126	-1.848230013	-1.604152881	-1.496201614	-1.226352533	-1.446959236	-1.815393113	-0.547069833	-2.391810706	-1.279484557	-1.217447189
ACH-001547	LO68	Mesothelioma	-1.364609852	-1.908037816	-2.345598017	-2.308921681	-1.375164018	-1.415909644	-1.521743428	-0.871454634	-1.399672537	-1.244392976	-2.130658469	-1.228875152	-0.707839758
ACH-000176	LOUNH91	NSCLC	-2.398874521	-2.294481393	-2.370125244	-2.217920721	-1.876433057	-1.793763654	-1.757577964	-1.693070586	-1.95138625	-1.741921766	-2.364084892	-0.70504083	-0.938700352
ACH-000438	LU65	NSCLC	-2.351351645	-2.064188222	-2.111566897	-1.617554136	-2.079541656	-1.550007449	-1.469986195	-1.075746903	-1.832190537	-1.393358061	-2.464271991	-1.284942476	-1.380313669
ACH-000444	LU99	NSCLC	-2.401237483	-2.204173232	-2.215300278	-2.082491442	-1.857020371	-1.179471942	-1.497894713	-1.287032134	-1.934508827	-1.032117529	-2.455258328	-1.117067874	-1.168722259
ACH-002158	LU99A	NSCLC													
ACH-000390	LUDLU1	NSCLC	-2.275107385	-1.992672531	-2.311982683	-1.809774281	-1.738348985	-1.54048621	-1.757843722	-1.106409385	-0.913673673	-2.100182812	-2.234804846	-1.459145248	-1.177228812
ACH-000787	LXF289	NSCLC	-2.220631482	-1.795333245	-2.03888126	-2.007198102	-1.872822388	-1.430422338	-1.810482434	-1.175301586	-1.70280174	-1.396378488	-2.537525797	-1.140006391	-0.954921419
ACH-001555	MERO14	Mesothelioma	-2.315908034	-1.798049544	-1.421682338	-1.951094689	-1.431498786	-1.983706435	-1.620212571	-0.691613189	-1.283127477	-2.050492142	-1.659289035	-1.322180314	-0.521749925
ACH-001556	MERO25	Mesothelioma	-2.124826178	-1.935478551	-2.18108198	-0.985115503	-1.782872797	-1.614016321	-1.613415175	-1.235698683	-1.564988136	-1.263123245	-2.424540213	-0.033574773	-1.193291312
ACH-001557	MERO41	Mesothelioma	-2.110328155	-2.181375514	-2.132282569	-2.027957818	-1.429647561	-1.559180528	-1.611343551	-1.251826934	-1.127620891	-1.432936327	-2.269656787	-1.236001388	-1.197021938
ACH-001558	MERO48A	Mesothelioma													
ACH-001559	MERO82	Mesothelioma	-2.690091936	-1.959758597	-1.611325329	-1.428468658	-1.522090772	-1.097481194	-1.861254007	-0.790329245	-1.616007858	-1.234853819	-2.451722325	-1.008165378	-1.407514687
ACH-001560	MERO83	Mesothelioma	-2.204226352	-2.149178169	-1.554813419	-2.046399826	-1.933128014	-1.533463493	-1.69577937	-1.192303514	-1.343967516	-1.189559534	-2.313076643	-1.13007354	-1.156315867
ACH-001561	MERO84	Mesothelioma													
ACH-001562	MERO95	Mesothelioma	-2.305939698	-1.563474065	-1.782509905	-2.218883746	-1.836997678	-1.306633835	-1.238875034	-0.945114818	-2.081741311	-0.771442594	-2.078272881	-1.102518374	-1.150956761

ACH-000851	MORCPR	NSCLC	-2.343941606	-2.0323688	-1.856083615	-2.252958433	-1.2663499	-1.454982104	-1.125791574	-0.81194792	-1.553410296	-1.038672889	-2.191438776	-1.27582614	-0.85972019
ACH-000319	MPP89	Mesothelioma	-2.327155308	-2.092006729	-1.922625926	-2.104263968	-1.657585064	-1.230000545	-1.715910467	-1.400213592	-1.914074139	-1.066814449	-2.289521129	-1.170540319	-1.226668708
ACH-000335	MSTO211H	Mesothelioma	-0.888805417	-1.310607796	-1.765517229	-0.963528495	-1.924926566	-1.147988007	-1.157066269	-1.027507489	-1.325168691	-0.889040717	-1.595024039	-0.614412308	-1.230582048
ACH-000980	NCIH1155	NSCLC	-2.430043195	-2.027286733	-2.306232004	-2.131996421	-1.824377308	-1.708484631	-1.707593604	-1.622431431	-1.88484839	-1.063044768	-2.464044557	-1.060755755	-1.159975141
ACH-000510	NCIH1299	NSCLC	-2.311690428	-1.883755739	-2.246586397	-2.071726137	-1.766351521	-1.309625905	-1.764329282	-1.615006303	-1.645865185	-1.71455922	-2.283314202	-1.339059155	-1.300449759
ACH-000666	NCIH1355	NSCLC	-2.260566025	-2.049637557	-2.153292626	-2.285377423	-1.789968047	-1.601225236	-1.250157217	-1.350592702	-1.162962994	-1.268635444	-2.356121572	-1.37090379	-0.837132371
ACH-000845	NCIH1373	NSCLC	-2.290544148	-1.092086943	-1.54503922	-2.01635877	-1.672452216	-1.160189118	-1.228054829	-1.688837263	-1.324155961	-1.191130162	-2.231646438	-0.863106384	-0.953422166
ACH-000737	NCIH1385	NSCLC													
ACH-000327	NCIH1395	NSCLC													
ACH-000852	NCIH1435	NSCLC													
ACH-000589	NCIH1437	NSCLC	-2.359064309	-2.46010707	-2.076885091	-2.301349844	-1.585834733	-1.385379539	-1.317407979	-0.95510664	-1.682307924	-1.335515624	-2.140129842	-0.834157489	-1.419655123
ACH-000892	NCIH1563	NSCLC													
ACH-000869	NCIH1568	NSCLC	-2.36363268	-2.200118649	-2.422462807	-1.558407676	-1.774126154	-1.219629129	-1.357184601	-1.518294693	-1.475725513	-1.240987014	-2.509402751	-1.481488075	-1.084578197
ACH-000916	NCIH1573	NSCLC	-2.176429354	-2.065103814	-1.583013447	-1.997287017	-1.432855007	-1.314632512	-1.909069634	-1.076787719	-1.309241659	-0.66853436	-2.391986733	-0.363713171	-0.912201935
ACH-000015	NCIH1581	NSCLC	-2.352198613	-2.081045668	-1.934601348	-2.135364134	-1.71043316	-1.526233228	-1.415964853	-1.178423211	-1.498948296	-0.815586634	-2.31691476	-1.227253676	-1.151644244
ACH-000744	NCIH1623	NSCLC													
ACH-000766	NCIH1648	NSCLC	-2.455217758	-2.259279909	-2.480556475	-1.392691644	-1.521863532	-1.676430075	-0.857337474	-0.920972952	-1.942002937	-1.378626929	-2.486889791	-0.621413548	-1.332969226
ACH-000035	NCIH1650	NSCLC	-2.392204551	-1.814487678	-2.135812287	-2.106693787	-1.698594826	-1.345040833	-1.774296597	-1.441706941	-1.651796121	-1.517333298	-2.335850875	-0.672607628	-1.096084063
ACH-000893	NCIH1651	NSCLC													
ACH-000448	NCIH1666	NSCLC	-2.39205269	-2.138674593	-1.674592911	-1.961351098	-1.999383045	-1.942008949	-1.856307926	-1.107312764	-1.696527284	-0.295364442	-2.202757972	-0.720752871	-1.421125297
ACH-000021	NCIH1693	NSCLC	-2.355953308	-2.057578179	-2.111468184	-1.93340495	-1.662446304	-1.369808675	-1.431610554	-1.180429201	-2.255918431	-1.084178271	-2.393209046	-0.52447326	-1.096383023
ACH-000747	NCIH1703	NSCLC	-2.385946804	-2.030972101	-1.787110072	-2.131950225	-1.909081601	-1.465211782	-1.648169125	-1.423887892	-1.915357403	-1.431209845	-2.278866495	-0.838928509	-1.061517847
ACH-000675	NCIH1734	NSCLC													
ACH-000282	NCIH1755	NSCLC	-2.285837797	-1.917921272	-2.103148665	-2.279220492	-1.823880052	-1.711051414	-1.745167531	-0.714794874	-1.764913594	-1.529980844	-2.234043118	-1.53721626	-1.131199818
ACH-001362	NCIH1770	NSCLC													

ACH-000379	NCIH1781	NSCLC													
ACH-000496	NCIH1792	NSCLC	-2.13076962	-2.323710911	-1.990596963	-2.010644159	-1.807859632	-1.530138326	-1.394397703	-1.403490577	-2.100076312	-1.607681093	-2.362382803	-1.851498046	-1.352670974
ACH-000888	NCIH1793	NSCLC	-2.390560003	-1.934321008	-2.224354192	-1.927894077	-2.068159877	-1.750587861	-1.588699455	-1.301554388	-1.583658712	-1.75984663	-2.194823957	-1.033275902	-1.339646946
ACH-000033	NCIH1819	NSCLC													
ACH-000733	NCIH1838	NSCLC													
ACH-000894	NCIH1869	NSCLC	-2.292992269	-1.734257341	-2.173261609	-2.072357113	-1.751116164	-1.221835727	-1.563618564	-1.668558413	-1.951490008	-1.284738977	-2.435171606	-0.858322603	-1.363956891
ACH-000434	NCIH1915	NSCLC	-2.278099352	-2.020837215	-2.357490734	-1.582949718	-1.580621419	-1.424626436	-1.54499136	-1.038877597	-1.984462635	-0.881690873	-2.113358127	-1.36949762	-1.094388566
ACH-000414	NCIH1944	NSCLC	-1.996920964	-1.922459978	-2.17811228	-1.872343012	-1.739286432	-1.433181093	-1.120406679	-1.254657284	-1.56479683	-0.871242864	-2.34661402	-1.035473726	-1.012664913
ACH-000587	NCIH1975	NSCLC	-2.328836372	-2.085168331	-2.029670969	-2.161952145	-1.635649008	-1.615591633	-1.594093472	-1.060999107	-1.351190753	-1.683956133	-2.3455106	-1.331081643	-1.097079051
ACH-001137	NCIH1993	NSCLC	-2.338994097	-2.132860741	-2.410617015	-1.916112397	-0.828253599	-1.255250353	-0.720251485	-1.047807279	-0.355439292	-1.196700196	-2.530066219	-0.858998167	-0.574926303
ACH-000886	NCIH2009	NSCLC	-2.370856854	-2.071194284	-2.216161438	-1.984629084	-1.82170016	-1.512871574	-1.578345768	-0.796551491	-1.650573233	-1.364059872	-2.240168849	-0.257213578	-1.440352756
ACH-000781	NCIH2023	NSCLC	-2.129189133	-2.033462171	-2.164398651	-1.921575705	-1.723684226	-1.284505673	-1.363407545	-0.80852582	-0.916318062	-1.062359457	-2.262055407	-1.044125048	-1.252483923
ACH-000521	NCIH2030	NSCLC	-2.315396947	-2.169193541	-2.098753958	-1.77221478	-1.995906078	-1.33537645	-1.353936549	-1.308568927	-1.353908381	-0.813066498	-2.521445426	-1.061354357	-1.358392203
ACH-000153	NCIH2052	Mesothelioma	-2.233803767	-2.114837013	-1.961439013	-2.101353565	-1.810016694	-1.353861333	-1.422491006	-1.282575877	-1.385336144	-0.717477143	-2.021402205	-1.330545523	-0.643806995
ACH-000590	NCIH2073	NSCLC													
ACH-000451	NCIH2085	NSCLC													
ACH-000841	NCIH2087	NSCLC	-2.401545747	-1.966172211	-1.871604253	-1.910178228	-1.812851235	-1.532795177	-1.337618817	-1.41786989	-1.466381402	-1.194454655	-2.113111883	-1.286859664	-1.273883449
ACH-000904	NCIH2106	NSCLC													
ACH-000929	NCIH2110	NSCLC	-2.316302864	-2.042293132	-1.858715167	-1.710108261	-1.450625095	-1.523959813	-1.144604324	-1.531461143	-1.698118225	-1.343002138	-2.323859854	-0.996283084	-1.024202424
ACH-000311	NCIH2122	NSCLC	-2.127585405	-2.092539688	-1.647895593	-2.024643361	-1.733386065	-1.707070974	-0.999549823	-1.03274671	-2.045809484	-1.086043874	-2.220481653	-1.319994473	-1.027901398
ACH-000785	NCIH2126	NSCLC	-2.336589306	-2.388582362	-2.221177082	-2.312311552	-2.144075032	-1.413092603	-2.032075467	-1.421921223	-0.981616114	-0.372526108	-2.52243527	-0.331726204	-1.053859023
ACH-001363	NCIH2135	NSCLC													
ACH-000481	NCIH2170	NSCLC	-2.221742	-2.215093938	-2.182556467	-2.131400381	-1.898193529	-1.546088269	-1.498240906	-1.50066652	-0.726074228	-1.200515044	-2.386624448	-0.772511321	-1.305023893
ACH-000924	NCIH2172	NSCLC	-2.314996334	-2.121721501	-1.603046053	-1.611043306	-1.556126317	-1.670026511	-1.240026842	-1.363746039	-1.660833112	-1.077824214	-2.467000353	-0.764968358	-1.22188042
ACH-000447	NCIH2228	NSCLC													

ACH-000367	NCIH226	NSCLC	-2.465548498	-2.111382845	-1.710460378	-2.220820643	-0.95846229	-1.520055651	-0.889673223	-1.568533954	-1.785095148	-0.996819386	-2.357956318	-1.080127002	-0.563798269
ACH-000718	NCIH2291	NSCLC	-2.19074069	-2.333537423	-2.470856714	-1.810374944	-1.557161892	-0.888438847	-1.394436036	-1.066973109	-1.287785382	-1.03154183	-2.46129636	-0.630828936	-1.269492022
ACH-000900	NCIH23	NSCLC	-2.237869624	-1.97112919	-1.866062534	-1.953690903	-1.456878192	-1.588392934	-1.473947298	-1.286727746	-1.843048252	-1.490975758	-2.313549486	-0.928859153	-1.285295157
ACH-000951	NCIH2342	NSCLC													
ACH-000875	NCIH2347	NSCLC													
ACH-000121	NCIH2405	NSCLC													
ACH-000186	NCIH2444	NSCLC													
ACH-000092	NCIH2452	Mesothelioma	-2.319337916	-2.425110254	-2.424766241	-2.078761173	-1.687845513	-1.548242637	-2.032632291	-1.457413744	-1.417825952	-1.929467855	-2.115752777	-0.453211654	-1.221501259
ACH-000648	NCIH28	Mesothelioma	-1.886281098	-2.436015895	-2.267434515	-1.979373403	-1.300748921	-0.932809449	-1.313406895	-0.798726001	-1.56375591	-1.221302135	-2.333272802	-1.030468066	-0.732110221
ACH-000700	NCIH2882	NSCLC	-2.020426106	-1.454940115	-1.813950179	-1.725908202	-1.711348183	-1.25091188	-1.308652847	-1.496115817	-1.915373203	-1.261959737	-2.518386908	-1.020986311	-1.138324284
ACH-000251	NCIH2887	NSCLC	-1.718086545	-1.956490524	-1.63606057	-2.207715546	-1.355190059	-1.1400773	-1.214609052	-1.13664221	-2.409695363	-1.159451592	-2.571653253	-0.907680628	-0.725021219
ACH-001075	NCIH292	NSCLC	-2.376134438	-1.918943326	-1.872175303	-1.954516752	-1.891702631	-1.454836021	-1.441391485	-1.37108013	-1.610786373	-1.175332561	-2.196763977	-1.237287691	-1.153580941
ACH-000337	NCIH3122	NSCLC	-1.489842645	-2.128113605	-2.00752999	-2.136300413	-1.476767483	-1.094419642	-1.1834324	-1.321168099	-1.739939651	-0.512277773	-2.324423831	-1.708331963	-1.20809641
ACH-000837	NCIH322	NSCLC	-2.255190044	-2.228155965	-2.115819877	-2.086659042	-1.660242661	-1.097194133	-1.462184595	-1.241433081	-1.641805096	-0.804539518	-2.309982878	-1.344532722	-1.016790337
ACH-002172	NCIH322M	NSCLC													
ACH-000109	NCIH3255	NSCLC													
ACH-000860	NCIH358	NSCLC	-1.887587288	-2.211274318	-1.87898302	-2.086904913	-1.251076958	-1.225266685	-0.98490614	-1.304827358	-1.319554194	-0.38988612	-2.347034339	-1.397265966	-0.891727535
ACH-000638	NCIH441	NSCLC	-2.550642241	-1.508738393	-1.681669877	-2.181439529	-1.933418453	-1.722608824	-1.141592423	-1.524526963	-1.04827431	-0.912265721	-2.513749604	-1.273837628	-1.386723207
ACH-000463	NCIH460	NSCLC	-2.325591137	-1.699323669	-1.657543116	-2.075925078	-1.974398081	-1.472511239	-1.368558271	-0.968633674	-1.809807971	-1.217176208	-2.448840126	-0.943963178	-1.38369644
ACH-000395	NCIH520	NSCLC	-2.531507919	-2.159618019	-1.658953713	-2.107345486	-1.787495411	-1.293959474	-1.316127645	-0.939619288	-1.476293678	-0.663795205	-2.394091762	-0.90287007	-1.233524317
ACH-000343	NCIH522	NSCLC	-2.366755196	-2.023052687	-2.061612717	-2.132986271	-1.581003012	-1.676137358	-1.814536032	-1.746498996	-1.703386966	-1.589861818	-2.242911537	-1.124493904	-0.919025702
ACH-000628	NCIH596	NSCLC													
ACH-000378	NCIH647	NSCLC	-2.168250764	-1.972906796	-1.750062177	-1.992109909	-1.514196102	-1.141246287	-1.327494342	-1.235924198	-1.595099409	-1.456750738	-2.405277594	-1.18060494	-1.159642577
ACH-000945	NCIH650	NSCLC	-2.446183752	-2.138543358	-2.333797229	-2.164052512	-1.541846158	-1.1959962	-1.327397372	-1.465289744	-1.693691942	-1.403688866	-2.38975374	-1.07674455	-0.73817563
ACH-000853	NCIH661	NSCLC	-1.75159285	-1.749985427	-1.818230215	-2.179194901	-1.7751201	-1.322407636	-1.241070311	-1.370654917	-1.832105498	-1.200091663	-2.476362386	-1.308254643	-1.199203701

ACH-002174	NCIH720	Lung Carcinoid													
ACH-000775	NCIH727	Lung Carcinoid	-2.11759028	-2.180811234	-2.327856281	-2.075684421	-1.618355168	-1.080429859	-1.748012196	-1.493438241	-1.975692417	-0.829559583	-2.263076405	-0.90681746	-1.441064546
ACH-000789	NCIH810	NSCLC													
ACH-002177	NCIH835	Lung Carcinoid													
ACH-000416	NCIH838	NSCLC	-2.322490168	-1.82114228	-2.150178547	-1.960293038	-1.847532081	-1.576433442	-1.811609196	-1.078863794	-1.457932656	-1.203948621	-2.261958933	-1.096647865	-1.274405182
ACH-000710	NCIH854	NSCLC													
ACH-001977	NO36	Mesothelioma	-2.194007808	-1.713357598	-1.772083128	-1.794644989	-1.710513088	-1.633625389	-1.31951176	-1.424166424	-1.724602512	-1.374992531	-2.344050031	-1.068209427	-0.863359497
ACH-001992	ONE58	Mesothelioma	-2.212230868	-2.304055904	-2.04284084	-2.142454506	-1.91546194	-1.07047574	-1.624418448	-1.158436732	-1.57420184	-1.38348418	-2.299137698	-1.148518953	-1.330655012
ACH-000030	PC14	NSCLC	-2.202417637	-2.096899502	-2.101466328	-1.904768504	-1.657831815	-1.126149229	-1.334648007	-0.890961878	-1.326883692	-1.145986964	-2.370867905	-1.160408807	-1.007112173
ACH-002184	PC3JPC3	NSCLC													
ACH-000779	PC9	NSCLC													
ACH-000791	RERFLCAD1	NSCLC	-2.360400197	-2.19805286	-1.966807566	-2.036769145	-1.983383473	-1.431772651	-1.473687972	-1.183162981	-1.461452771	-1.52004414	-2.429157249	-0.952171606	-1.174047656
ACH-000774	RERFLCAD2	NSCLC	-2.490274215	-1.723490266	-2.002523828	-1.990814032	-1.535154658	-1.081297401	-1.765164573	-1.323686818	-1.25368329	-1.225861632	-2.358846396	-0.126821092	-1.246551316
ACH-000261	RERFLCAI	NSCLC	-2.369505958	-2.329838814	-2.106184995	-2.154586801	-1.872371232	-1.673203741	-0.908850266	-1.432542682	-1.8063102	-0.69260336	-2.076900135	-0.098970052	-1.65084137
ACH-000482	RERFLCKJ	NSCLC													
ACH-000062	RERFLCMS	NSCLC													
ACH-000442	RERFLCSQ1	NSCLC	-2.345823282	-2.051781585	-1.975786693	-1.939241609	-1.491843834	-1.171148309	-1.756596345	-1.249576663	-1.040372831	-1.455305862	-2.344372332	-1.453396268	-1.017694506
ACH-000309	SKLU1	NSCLC													
ACH-000665	SKMES1	NSCLC	-2.310752374	-2.113051373	-2.20868141	-2.073728088	-1.781720574	-1.377950172	-1.643719126	-1.246566774	-1.914951701	-1.719425293	-2.279581112	-0.992838043	-1.01957481
ACH-000553	SQ1	NSCLC													
ACH-000677	SW1573	NSCLC	-2.302540544	-1.886515877	-2.296511119	-1.890459051	-1.679691838	-1.193578794	-1.694739664	-1.141885577	-1.396396561	-1.787256332	-2.317514111	-1.440148377	-0.701053006
ACH-000669	SW900	NSCLC													
ACH-000813	T3M10	NSCLC													
ACH-001417	UMC11	Lung Carcinoid													
ACH-001233	VMRCLCD	NSCLC	-2.259733819	-2.072836451	-2.103445017	-2.25806125	-0.987554376	-0.869655476	-0.765169042	-1.211617005	-1.635151055	-1.253550013	-2.286439588	-1.553167322	-1.184362525

ACH-001234	VMRCLCP	NSCLC													

**Supplementary Table S5: 2416 risk prognostic genes and its distribution in CM, CN, DN**					
**CM:**CACNA2D2,PLA2G1B,GNG4					
**CN:**CYP4B1,KL,SLC24A4,EREG,HSD17B6,NME1,FGF5,WASF3,GNG4,CACNA2D2,PLA2G1B					
**DN:**PLK1,CHEK1,CDK1,PKMYT1,MCM4,ORC1,ESPL1,CDC45,PLK4,MCM6,RFC4,MCM2,ORC6 TRPA1,E2F7,KCNV1,SLC2A1,CDC25C,KIF20A,NEK2,KCNF1,KCNG1,FEN1,GRIA1,EGLN3,MCM10,PTGDS,MS4A2,MYBL2,AGMAT,INHA,ADAM12,CHRNA6,CTSG,KCNK3,CPA3,SLC7A11,GATA1,TEX15,CNGA3,PRKCE,SLC13A5,TPSAB1,CA9,CHRNA5,CRP,FFAR4,GNG4					
**Genes**	**HR**	**Low 95%CI**	**High 95%CI**	**cox p**	**log rank p**
PITX3	3.181347215	2.374159574	4.262969607	9.15E-15	3.21E-08
DKK1	1.196256129	1.129900492	1.266508632	7.53E-10	2.64E-06
LDHA	1.786551585	1.461889493	2.183315895	1.42E-08	3.87E-05
GNPNAT1	1.717013401	1.421339862	2.074194285	2.07E-08	7.39E-06
ERO1A	1.517830738	1.306835513	1.762892212	4.65E-08	0.000256438
C1QTNF6	1.493324182	1.293185709	1.724436867	4.71E-08	2.44E-07
LDLRAD3	1.493756267	1.291197903	1.72809124	6.76E-08	6.93E-06
KRT6A	1.134395921	1.083175767	1.188038123	8.83E-08	0.001759207
TLE1	1.725911003	1.410184138	2.112326119	1.19E-07	5.98E-06
RHOV	1.22901521	1.138527711	1.326694442	1.26E-07	0.000125211
GAPDH	1.615545985	1.351981858	1.930491015	1.30E-07	0.000178015
FAM83A	1.262597755	1.157518002	1.377216672	1.45E-07	0.001341257
ANLN	1.327565378	1.194413334	1.475561083	1.48E-07	1.02E-05
RGS20	1.369244717	1.215800507	1.542054874	2.19E-07	0.005924664
PKP2	1.278225995	1.164246509	1.40336405	2.59E-07	0.00011507
LINGO2	1.698844071	1.388291419	2.078865529	2.67E-07	0.033840691
GJB3	1.211557881	1.125672882	1.303995612	3.13E-07	8.69E-05
MELTF	1.318384743	1.18575984	1.46584348	3.23E-07	0.002447538
ARNTL2	1.330460254	1.192095961	1.484884226	3.47E-07	4.65E-05
VDAC1	1.890446414	1.479015988	2.416327932	3.67E-07	3.43E-06
PLEK2	1.419298794	1.239296818	1.625445201	4.18E-07	0.004789505
AHSG	1.909738444	1.486257005	2.453883084	4.24E-07	0.011790467
VEGFC	1.346047648	1.19901695	1.511108138	4.77E-07	0.028475646
GALNT2	1.807777657	1.431633728	2.282748718	6.54E-07	0.004478388
CREG2	1.3759652	1.213287224	1.560455097	6.64E-07	0.0013252
PLCD3	1.399814192	1.225526578	1.598888026	7.14E-07	0.025481102
IGFBP1	1.229813134	1.132928168	1.334983441	7.77E-07	0.001386741
FUT4	1.627831121	1.341364122	1.97547714	8.07E-07	2.54E-05
TRPA1	1.712580814	1.382784612	2.121033905	8.24E-07	0.032008234
LYPD3	1.20954078	1.121282207	1.304746378	8.60E-07	0.000160408
FKBP4	1.513879949	1.28317422	1.786064949	8.85E-07	5.48E-05
PLK1	1.358297956	1.200867418	1.53636722	1.10E-06	1.36E-05
IGF2BP1	1.21012912	1.120101926	1.30739217	1.33E-06	0.031994862

HMMR	1.374408509	1.208151295	1.563544861	1.34E-06	2.68E-05
TNS4	1.179471144	1.102903616	1.261354265	1.43E-06	3.06E-05
MESD	2.076958251	1.542673677	2.796285204	1.46E-06	0.006693453
FAM117A	0.599075625	0.485879459	0.738643294	1.63E-06	0.002436985
KYNU	1.253493225	1.142660693	1.375075975	1.72E-06	0.023835505
AVEN	1.746906282	1.387598064	2.199254697	2.05E-06	0.000600583
FOSL1	1.216190514	1.120718922	1.319795122	2.70E-06	0.000237741
KRT18	1.472617754	1.252002771	1.732107228	2.95E-06	0.026467088
SFTA3	0.853498882	0.798581558	0.912192792	3.04E-06	0.001014263
MSANTD3	1.876796069	1.44076882	2.444780478	3.06E-06	0.003718554
MGAT5B	1.360117839	1.195284461	1.547682243	3.07E-06	0.015649441
DLGAP5	1.311066009	1.170003581	1.469135743	3.11E-06	7.71E-05
EXO1	1.329914107	1.179672676	1.499290072	3.14E-06	0.000851198
E2F7	1.380701794	1.205378434	1.581526091	3.23E-06	0.000309491
BZW1	1.773097495	1.392681181	2.257426014	3.35E-06	0.001364993
ANKRD65	0.797337117	0.724636148	0.877331995	3.44E-06	3.27E-05
PLEKHB1	0.758404097	0.67477795	0.852394146	3.50E-06	0.000876426
ECT2	1.383860073	1.206220139	1.587661024	3.57E-06	8.83E-05
KCNV1	1.679725351	1.348785048	2.091865756	3.61E-06	0.003990734
CCT6A	1.465501477	1.246059512	1.72358909	3.87E-06	0.000425487
AP3S1	2.095350436	1.528472446	2.87247144	4.31E-06	0.001094795
CCNB1	1.351718995	1.188307879	1.537601722	4.55E-06	0.000404813
LOXL2	1.292949273	1.158228738	1.443339962	4.73E-06	0.001821536
SHC1	1.622166169	1.318256691	1.996138611	4.87E-06	0.00420349
NTSR1	1.264173068	1.143234837	1.397904869	4.90E-06	0.003447348
RRM2	1.30492074	1.164123272	1.462747268	4.91E-06	0.000134752
KRT8	1.466522356	1.244219397	1.728543877	4.99E-06	0.013897695
AHSA1	2.048137782	1.504328991	2.788531231	5.27E-06	0.000122596
IRX5	0.811351958	0.741480311	0.887807795	5.37E-06	1.44E-05
RHOF	1.443111693	1.231742946	1.690751603	5.64E-06	0.006351829
SEC61G	1.540049886	1.276600046	1.857867434	6.45E-06	0.001161013
ARHGAP11A	1.361757765	1.19057797	1.557549574	6.64E-06	0.000252092
RHCG	1.216703865	1.117169703	1.325106017	6.66E-06	0.041784846
FSCN1	1.278735178	1.148641961	1.423562529	7.07E-06	0.001468652
GNG7	0.702797407	0.602460534	0.819844898	7.21E-06	2.34E-05
PPP1R3G	1.432253499	1.223891946	1.676087576	7.51E-06	4.69E-05
SLC2A1	1.25488013	1.135979612	1.386225707	7.81E-06	3.51E-05
CD109	1.265272657	1.141227412	1.402800948	7.85E-06	0.005545416
B4GALT1	1.634256329	1.317495536	2.027174798	7.89E-06	1.64E-05
CCNA2	1.303967553	1.160413912	1.465280071	8.19E-06	7.17E-05
ADM	1.263695228	1.13995333	1.400869305	8.54E-06	0.00052999
CDKN3	1.305555463	1.160888083	1.468250981	8.60E-06	8.49E-05
HMGA1	1.330556306	1.17326665	1.508932418	8.61E-06	0.000110314

PRC1	1.343541996	1.179356736	1.530584461	8.97E-06	4.61E-05
CIDEC	1.285674727	1.150602617	1.436603289	9.12E-06	0.002812182
KIF14	1.333548888	1.173896711	1.514914062	9.68E-06	1.50E-05
CERS4	0.753638107	0.664768332	0.854388467	9.95E-06	0.000759042
MYLIP	0.66562668	0.555600779	0.797441066	1.01E-05	0.000729167
OIP5	1.335759674	1.174662768	1.518949912	1.01E-05	7.47E-05
ABCC2	1.145447237	1.07838721	1.216677425	1.03E-05	0.009203406
NKX2-1	0.860748924	0.805189442	0.920142107	1.06E-05	0.009486964
PKM	1.738493616	1.359076001	2.223834466	1.07E-05	0.003541932
HJURP	1.279854556	1.146200565	1.42909342	1.16E-05	2.38E-06
CDC25C	1.370739038	1.190336449	1.578482716	1.19E-05	2.75E-05
CNIH1	1.733265197	1.354935619	2.217233203	1.20E-05	0.005247428
SMS	1.585145146	1.289593009	1.948432658	1.21E-05	0.013519106
TMEM125	0.766362698	0.679929444	0.863783426	1.31E-05	1.53E-05
SH2D5	1.457986875	1.230163837	1.728002127	1.36E-05	0.000864381
DSG2	1.367573384	1.187594526	1.574827871	1.37E-05	0.005957553
ERLIN1	1.68321928	1.330368257	2.129656303	1.44E-05	0.002235663
TPI1	1.650454994	1.315071511	2.071371529	1.54E-05	0.000363086
SLC47A1	0.758395646	0.668896916	0.859869349	1.59E-05	0.00035363
MSANTD3-TMEFF1	8.43762645	3.202324968	22.2318287	1.60E-05	0.013168452
ITGA6	1.269332528	1.138900382	1.414702367	1.63E-05	0.003353831
EIF5A	1.659159278	1.317907056	2.088773634	1.64E-05	6.15E-05
KIF18A	1.436963418	1.218297403	1.694876685	1.67E-05	6.30E-05
SOWAHC	1.50337419	1.248397232	1.810428521	1.71E-05	0.01488701
TK1	1.323078421	1.164549696	1.503187468	1.71E-05	0.000104989
PLIN3	1.564914191	1.275178969	1.920480563	1.81E-05	0.000779239
CTSL	1.338542324	1.171430821	1.529493267	1.82E-05	0.001594925
SRGAP1	1.576786706	1.280130685	1.942189454	1.85E-05	0.009224431
KIF20A	1.331759526	1.168090398	1.518361454	1.85E-05	8.59E-05
CFL1	2.029247123	1.466862221	2.807246534	1.92E-05	0.000350007
ARL14	1.172487713	1.089931163	1.261297488	1.94E-05	0.004687546
PTPRH	1.21107842	1.109085058	1.322451266	1.99E-05	0.000483599
FAM207A	1.703601703	1.33338165	2.176615196	2.03E-05	0.001180479
PSMB7	1.9503875	1.434367594	2.652047785	2.04E-05	0.001684659
PTGES3	1.709431766	1.33526624	2.188445175	2.10E-05	0.00049955
TPX2	1.243410852	1.124655865	1.37470545	2.10E-05	0.00198147
YWHAG	1.64162907	1.306365653	2.062933909	2.11E-05	4.84E-05
FOXM1	1.256198444	1.130784852	1.395521463	2.13E-05	8.85E-05
KNL1	1.389929774	1.194116317	1.617853092	2.14E-05	7.40E-06
SPC25	1.340745159	1.171099072	1.534966277	2.15E-05	0.001023014
FSIP2	1.58329376	1.280618025	1.957507297	2.19E-05	0.010054915
NEK2	1.276028937	1.140199582	1.42803933	2.19E-05	0.000372676
SMOX	1.32647335	1.164080899	1.511519991	2.23E-05	0.003081004

SLC16A3	1.374850725	1.186676629	1.592864029	2.24E-05	4.21E-05
CLEC17A	0.599388598	0.472906778	0.759698757	2.31E-05	0.000363858
MYO1E	1.427565074	1.210478747	1.683583497	2.34E-05	0.006365755
LYAR	1.608480266	1.290377107	2.005002067	2.36E-05	0.006024504
KRT81	1.124977342	1.064980408	1.188354274	2.54E-05	0.002851974
PSMD2	1.612331276	1.290715239	2.014086503	2.58E-05	0.008183051
KPNA2	1.369549488	1.18294627	1.585588329	2.58E-05	0.000245734
DNER	1.194652258	1.099546578	1.297984137	2.65E-05	0.022550029
C18orf54	1.545468754	1.261154105	1.893879313	2.71E-05	0.002104064
SLC25A42	0.640947996	0.520587812	0.789135519	2.77E-05	0.000198227
NPAS2	1.35824903	1.17699267	1.56741879	2.79E-05	0.00283208
ASPM	1.297776456	1.148760189	1.466122997	2.81E-05	0.004351271
ESYT3	0.749719158	0.654862462	0.858315826	3.00E-05	0.00073327
GSTM2	0.686043612	0.574744528	0.818895727	3.02E-05	0.023281766
HNRNPC	2.238779653	1.532833419	3.269849333	3.05E-05	0.002127766
CKAP2L	1.311507988	1.154465661	1.489912831	3.08E-05	0.002395931
DEPDC1B	1.296959268	1.14763722	1.465709993	3.09E-05	1.35E-06
BCAR3	1.370467729	1.181538019	1.589607584	3.13E-05	0.002356958
YWHAZ	1.646115841	1.301838428	2.081439065	3.14E-05	0.00032697
SHCBP1	1.357706194	1.175651157	1.567953299	3.14E-05	0.000157467
RIPK2	1.489205751	1.234101391	1.797043408	3.27E-05	0.000870329
SERPINB5	1.141522242	1.072367089	1.215137095	3.31E-05	0.000131804
CYP4B1	0.8817698	0.830822481	0.935841289	3.42E-05	0.001833309
ENPP5	0.75771978	0.664444547	0.864089061	3.48E-05	0.011361493
H2AX	1.44836562	1.215273391	1.726165475	3.51E-05	5.05E-06
NEIL3	1.247463144	1.123385054	1.385245682	3.53E-05	5.70E-05
RPE	1.841858225	1.378596261	2.460794228	3.60E-05	0.000154862
INPP5J	0.722204798	0.618803756	0.842883976	3.66E-05	6.42E-05
DTL	1.352752194	1.171872782	1.561550474	3.70E-05	0.005995352
CDCP1	1.315228134	1.15452899	1.498295028	3.77E-05	0.004596015
AHNAK2	1.223351911	1.111363377	1.346625171	3.86E-05	0.002543799
TGIF1	1.605115846	1.28103095	2.011190189	3.92E-05	0.000183729
NPAS1	1.490033645	1.232070264	1.802007831	3.93E-05	0.01734307
FADD	1.774999094	1.350155518	2.333525095	3.94E-05	3.47E-05
KIF4A	1.266764438	1.13158168	1.418096608	4.01E-05	0.003664902
SFTPB	0.910996835	0.871353195	0.952444127	4.02E-05	0.000476195
ADIPOR2	1.598286954	1.277663233	1.999369727	4.04E-05	0.010025805
CDCA5	1.279773548	1.137462446	1.439889588	4.11E-05	0.001995969
ZNF540	0.595690946	0.465040326	0.763047167	4.12E-05	0.00102308
FAM72A	1.611417595	1.282101397	2.02532083	4.31E-05	0.000955026
STRAP	1.54722862	1.254917063	1.907629176	4.40E-05	0.00181154
AP1S3	1.457287519	1.216372892	1.74591766	4.42E-05	0.002787069
LAMC2	1.212706087	1.105470224	1.330344339	4.45E-05	0.00085269

ABAT	0.733269925	0.631688239	0.851186947	4.55E-05	0.000291563
KCNF1	1.249944307	1.122668353	1.391649427	4.67E-05	2.15E-05
TEAD4	1.447275374	1.210555154	1.730285483	4.97E-05	0.018185176
SKA3	1.312321348	1.150823834	1.496482145	4.98E-05	0.000823095
ZC3H12D	0.626660476	0.499987322	0.78542662	4.99E-05	0.000229268
CHEK1	1.337302575	1.162017298	1.539028876	5.02E-05	0.00124459
ANP32B	1.855984456	1.376302172	2.502850298	5.04E-05	0.006239614
DERA	1.592787374	1.271693374	1.994955443	5.07E-05	4.70E-05
HSPA4	1.821178418	1.362528443	2.434217683	5.13E-05	0.002021195
EIF2S1	1.821984553	1.362553229	2.436328829	5.19E-05	0.002650338
TRIM6	1.330656304	1.158468201	1.528437465	5.33E-05	0.005729504
UCK2	1.297114404	1.143319101	1.471597715	5.35E-05	0.000211871
CENPH	1.431649792	1.202648988	1.704255478	5.46E-05	0.00211157
NDC80	1.270833522	1.131144096	1.427773743	5.48E-05	0.001553656
PFKP	1.297856143	1.143288462	1.47332071	5.58E-05	1.29E-05
RAD23B	1.755049395	1.33489307	2.307449523	5.61E-05	0.008189638
ITGB1	1.458882649	1.213807907	1.75343938	5.70E-05	0.002030257
CLIC6	0.867736781	0.80981376	0.929802825	5.70E-05	0.00358842
TMA16	1.852186487	1.371686311	2.501005336	5.76E-05	5.65E-05
CDCA2	1.322507312	1.15397996	1.515646417	5.84E-05	0.003056274
SPDL1	1.508821659	1.234580149	1.843981374	5.85E-05	0.006843486
CKS1B	1.360363028	1.170734731	1.580706131	5.87E-05	0.000167738
TSPOAP1	0.738799914	0.63726464	0.856512785	5.99E-05	0.001708392
NAPSA	0.903504514	0.859816513	0.949412338	6.00E-05	0.007037878
PCDH7	1.219079154	1.106575673	1.343020653	6.07E-05	0.003460777
AKT1S1	1.762854329	1.335984404	2.326116514	6.13E-05	0.018601766
TRIM7	1.36152538	1.17076725	1.583364551	6.15E-05	0.022576619
CDK1	1.278421864	1.133647675	1.441684659	6.19E-05	0.000340232
ANGPTL4	1.17930032	1.087830051	1.278461873	6.24E-05	0.000294898
SMCO2	1.667283625	1.297706856	2.142112969	6.38E-05	0.003046997
OPN3	1.26422693	1.126805295	1.418408076	6.51E-05	0.009202927
EHBP1	1.498760977	1.228654183	1.828247929	6.58E-05	9.69E-05
TESMIN	1.272117596	1.130221273	1.43182863	6.65E-05	0.000200798
BMP5	0.817708252	0.740675565	0.902752592	6.70E-05	0.003705749
STK33	0.742087912	0.640633589	0.85960911	6.98E-05	0.000183894
STAP1	0.727875462	0.622369635	0.85126693	7.03E-05	0.000422355
ALDOA	1.494479075	1.225651051	1.822270461	7.16E-05	0.003087038
SLC6A17	1.395459834	1.183776328	1.644996696	7.19E-05	0.024132027
CCT4	1.687211101	1.303137444	2.184482774	7.22E-05	0.000636831
VDAC2	1.646215085	1.286477234	2.106546494	7.42E-05	0.002075736
PGM2	1.446415183	1.205034614	1.73614671	7.43E-05	0.004409206
SGO2	1.394338197	1.182796654	1.643713651	7.50E-05	0.000202398
DIAPH3	1.36249656	1.169040226	1.587966638	7.53E-05	0.00054533

BEX4	0.760283537	0.663704843	0.870915834	7.69E-05	2.59E-06
CCNB2	1.277553417	1.131468792	1.442499118	7.70E-05	0.001406678
TYMS	1.321662894	1.150917461	1.517739425	7.77E-05	0.000327372
NCAPG	1.260504822	1.123751996	1.413899518	7.78E-05	0.000133823
IL1R2	1.226540999	1.108367041	1.35731465	7.80E-05	4.48E-05
SERPINB7	1.22295968	1.106714092	1.35141532	7.83E-05	0.036636644
DNALI1	0.816354931	0.738157147	0.902836714	7.83E-05	0.000821002
PERP	1.36024204	1.167617119	1.58464481	7.85E-05	8.69E-05
H2AZ1	1.501142927	1.227013178	1.836516615	7.86E-05	0.000156133
KIF23	1.278663058	1.131764094	1.444628988	7.88E-05	2.89E-05
MEAK7	1.554030099	1.24853166	1.934279784	7.90E-05	0.005005997
GPD1L	0.747519338	0.64689315	0.863798234	7.99E-05	0.001634018
EMC6	1.583231596	1.259949398	1.989462666	8.05E-05	0.001471164
EPGN	1.281152073	1.132665861	1.449104005	8.08E-05	0.016893602
TFAP2A	1.216704066	1.103626085	1.341368064	8.11E-05	2.34E-05
AP2M1	1.739699632	1.319564135	2.293601902	8.63E-05	0.000270703
GPI	1.499635451	1.224678396	1.836324126	8.81E-05	0.020212045
TACC3	1.345579135	1.160042273	1.560790714	8.81E-05	4.78E-05
GSK3A	1.748316476	1.32222798	2.311712162	8.86E-05	0.001358224
ATP8A2	0.670257095	0.548696416	0.818748875	8.91E-05	0.011453342
PRR11	1.275250595	1.129188208	1.440206397	8.94E-05	0.000321104
KCNG1	1.440805244	1.200187572	1.729662763	8.96E-05	0.032134858
CDIN1	1.710029256	1.307282535	2.236853915	9.02E-05	0.015342086
CLEC3B	0.809533158	0.728277988	0.899854099	9.03E-05	0.034189197
DNAJB4	1.367567906	1.169080908	1.599754103	9.14E-05	0.001871319
CD200R1	0.636067923	0.507061854	0.797895561	9.15E-05	1.85E-05
CYCS	1.529855529	1.236272666	1.893156749	9.19E-05	0.000234581
CISH	0.766098593	0.670198191	0.875721634	9.43E-05	0.003182638
XRCC5	1.796522287	1.338633745	2.411034638	9.50E-05	0.008126718
CTSV	1.205961996	1.097687447	1.324916614	9.54E-05	0.000321376
FAM72B	1.436097668	1.197338212	1.722467796	9.57E-05	1.10E-05
GTSE1	1.278082773	1.12969583	1.445960525	9.75E-05	1.93E-05
METTL7A	0.743735465	0.640797943	0.863208829	9.81E-05	0.027037279
EGLN1	1.480522883	1.215208892	1.803762318	9.84E-05	0.021634321
IER5L	1.287378867	1.133596437	1.46202325	9.95E-05	2.53E-05
VANGL1	1.52386019	1.232531658	1.884048871	9.97E-05	0.002030121
GJB2	1.166104586	1.079230327	1.259971919	0.000100143	2.79E-05
PTTG1	1.309976192	1.143332482	1.500908659	0.000100464	0.001932218
MKI67	1.249340032	1.116752576	1.397669053	0.000100623	0.000698736
CENPU	1.305271413	1.141234499	1.4928864	0.000101086	1.88E-05
KIF11	1.289804028	1.13446775	1.466409628	0.000101537	0.00141031
RAD51	1.330759928	1.152168665	1.537033631	0.000101703	0.006907253
UBE2S	1.307534466	1.141831657	1.497284097	0.000105175	0.010340446

PCLAF	1.29391199	1.135842549	1.473979151	0.000106191	0.000340407
TMEM11	1.738772223	1.314014859	2.300833072	0.000108429	0.000174918
SGO1	1.321143138	1.147278614	1.52135599	0.000109566	0.000356472
WDHD1	1.342960885	1.156546777	1.559421525	0.000109969	0.001741092
PBK	1.232668661	1.108627651	1.370588246	0.000110782	0.003074542
YARS2	1.598843135	1.26028044	2.028357571	0.000110877	0.004651538
CEP55	1.258219551	1.119827325	1.413714779	0.000111727	0.004157809
MTURN	0.75074821	0.648976803	0.868479231	0.00011469	0.003583388
DDX56	1.702479509	1.299093165	2.231122877	0.000115027	0.014801345
TRIM28	1.587929622	1.254973708	2.009221761	0.000117342	0.001295877
PPIA	1.653358278	1.279877152	2.135824983	0.000118655	1.03E-05
HTATIP2	1.467390707	1.206977031	1.783990442	0.000119468	0.006124555
BTN2A2	0.652582231	0.525035519	0.811113824	0.000119716	0.0006346
LRFN4	1.298611138	1.136643403	1.483658712	0.000120868	0.000237328
FJX1	1.335733441	1.152388206	1.548248946	0.000121622	0.046030731
DEPDC1	1.265009513	1.122001524	1.426245005	0.0001227	0.000490531
GMPS	1.519189049	1.226887037	1.881131105	0.000125362	0.016354345
ZFP3	0.697281719	0.579935108	0.838372758	0.000125543	0.00603494
PAICS	1.447325675	1.198009822	1.74852624	0.000126639	0.006949998
PGAM4	2.075208752	1.428447921	3.014804602	0.000127459	0.002997408
MAD2L1	1.288686509	1.131711492	1.467434882	0.000129736	0.0002651
CKAP4	1.462914696	1.203482277	1.778272475	0.000133667	0.001703559
SPHK1	1.286397292	1.130341588	1.463998151	0.000135202	0.004076644
CDCA4	1.408638366	1.181352851	1.67965231	0.000135464	0.000313725
FANCI	1.341169951	1.153072822	1.55995077	0.000140477	0.005454221
TUBA1C	1.478283942	1.208827449	1.807804262	0.000140584	0.000353509
CCR2	0.762673026	0.663343231	0.876876582	0.000141528	0.000201192
SETDB2	0.577105438	0.434790492	0.76600269	0.000141753	0.002349437
C16orf74	1.233689222	1.106987407	1.374892872	0.000145688	0.005910901
INTS13	1.524120837	1.226077661	1.894614345	0.00014715	0.00163028
NBN	1.557262178	1.238507289	1.958055081	0.000150302	0.000856557
FBXO45	1.469858706	1.204301954	1.793972525	0.000151584	0.000399974
FAM189A2	0.797117104	0.7088297	0.896401037	0.000153068	0.019947182
ACSL3	1.446130039	1.19435179	1.750985017	0.000157004	0.002129033
SNAI3	0.657940795	0.529426842	0.817650439	0.000159603	0.000534579
PGAM1	1.605350308	1.255292148	2.053027748	0.000162154	0.036145553
BCAN	1.301712276	1.134978024	1.492940669	0.000162957	0.015102976
TNPO1	1.656219645	1.273902239	2.15327631	0.000164678	0.024242585
UPK1B	1.118674591	1.055276637	1.18588131	0.000164922	0.001741814
MCM5	1.459080512	1.198674368	1.776058618	0.000165451	0.00019533
DAAM2	0.805119849	0.719223206	0.901275105	0.000166048	0.007272896
NUP37	1.589625467	1.248707036	2.023620476	0.000167636	0.000685274
CENPE	1.308792291	1.137399402	1.506012099	0.000171465	0.001326005

B4GALT4	1.415888445	1.180939316	1.697580954	0.00017236	0.004245473
SLC44A1	1.531764028	1.226203714	1.913467568	0.000172437	1.88E-05
F2	1.22318218	1.101090387	1.358811831	0.00017341	0.001487866
SERBP1	1.791507969	1.32111108	2.429395115	0.000175497	0.004908204
HSPD1	1.471962992	1.20263896	1.801600581	0.00017711	0.004442803
MAP4K4	1.418969234	1.181608167	1.704011317	0.000179169	0.006799379
KLHDC8B	0.710116141	0.593582955	0.849527314	0.000181798	0.008092084
BIRC5	1.229536627	1.103421109	1.370066518	0.000182324	0.000339117
ZNF555	0.470795993	0.317275538	0.698600556	0.000183107	0.004762828
ALG3	1.515345988	1.218830635	1.883997166	0.000183165	5.01E-05
GPX8	1.319103943	1.140821467	1.525247608	0.000185265	0.008613988
TMPRSS2	0.8468895	0.776189981	0.924028708	0.000186641	0.0233118
SFXN1	1.570524815	1.239349715	1.990195475	0.000187045	0.00076437
PSMD11	1.562469033	1.236202217	1.974846383	0.000188214	0.005352528
FEN1	1.380582938	1.165589117	1.635232536	0.000188412	0.023557326
DAPK2	0.719087342	0.604794513	0.854978996	0.000188424	0.014243165
PKMYT1	1.28630283	1.127031136	1.468082751	0.000189117	0.000111919
EXT1	1.514249632	1.217828052	1.882820769	0.000189246	0.000164628
S100A16	1.287132684	1.127363952	1.469543659	0.000189352	9.25E-06
CNTNAP3	1.726094883	1.294601348	2.301406183	0.000199831	0.02699107
FSTL3	1.269314948	1.119332821	1.439393544	0.00020151	0.014581093
PVR	1.420265681	1.180367956	1.708920167	0.000201902	0.001535348
HOXA13	1.367254172	1.159275281	1.612545357	0.000202836	0.013055222
APOL1	1.277858498	1.122697439	1.454463404	0.000205431	0.042370303
PSMA4	1.697769814	1.283778328	2.24526484	0.000205902	0.006589036
NXNL2	1.59434188	1.246245503	2.039667163	0.000206004	0.00060235
CYP17A1	0.464857233	0.310184919	0.696656202	0.000206336	7.42E-05
CACYBP	1.591544868	1.245157477	2.034292941	0.000206556	0.015611673
CMAS	1.443086074	1.18892333	1.751582599	0.000206738	0.000160178
DCUN1D1	1.604991801	1.250071234	2.060681512	0.000206934	0.008180904
CD302	0.764054725	0.662778274	0.880806819	0.000207839	0.001570177
C16orf89	0.902616782	0.854926137	0.95296777	0.000216143	0.007708468
MDFI	1.213326236	1.095170636	1.344229388	0.000216391	4.42E-05
UBE2M	1.72883381	1.293485329	2.31070757	0.000216828	0.011460262
LPGAT1	1.486375109	1.204774428	1.833796363	0.00021708	0.015324911
AURKA	1.261915584	1.115510016	1.42753621	0.000217903	0.009456347
EIF3B	1.520161776	1.217495475	1.898070155	0.000217952	0.005342052
CALM3	1.692266839	1.279816401	2.237638971	0.000223416	0.018819118
DUT	1.715886395	1.288136386	2.285678871	0.000223649	0.004110513
BUB1B	1.250061245	1.11026045	1.407465352	0.000225573	6.69E-05
AGFG1	1.551796566	1.228555176	1.960085008	0.000226741	0.000430674
NGEF	1.215731023	1.095812483	1.348772661	0.000227109	0.003853602
GPR78	1.600031855	1.246025274	2.054614774	0.000229637	0.001196376

ADGRF4	1.176024958	1.078788616	1.282025673	0.000231125	0.00100175
BUB3	1.689804859	1.277987425	2.234325944	0.00023225	2.27E-05
CENPM	1.303106927	1.131672381	1.500511713	0.000234395	0.000605269
POLR1G	1.471722844	1.197771505	1.808331656	0.000235809	0.002624828
CDC20	1.218386635	1.096518526	1.353799282	0.000239194	0.00680559
MAPK1IP1L	1.710312468	1.284424436	2.277415983	0.00023957	0.000217029
PPM1M	0.667976447	0.538569541	0.828477104	0.000240069	2.63E-06
BEX5	0.810255447	0.724163831	0.906581993	0.000241459	0.009197382
MTHFD1	1.537394066	1.221838033	1.93444667	0.000243211	5.05E-05
MS4A1	0.839933175	0.765123445	0.922057406	0.000247419	0.00068716
TICRR	1.33551581	1.143931316	1.559186688	0.000250233	0.0080796
LAMB1	1.32113216	1.138007653	1.533724468	0.000254069	0.048937011
HMGA2	1.165911866	1.073846598	1.265870265	0.000254582	0.02562365
PA2G4	1.652440007	1.262423367	2.16294949	0.000255699	0.002438244
CCT3	1.554888934	1.226991486	1.970412691	0.000259349	0.003713488
POLR3G	1.397262698	1.167684736	1.671977879	0.000259461	0.001818647
TMEM171	1.244494276	1.106564401	1.39961669	0.000262771	0.048082597
PPP1R13B	0.741724288	0.631722569	0.870880584	0.000264354	0.000853346
DTYMK	1.464662586	1.192520526	1.798909489	0.000273938	7.33E-05
EIF3M	1.833362743	1.322610974	2.541351169	0.00027454	1.84E-05
NMI	1.524457242	1.214673112	1.913247161	0.000274878	0.000460356
SPATA6	0.712693896	0.593717732	0.855511908	0.000278449	0.005862253
GLRX2	1.644042124	1.257297601	2.149749196	0.00027988	0.00269871
NEDD1	1.592455495	1.238930576	2.04685763	0.000280406	0.000698542
CDCA3	1.270147395	1.116361864	1.445117803	0.000281624	4.83E-05
GREB1L	1.272384505	1.117044361	1.449326798	0.00028774	0.004701024
CDT1	1.261987459	1.112840393	1.431123777	0.000287758	0.011912329
TOMM40	1.427142779	1.177314404	1.729985215	0.000291766	0.001376751
B3GNT5	1.345126609	1.145656067	1.579327031	0.000294196	0.003593846
CENPF	1.227659706	1.098590133	1.371893219	0.000295726	0.004241622
NUSAP1	1.267165459	1.11464096	1.440561004	0.000296208	0.000210945
SLC34A2	0.887970568	0.832599404	0.947024134	0.000298175	0.010794808
CACNA2D2	0.876902064	0.816598624	0.941658737	0.000301968	0.00076046
DDIT4	1.250024079	1.107392738	1.411026227	0.000305968	0.003954628
TP53I3	1.346160861	1.145444585	1.582048654	0.000308193	0.000336198
LRIG1	0.752731919	0.645090199	0.878335064	0.00030905	0.000352522
INTS7	1.610066753	1.242925087	2.08565663	0.000309932	0.000816685
SKA1	1.248436008	1.106573061	1.408485821	0.000311643	0.015439162
POPDC3	1.164285653	1.0718702	1.264669063	0.000312411	0.037985649
ATP8B3	1.254915776	1.109160864	1.419824351	0.000312584	0.000591601
ACSM5	0.60739099	0.463145405	0.796561537	0.000313155	0.001385363
PFN1	1.910759075	1.343441788	2.717646776	0.000315032	0.020371258
CAPZA1	1.722922054	1.281340738	2.316683078	0.000317186	0.000266587

KIF20B	1.367136927	1.153072321	1.620942019	0.000319096	0.001462048
CDH17	1.130285284	1.057364642	1.208234862	0.000319111	0.025349077
HSF2BP	1.461172378	1.188444149	1.796487214	0.000320856	0.018230105
CD40LG	0.745363705	0.635066505	0.874817123	0.000322162	0.031118859
STRIP2	1.207781149	1.089626776	1.338747666	0.000325513	5.20E-06
UBE2D1	1.651560611	1.256142322	2.171451756	0.000326713	0.008342062
PPP2CA	1.928610389	1.347840572	2.759627592	0.000327081	0.000890888
RAC1	1.60466776	1.239623066	2.077210962	0.00032927	0.000955672
SPECC1	1.431400561	1.176908964	1.74092273	0.000329627	0.000124988
NCAPD2	1.338814316	1.141682229	1.569984824	0.000330152	0.000711076
MRPL15	1.429011176	1.175835685	1.736699242	0.000333222	0.000122324
SPOCK1	1.168531546	1.073201116	1.272329999	0.000334514	0.000429
CTSH	0.811994172	0.724647787	0.909868969	0.000334967	0.016542226
KPNA4	1.700431095	1.272033411	2.273105316	0.000337484	0.00822391
LETM2	1.405235876	1.166520075	1.692802301	0.000341728	0.030062028
IVD	0.725080599	0.608102863	0.864560763	0.000342001	0.006267226
FXR1	1.595389849	1.235283065	2.060474106	0.000345107	0.002105023
PANX1	1.518135864	1.207794486	1.908219096	0.000346181	0.000412242
PXN	1.499008241	1.200736722	1.871372522	0.000348896	0.031546153
GNMT	0.707695131	0.58548005	0.8554218	0.000351016	0.016140929
JOSD1	1.579932359	1.229318662	2.030544509	0.000353408	0.018986606
BCAR1	1.482879017	1.194464378	1.84093408	0.000356663	0.003368176
ATP8A1	0.793726328	0.69915682	0.901087517	0.000358263	0.002945604
C3orf18	0.664837489	0.531305223	0.83193025	0.000359012	0.007186058
ABCA3	0.863092175	0.795992728	0.935847873	0.00036296	0.032946003
RAB10	1.569656023	1.225076021	2.011156849	0.000363496	0.019786582
IKZF3	0.754900644	0.64678071	0.881094586	0.000363786	0.005115467
GPR1	1.340946292	1.141202171	1.57565154	0.0003639	0.01840455
STYK1	1.27586592	1.115853562	1.458823901	0.000366276	0.007018858
YKT6	1.518693434	1.206801777	1.911191872	0.000367121	0.002248485
MCM4	1.280736144	1.117740535	1.467500746	0.000367178	0.00019813
EIF3J	1.650003385	1.252607874	2.173474418	0.000368052	0.039366618
TMEM50B	0.665355135	0.531666658	0.832659805	0.000370643	0.000325967
AP2S1	1.637482012	1.248065417	2.148402883	0.000371876	0.005823364
CCT7	1.733392422	1.279792353	2.347763119	0.000379818	0.000359671
NEMP2	1.695268483	1.266950957	2.268387116	0.000381776	0.00569689
ERO1B	0.804275909	0.713131772	0.907069021	0.000386144	0.003519768
NUP62CL	1.307898142	1.127704701	1.516884295	0.000386668	4.88E-05
CDC6	1.234600359	1.098952672	1.38699153	0.000386822	0.002809028
ZNF77	0.598757955	0.451007639	0.794911346	0.000388932	0.000255857
CYREN	1.716605777	1.273531312	2.313830344	0.000389206	0.021012997
IGF2BP3	1.169973345	1.072721257	1.276042233	0.000392035	0.001097027
FRRS1	1.346587456	1.142299145	1.587410604	0.00039286	0.009827019

FAM184A	0.782942363	0.683784896	0.896478918	0.000397624	0.012845856
RACGAP1	1.291816372	1.121102467	1.488525437	0.000399066	0.000211547
RHPN2	1.268923573	1.112180492	1.447756948	0.000399368	0.002933792
ACSS1	0.79130199	0.695070899	0.900856072	0.00040291	0.000848069
AMT	0.786235873	0.688159489	0.898290088	0.000403416	0.004998893
BTG2	0.797797845	0.703942968	0.904166147	0.000403813	0.003096701
BUB1	1.242366371	1.101544606	1.40119083	0.000406868	0.001750566
RAN	1.516882249	1.203933728	1.911178086	0.000408939	0.001915145
P2RX1	0.71753906	0.596899497	0.862561126	0.000409045	0.012966598
UHRF1	1.267527522	1.11135517	1.445645877	0.000409717	0.010874158
RSRC1	1.578192783	1.225229981	2.03283669	0.000411439	0.017971522
PIMREG	1.232665494	1.097536707	1.384431347	0.000414037	0.000616404
MELK	1.213645942	1.08989167	1.35145218	0.000417728	0.002395373
FAM83D	1.236024292	1.098656655	1.390567329	0.000423079	6.73E-05
PTBP1	1.813681532	1.302391126	2.525693422	0.000425642	0.000403144
SMC2	1.378598436	1.153080207	1.648223286	0.000427007	0.001255726
IL22RA1	1.197338326	1.08312457	1.32359574	0.0004298	0.004305619
KIF2C	1.216486639	1.09075569	1.356710542	0.00043051	0.007012471
MTMR2	1.557176444	1.21690271	1.992598471	0.000430838	0.005805485
RHOC	1.448841922	1.178599797	1.781048087	0.000431347	0.001077665
HERPUD1	0.645774343	0.506211511	0.823814735	0.00043156	0.001618468
GPR37	1.181053601	1.076491837	1.295771656	0.000434197	0.003876693
CDK5R1	1.314066674	1.128568506	1.530054414	0.000435232	0.026197774
FUCA2	1.505063032	1.198161587	1.890575323	0.000441804	0.000466866
RCCD1	1.460712463	1.182413787	1.804512872	0.000441912	0.012553839
EIF6	1.655678839	1.249678606	2.193581937	0.000443422	0.00208479
XRCC6	1.725100873	1.272572429	2.338549032	0.000443479	0.032042797
ZNF493	0.699026573	0.572415533	0.853642365	0.000444605	0.006528594
PAFAH1B2	1.676563119	1.256221999	2.237553471	0.000449937	0.000433294
PARP15	0.772448666	0.66866477	0.892340929	0.000452681	0.001115348
STC1	1.197626075	1.082779209	1.324654374	0.000454526	0.001883588
COA6	1.396692313	1.158658077	1.683628202	0.000457085	0.006372339
NEDD4	1.398163407	1.159044481	1.686614228	0.000461291	0.017821209
CARHSP1	1.484819893	1.190074932	1.852564116	0.00046297	0.00356178
TCN1	1.092605051	1.039757878	1.148138255	0.00046299	0.000855309
ELAPOR1	0.877087893	0.814977038	0.943932326	0.000465732	0.000804169
GRIA1	0.618408175	0.472463562	0.80943527	0.000466471	0.047333211
SMARCAL1	1.973399976	1.348429024	2.888032962	0.000467835	0.001073295
CENPA	1.210715444	1.087673522	1.347676354	0.000470644	0.000892672
PMEPA1	1.262566286	1.107886661	1.43884179	0.000471491	0.001346655
ZIC5	1.390734046	1.155966177	1.673181469	0.00047167	0.006029617
HTR1D	1.197044926	1.08222219	1.324050244	0.000472701	0.012256431
SDCBP2	1.172976366	1.072549333	1.28280678	0.000476495	0.000907623

FAM131C	1.379946437	1.151839773	1.653226614	0.000476973	0.003953832
RAET1L	1.30492365	1.123718714	1.515348736	0.000484534	0.000399003
CDH26	0.811738003	0.721970794	0.912666538	0.000486094	0.000388439
CCT8	1.644120329	1.243277335	2.174198451	0.000488166	0.001539977
FCRL1	0.718045739	0.596039581	0.865025915	0.000490314	0.000771872
ESCO2	1.371002879	1.148057214	1.637243222	0.000492508	0.001500254
TUBA1B	1.410379192	1.162215322	1.711532646	0.000496947	0.002363938
SMNDC1	1.816442697	1.298056678	2.541849003	0.000498401	0.004556899
MARVELD1	1.373137734	1.148484046	1.641735681	0.000503723	0.001869984
FUT1	0.709251024	0.584419277	0.860746788	0.000504894	0.035404378
GRWD1	1.732443086	1.270752351	2.361875659	0.000510362	0.013440934
NCAPH	1.225608025	1.092460574	1.374983287	0.000526154	0.005650987
SEMA4B	1.297478481	1.119821061	1.503320903	0.000527765	0.00027506
ZNF185	1.341384479	1.136119203	1.583735505	0.000528279	0.015906082
CBFA2T3	0.737725028	0.621111525	0.876232682	0.000530229	0.010837278
WDR4	1.518053411	1.19874859	1.9224099	0.000531206	0.000559453
DARS1	1.691115745	1.256149482	2.276697561	0.000533768	0.000474955
CDC25A	1.291296696	1.117256704	1.492447664	0.000538067	0.028298265
PLOD2	1.208764785	1.085688226	1.345793636	0.000539141	0.013795819
GGT6	0.87168062	0.806434323	0.942205807	0.000540796	0.0007548
SNX30	0.788629522	0.689355896	0.902199469	0.000541586	0.011964501
TPM3	1.6871269	1.254207128	2.269479349	0.000546002	0.00052705
LRRC66	1.240064591	1.097599035	1.401021813	0.000549122	0.003664324
CENPN	1.351343064	1.139159087	1.603049213	0.0005503	0.031989991
SATB2	1.375737506	1.147952295	1.648721548	0.000552342	0.007956712
RAB35	1.762126419	1.277359266	2.430866239	0.000557977	0.00338781
GPRIN1	1.268404472	1.108174661	1.451801743	0.000559152	0.003938753
BZW2	1.436946783	1.169472629	1.765595882	0.000561204	0.000239323
CRTAC1	0.871562656	0.806077982	0.942367215	0.000561616	0.003983028
BLM	1.325641981	1.129420586	1.555954163	0.0005626	0.005891117
PPP2R1A	1.824021305	1.296108587	2.566956005	0.000565239	0.000880472
LMNB2	1.340995481	1.134885125	1.584538242	0.000568872	0.000334817
TFDP1	1.372536743	1.146227542	1.643528046	0.000572141	0.015352731
VEGFD	0.840683387	0.761628896	0.927943465	0.000572792	0.018999653
CFTR	0.84258181	0.764306425	0.928873661	0.000575033	0.011162192
ZNF44	0.630384366	0.484761223	0.819752964	0.000575354	0.033785692
HASPIN	1.336872448	1.133159895	1.577207198	0.000577418	0.0101446
TMOD3	1.482659847	1.184692754	1.855569907	0.000580478	0.042164283
DERL1	1.712382363	1.260376892	2.326489304	0.000581973	0.005934172
SHISA2	0.857834755	0.786063591	0.936158952	0.000582146	0.00112158
CENPK	1.294399386	1.117403568	1.499431197	0.000582473	0.000175185
KNSTRN	1.328784079	1.129949591	1.562606989	0.000587659	6.59E-05
SEPTIN1	0.739510021	0.622570781	0.87841429	0.000590208	0.033308757

DARS2	1.414617785	1.160607007	1.724221433	0.000592668	0.003452802
ZNF441	0.631407146	0.485666542	0.820882127	0.000594576	0.012416929
MRPL51	1.606043914	1.225482256	2.104785312	0.000595632	0.0059431
IRX2	0.885771799	0.826516191	0.949275633	0.000595822	0.001826634
MMADHC	1.659183187	1.242640103	2.215354907	0.000597374	0.003077278
C11orf24	1.518486083	1.195997033	1.92793119	0.000605003	0.000151828
ANXA2	1.393695989	1.152834546	1.684880555	0.000605514	0.001682132
PXMP4	0.810297108	0.718484455	0.913842184	0.000607186	0.002152244
SPAG5	1.230727533	1.092831239	1.386023941	0.000616823	0.000436879
ZWILCH	1.43284149	1.166162862	1.760504302	0.000619513	0.022246203
ZNF254	0.723716643	0.60137755	0.870943351	0.000620524	0.016994427
GNAI3	1.715287848	1.259317083	2.336355507	0.000620718	0.015350919
C11orf16	0.800597288	0.704856253	0.909342884	0.00062072	0.001161241
MZT2A	1.446540068	1.170823884	1.787184389	0.000622389	0.004954028
PRKCD	0.690767187	0.558783128	0.853925759	0.000627169	0.000599711
FAAH	0.756231913	0.644199243	0.887748182	0.000636845	0.011036071
FAIM2	0.772152287	0.66565513	0.895687763	0.000638226	0.004406041
MTHFS	1.523806244	1.196524403	1.940608535	0.000639321	0.038316408
ZNF429	0.676514791	0.540481121	0.846786771	0.00064509	0.036949036
CCDC34	1.315605231	1.123741799	1.5402267	0.00064836	0.040759055
UBAC2	1.7166543	1.258330906	2.34191338	0.000649595	0.048494448
SNRPE	1.566183992	1.209481925	2.028085123	0.000668232	0.028677416
EGLN3	1.161870171	1.065625921	1.266806923	0.000672092	0.00012998
TTLL12	1.375512595	1.144560212	1.65306716	0.000674556	0.003381876
TMEM250	1.651098032	1.23657705	2.204573271	0.000674816	0.011080199
METTL5	1.63077842	1.229934385	2.162260268	0.000679063	0.000206263
PSMB5	1.537108868	1.199476755	1.969778624	0.000680504	0.005424399
DPYSL2	0.782920498	0.679820897	0.901655876	0.000681501	0.012421156
RAB5IF	1.435804657	1.165073684	1.769446037	0.000690934	0.006322092
KRT16	1.119236064	1.048707575	1.19450779	0.000693655	0.000181579
SH3GL1	1.602981562	1.220371474	2.10554732	0.000695643	0.013955172
MRPS35	1.449371081	1.16929509	1.796532415	0.000705236	0.029195822
ZWINT	1.248803777	1.098152732	1.420122017	0.000705507	0.004227498
STK24	1.462845094	1.173346917	1.823770736	0.00072283	0.027993205
RPP25	1.328077093	1.126513472	1.565705879	0.00072902	0.006680898
IL16	0.747943348	0.631939771	0.885241407	0.000731356	0.000192309
MIF	1.34252701	1.131575068	1.592805307	0.000732194	0.002206985
TCTA	0.710838703	0.583074435	0.866598897	0.000734595	0.028922748
JCHAIN	0.86992117	0.802319165	0.943219202	0.000734786	0.005979404
TFG	1.530183773	1.195302833	1.958886331	0.000736427	0.008516734
BEND5	0.775851842	0.669533512	0.899052952	0.000737833	0.008413173
ERCC6L	1.306950449	1.118725079	1.526844716	0.00074092	0.000938758
SLC46A3	0.756124307	0.642780353	0.88945464	0.000741504	0.019446955

DSC1	2.578853578	1.486603752	4.473610246	0.000749729	0.007515797
HLA-DMA	0.810940216	0.717895591	0.916044117	0.000751025	0.000806826
TRIM59	1.421301139	1.158443864	1.743802175	0.000752732	0.011701905
ETHE1	1.391235841	1.14810776	1.685849738	0.000753603	0.018123186
GTF3C6	1.646300795	1.231649378	2.200550218	0.000759168	0.002937982
RGS13	0.563555239	0.403611327	0.786882048	0.000759319	0.004489042
FBP1	0.803586163	0.707537684	0.912673255	0.000760129	0.002055214
LY6K	1.131005	1.052767071	1.215057297	0.000762894	0.002711895
PSMA5	1.620262099	1.22330771	2.146025278	0.000763556	0.002062697
ASPH	1.226966902	1.089180046	1.3821845	0.000763987	0.027384487
IRX3	0.849252356	0.772163176	0.934037761	0.000764245	0.002719533
TMPO	1.361931599	1.137628031	1.630460598	0.000767287	0.006340506
SMIM15	1.624832413	1.224537035	2.15598246	0.000769242	5.50E-05
GOLM1	1.298062727	1.114970075	1.511221584	0.0007715	0.001246161
UBE2C	1.168152151	1.066966583	1.278933634	0.000773343	0.004573215
LY9	0.68243343	0.546062525	0.852860919	0.000781536	0.001015427
ASB2	0.691366289	0.557419674	0.857499955	0.000781868	0.031202195
TREML1	0.677614509	0.539929931	0.850409276	0.000784599	0.006236135
ORC1	1.253423266	1.098597677	1.430068455	0.000785479	0.011831683
ZNF253	0.690636747	0.556426834	0.857218032	0.000786762	0.045502063
HSPA9	1.515049482	1.188731877	1.930944208	0.000788096	0.002268539
NUDCD1	1.43734169	1.162744855	1.776788025	0.000796823	0.002274722
SNW1	1.796833466	1.275649139	2.530954952	0.000799829	0.004941037
HLF	0.845380534	0.766281158	0.932644942	0.000804686	0.003054298
ELF5	0.84621768	0.767483258	0.933029293	0.000804798	0.012399469
MYOZ1	0.78854785	0.686219249	0.906135631	0.000808598	0.001782376
ZNF697	1.3645708	1.137632442	1.636779507	0.000809698	0.002683258
LMNB1	1.273261676	1.105268772	1.466788293	0.000818713	0.041576423
MFSD12	1.333085456	1.126260433	1.577891561	0.000831005	0.021957381
SFTPD	0.907943242	0.857939143	0.960861779	0.000833865	0.006965928
CRYBG2	1.199148286	1.077964277	1.33395572	0.000834454	0.004189617
C17orf58	1.451389698	1.166417334	1.805984869	0.000837007	0.010091344
P4HA1	1.366542444	1.137708447	1.641403171	0.000838592	0.003182542
ESPL1	1.227010889	1.088130792	1.383616503	0.000843535	0.000266436
MBIP	0.799421933	0.700918956	0.911767931	0.000847671	0.005762917
FARSB	1.585217015	1.209209047	2.078146032	0.000852657	0.000731607
CLTB	1.383541821	1.14321881	1.674384601	0.000853292	0.043470788
RTL5	0.779395123	0.673180958	0.902367708	0.000854983	0.004794042
CAPNS1	1.571866102	1.204874735	2.050638935	0.000856646	0.001283667
TCP1	1.49993275	1.181692666	1.903877649	0.000861994	0.001615309
PPID	1.682806908	1.238837404	2.285884394	0.000866995	0.001431829
POLD2	1.461309639	1.168925008	1.826828792	0.0008676	6.27E-05
DSCC1	1.264726133	1.101448881	1.452207379	0.000868335	0.002166171

MND1	1.272290166	1.1041452	1.466041121	0.000868947	0.008611922
DYNLL1	1.753880105	1.259963091	2.441417088	0.000870571	3.39E-05
KCMF1	1.670490606	1.23489704	2.259734031	0.000872604	0.000196703
WBP11	1.5452556	1.195860629	1.996733408	0.0008758	0.01517365
OLA1	1.569081507	1.203386656	2.045906661	0.000876399	0.005238307
ADA	1.298618659	1.113348789	1.514718872	0.000877456	0.000154371
DNAAF5	1.489273774	1.17769472	1.883286336	0.000882093	0.037135035
TLR10	0.774438435	0.666121239	0.900368963	0.000883463	0.001164532
ATIC	1.542471147	1.194743125	1.991404838	0.000883763	0.019426239
PSMC6	1.576571221	1.205515351	2.061837549	0.000883826	9.80E-06
NT5C3A	1.434751591	1.159747128	1.774966351	0.000884112	0.000783474
DENR	1.573126379	1.204061072	2.055316513	0.000896088	0.006764642
PSMC4	1.532017916	1.19088481	1.970869789	0.000902474	0.004052546
SLC35G2	1.273127122	1.103844582	1.468370363	0.000909327	0.044399507
ELOVL6	1.21100499	1.081242077	1.356341116	0.000930598	0.007172351
SBK3	1.349916326	1.130209716	1.612332705	0.000931342	0.032249528
LGR4	1.268006451	1.101634714	1.459504081	0.000936961	0.003317571
RGN	0.802648132	0.704645199	0.914281436	0.000937028	0.000669365
PKIB	1.180712084	1.070031982	1.302840522	0.000940295	0.042947978
TWF1	1.412314415	1.150872066	1.733148335	0.000948573	0.001679491
SLF1	1.329581507	1.122907028	1.574295056	0.000950449	0.023196688
ERF	1.488481861	1.175651042	1.88455432	0.000952283	0.014052821
TRMT10A	1.544161241	1.193282321	1.998214418	0.000954683	0.000985149
HROB	1.246638249	1.093765108	1.420878132	0.000957566	0.00044721
B3GNT8	0.840328316	0.757876936	0.931749794	0.000961391	0.049498295
RAB44	0.47623774	0.306470197	0.740047115	0.000971965	0.049612688
OSGIN2	1.350644321	1.129694025	1.614809003	0.000973928	4.57E-05
NPC2	0.816482919	0.723764475	0.921079136	0.00097836	0.000297499
KRT6C	1.272128263	1.102481869	1.467879302	0.000980761	5.97E-06
MED6	1.820207131	1.274623388	2.599319948	0.000985062	0.021953892
SLAMF1	0.744403629	0.624514043	0.887308732	0.00098636	0.000552532
ALG8	1.493731535	1.176203275	1.896979839	0.000998508	0.003323342
USP5	1.539883347	1.190720598	1.991433361	0.001000316	0.002224018
CD1D	0.742275074	0.621518888	0.886493229	0.001002129	0.000412884
PDLIM7	1.339098009	1.125203332	1.593652834	0.001007304	0.037851734
ZNF506	0.690428595	0.553544557	0.861162193	0.001017093	0.00112188
CLCN1	1.546980727	1.192452281	2.006914163	0.001018723	0.004753778
ADGRF5	0.86063872	0.786916425	0.941267692	0.001020977	0.007375694
LIFR	0.798490854	0.698147473	0.913256394	0.001022491	0.019746905
SLC4A8	0.738741552	0.616562804	0.885131371	0.001027807	0.023892961
RDX	1.354032369	1.12984317	1.622706322	0.001031328	0.006169802
EFHD2	1.366986784	1.134094003	1.647705447	0.001036479	0.036832116
RMDN2	0.730884913	0.605965464	0.881556438	0.001044627	0.002617204

DHFR	1.424483841	1.152865855	1.760095682	0.001046072	0.020590929
DMWD	1.493999463	1.175057946	1.899510064	0.001050645	0.02362925
MFSD2B	1.734978645	1.247736812	2.41248865	0.001053427	0.006030156
DNAJC9	1.485185352	1.172154234	1.881813387	0.001055625	1.54E-05
CDCA8	1.22544564	1.085061714	1.383992262	0.001056377	0.003858938
SCNN1B	0.870498064	0.801155413	0.945842551	0.001058097	0.000492613
MCM10	1.220872864	1.083295789	1.375922039	0.001069525	0.007073871
C12orf76	0.654363401	0.507544918	0.843652346	0.001069922	0.017067996
ZNF574	1.583152992	1.202089893	2.085013284	0.001075105	0.008215696
HOXD8	1.221083503	1.0833078	1.376381597	0.001075543	0.049208144
UBALD2	1.491252948	1.173548516	1.894966697	0.00107877	0.00076603
COPS8	1.760229395	1.254120775	2.470581449	0.001078963	0.005327076
ACTG1	1.559142123	1.194536029	2.035036284	0.001083576	0.017433649
SLC18A2	0.731239352	0.606074189	0.882253361	0.00108376	0.009726219
XCR1	0.681967573	0.542068345	0.857972569	0.001084414	0.002439184
MZT1	1.39591403	1.142774355	1.7051275	0.001085991	0.001464464
FAM111B	1.234944426	1.08807186	1.401642477	0.001088765	0.00361896
PTDSS1	1.582258364	1.201448193	2.083769858	0.001089069	0.011010356
MTFR2	1.265729832	1.098782534	1.458042841	0.0010935	0.000352139
PPP1R13L	1.297753357	1.109786632	1.517556373	0.001095375	0.000617794
ZNF396	0.614891476	0.459032753	0.823670044	0.001111812	0.008366679
RCBTB2	0.697996878	0.562301428	0.866438565	0.001115011	0.028244835
CLIC1	1.597400348	1.205167585	2.117288833	0.001121547	0.011264205
SEMA3C	1.217301618	1.081496194	1.370160373	0.001121735	0.023122849
NPTN	1.549138496	1.190481096	2.015848962	0.00112339	0.007229611
RUVBL2	1.574442013	1.198106938	2.068986976	0.001126643	0.001404031
CTBP2	1.601985632	1.206381974	2.127317898	0.001127688	0.02081947
EIF2S3	1.477771901	1.168201042	1.869378396	0.001129159	0.013592466
FCGRT	0.730146817	0.604218673	0.882320257	0.001129181	0.010266478
PPTC7	1.489659394	1.17186939	1.893628358	0.001132088	0.031500419
KIFC1	1.204779929	1.076911768	1.34783064	0.001136558	0.008034642
SPC24	1.233151367	1.086844868	1.399153033	0.001144458	0.000401682
TRAF3IP3	0.749328814	0.629718222	0.891658604	0.001144551	0.004834681
ATF7IP2	0.716888272	0.586595491	0.876121284	0.001145071	0.000709473
NRAS	1.448523659	1.158570011	1.81104359	0.00114789	0.026544144
MAGOHB	1.537515704	1.185918555	1.993353194	0.001165825	0.040181003
ZNF25	0.661041066	0.514803316	0.848819884	0.001175242	0.005592887
EMC4	1.761433684	1.25127193	2.4795958	0.001175639	0.008780206
CHAF1B	1.308694593	1.11232435	1.539732129	0.001181559	0.00486911
BRI3BP	1.381837637	1.136498268	1.680139168	0.001182873	0.030512592
SAPCD2	1.222521865	1.082723887	1.380370128	0.001183762	0.001708005
ADAM10	1.482597697	1.168544138	1.881055117	0.001185031	0.008271692
KLHDC9	0.805368667	0.706577295	0.917972732	0.001187841	0.003744347

GLI2	1.344705584	1.124080783	1.608632703	0.001198721	0.037258992
RXYLT1	1.584192793	1.199206947	2.092772071	0.001200316	4.13E-05
PPT2	1.323773207	1.117047615	1.568756317	0.001205198	0.002043248
NLRP1	0.773540676	0.662155922	0.903662049	0.001208096	0.011375594
NUDT1	1.38987004	1.138670405	1.696486287	0.001209047	0.006983391
TMPRSS11E	1.109387152	1.04178658	1.181374263	0.001211496	0.036516388
CDC45	1.204438146	1.076100573	1.348081474	0.001212874	0.004414619
CLECL1	0.721770442	0.59239772	0.879396651	0.001215638	0.002629094
TXN	1.308069523	1.111617566	1.539239691	0.001219009	0.020772032
WFDC2	0.868911782	0.797975872	0.946153526	0.001221507	0.040951936
S100A10	1.302529461	1.109690732	1.528879126	0.001224406	0.000152587
PSMD1	1.44450104	1.155767515	1.805365895	0.001227915	0.001290175
TMED7-TICAM2	2.060229626	1.329073648	3.193612421	0.001229304	0.002756076
RHOD	1.297584102	1.107965026	1.51965492	0.001229537	0.027425698
MAPK6	1.366743318	1.130764862	1.651967938	0.001234476	0.004257618
PNMA2	0.846784238	0.765515894	0.936680155	0.001235054	0.001364452
NAA50	1.491049777	1.170111119	1.900015648	0.001236575	0.047010928
BANF1	1.530894494	1.182255908	1.982344037	0.001239022	6.79E-06
DDX52	1.592918402	1.200684948	2.113284623	0.001246293	0.00479259
KRT6B	1.129041928	1.048764019	1.21546473	0.001258944	0.028346481
SRSF9	1.572226579	1.194188012	2.069939065	0.001261173	0.008096771
IRX6	0.834400863	0.747449854	0.931466903	0.001262375	0.039362568
SAMM50	1.68092864	1.225860968	2.304927855	0.001263102	0.032530792
CBX5	1.402871383	1.141885554	1.723507327	0.001267133	0.012487901
CTDNEP1	1.933548107	1.294689553	2.887648451	0.001272803	0.017805648
TTK	1.213499589	1.078729571	1.365106967	0.001274443	0.000122069
FAF2	1.555741975	1.188623383	2.036248931	0.001289797	0.019987391
PLAUR	1.242380986	1.088547192	1.417954614	0.001290991	0.00054209
NCL	1.490827604	1.168746146	1.901667827	0.00130175	0.007708435
IKZF4	0.703033939	0.567149902	0.871474575	0.001302945	0.005333057
RCN1	1.430288131	1.149884973	1.779068502	0.001307241	5.88E-06
INAFM2	0.730458352	0.603128186	0.884669986	0.001309615	0.011970193
SPATA5	1.46360316	1.160176394	1.846386653	0.001312009	0.047081484
MRPL10	1.717874155	1.234883382	2.389773525	0.001315429	0.017963063
AMPD1	0.724570553	0.595153795	0.882129107	0.001330726	0.041847678
R3HDM1	1.517051871	1.176062033	1.956909002	0.00133472	0.000428054
MBTPS2	1.510641628	1.174125517	1.943606622	0.001334737	0.031646913
MMP14	1.245306517	1.08906922	1.423957535	0.001339349	0.026547036
NCALD	0.786404918	0.679008965	0.910787231	0.001340029	0.006327729
CANT1	1.435449335	1.150900045	1.790350781	0.001342335	0.014548739
ACTL6A	1.426746525	1.148154581	1.772936919	0.001343985	0.013652412
CD79A	0.86693912	0.794453123	0.946038749	0.001349948	0.030809941
NDC1	1.371687554	1.130632004	1.664137173	0.001350232	0.000442979

MROH8	0.518695731	0.347146532	0.775019299	0.001355762	0.026088783
MRPS24	1.394792401	1.137880549	1.709710078	0.001357529	0.002846567
UNG	1.401108424	1.139809658	1.722309335	0.001361921	0.044491594
FCRLA	0.816642918	0.721419107	0.924435808	0.001364467	0.002822105
TBC1D31	1.399976407	1.139333129	1.720246599	0.001369627	0.000800063
BLOC1S4	1.504686028	1.171457889	1.932702887	0.00137928	0.003996345
ASF1B	1.251401853	1.090724615	1.435748838	0.001381256	0.003376791
PLK4	1.271088572	1.097291628	1.472412726	0.001385606	0.003796314
GPR153	1.245611055	1.08871464	1.425118064	0.001386779	0.028968371
INPP5B	0.708811176	0.573882859	0.875463128	0.001400958	0.028385186
RHNO1	1.531192218	1.178693673	1.989108503	0.001415129	0.000299489
TBRG4	1.467680378	1.159484895	1.85779539	0.001420665	0.000134319
TMEM163	0.858232044	0.781291584	0.942749489	0.001421858	0.019898903
P2RY13	0.805361043	0.705043708	0.919952058	0.001426644	0.002952846
GARS1	1.41467771	1.143028053	1.750887055	0.00142873	0.048003932
DTNBP1	0.582882388	0.418296544	0.812227314	0.001430105	0.036816541
PSMC1	1.450242099	1.153975405	1.822571033	0.001431216	0.00204933
ZDHHC11B	0.845293964	0.762317519	0.937302197	0.001431432	0.026906586
PSME3	1.546380969	1.182783257	2.021751735	0.001435408	0.008946917
ADGRE3	0.658918477	0.509800837	0.85165329	0.001439696	0.01021067
EIF4G1	1.436618107	1.149635139	1.795240521	0.001440584	0.000356396
DEDD2	1.606018999	1.200021671	2.149375373	0.001441022	0.000429859
CD19	0.823940639	0.731346927	0.928257373	0.001452805	0.000815783
ZNF43	0.762507052	0.645200376	0.901141763	0.001466377	0.007791499
CCDC85B	1.33478553	1.117209568	1.594734292	0.001468702	0.036835391
FCRL6	0.747174901	0.624339217	0.894177905	0.001469983	0.0142031
UBA2	1.45625553	1.155167	1.835821288	0.001469992	0.029830942
PLSCR1	1.333007102	1.116603995	1.591350151	0.001471477	0.010461942
CACNB1	0.792259282	0.68632559	0.914543738	0.001474034	0.009740512
MFF	1.473203812	1.160162505	1.870711614	0.001478485	0.010754477
ADGRD1	0.841778337	0.756957339	0.936103967	0.001480708	0.000208066
PTGDS	0.853509723	0.774039571	0.941139026	0.00149047	0.044313754
ICAM3	0.758648353	0.639761148	0.89962844	0.001491798	0.004145847
LRP2BP	0.692255303	0.551668785	0.868668696	0.001495445	0.003250855
DSC2	1.237589306	1.085018573	1.411613893	0.00149573	0.001976338
AURKB	1.185692761	1.067264202	1.317262699	0.00151146	0.000952883
UBE2T	1.23370555	1.083506413	1.404725774	0.00152006	0.002100765
PRDM16	0.835541883	0.747684405	0.933723152	0.001525773	0.00116387
TASL	0.777474489	0.665408275	0.908414584	0.001527053	0.000280854
SCGB3A1	0.931190034	0.891021357	0.973169579	0.00153053	0.021116395
ATP13A4	0.879381948	0.812174612	0.952150682	0.001531118	0.026725756
ADAMTS8	0.813126888	0.715432761	0.924161392	0.001536918	0.031673249
BORA	1.368305526	1.126962522	1.661332986	0.001538983	0.04663785

CHRDL1	0.864783532	0.790411342	0.946153626	0.001543678	0.001305897
RP11-302B13-5	1.871847786	1.269780433	2.759385829	0.001544349	0.046166049
NOP2	1.381650071	1.131060718	1.687758128	0.001544817	0.025304393
SAE1	1.532774586	1.176499374	1.99693938	0.001554791	0.037350494
FOSL2	1.305758808	1.106747022	1.54055627	0.001566187	0.006097732
HNRNPF	1.60925642	1.198126662	2.161462815	0.001573011	0.02786484
RANGAP1	1.443884326	1.149670249	1.813391231	0.001579499	0.002969116
ZNF266	0.708887038	0.572556014	0.877679773	0.001592641	0.049633098
DRP2	1.40705767	1.138107524	1.739564361	0.001603773	0.00025647
TSPAN32	0.704389449	0.566568711	0.875735786	0.001608257	0.036573757
MCM6	1.305596079	1.106199109	1.540935179	0.001612768	0.005784226
PCP4L1	0.899366387	0.841975786	0.960668836	0.001617975	0.013491845
CD5	0.803362108	0.701101404	0.920538275	0.00162241	0.006546531
ITGB4	1.173128153	1.062150908	1.29570069	0.0016375	0.000748439
RNASE1	0.858163187	0.780214993	0.943898876	0.001642116	0.003066214
GLS2	0.615871135	0.455444683	0.832806419	0.001642474	0.003581074
KRT7	1.252768733	1.088742891	1.441506082	0.001646985	0.022830713
RPS6KA4	1.493413179	1.163263923	1.917263039	0.001653111	0.007549734
HLA-DOB	0.816946408	0.720195217	0.926695175	0.001668178	0.001784374
ACOT7	1.370111324	1.125886753	1.667312483	0.001668191	0.013571579
SEPTIN2	1.665373996	1.211491353	2.2893028	0.001679742	0.00067734
ACTR2	1.620062282	1.198953449	2.189077314	0.001681365	0.000651004
TOR4A	1.344827445	1.117793087	1.61797463	0.001687779	0.022233088
CLSPN	1.225789435	1.07947913	1.391930329	0.001693785	0.02547266
F2RL1	1.204235095	1.072247637	1.352469443	0.001702751	0.016877371
CD1C	0.854871007	0.775096998	0.942855464	0.001705467	0.036700628
FAM72D	1.379777196	1.128363611	1.687208886	0.001708691	0.000118704
ADGRV1	0.800229708	0.696203553	0.919799365	0.001709278	0.000548491
TMX1	1.45657822	1.15136775	1.842695446	0.001719518	0.00199367
APOC4-APOC2	0.49817891	0.322133707	0.770432343	0.001733944	0.014047293
GMFB	1.497323945	1.162971863	1.927801582	0.001742332	0.007143636
HS3ST2	0.83153785	0.740791755	0.933400232	0.001754413	0.043343122
ARHGEF4	1.2132621	1.074892083	1.36944438	0.001754628	0.047344233
LOX	1.216932539	1.076076766	1.376225982	0.00175873	0.045807599
ZNF502	0.782856751	0.671533979	0.912633927	0.001759166	0.031802182
MYO6	0.752799003	0.629930011	0.899633815	0.001788145	0.037105492
MRPS12	1.384859055	1.128875053	1.698890056	0.001793381	0.004913596
CDC42BPB	1.422947034	1.140330689	1.775606218	0.001793773	0.008469197
PSMA2	1.588199048	1.187935924	2.123326825	0.001794092	0.000127993
LRRC42	1.452315022	1.149024218	1.83566098	0.00179464	0.001834268
PDE10A	1.220414843	1.076796817	1.383187957	0.001819204	0.043888246
UBE2V2	1.476298083	1.155628908	1.885948001	0.001823263	0.004069075
RND3	1.215130452	1.074956531	1.373582998	0.00183475	0.015942123

PLOD1	1.419442223	1.138689124	1.769417291	0.001839406	0.03210046
LTBR	1.388647986	1.129455196	1.707321579	0.001840292	0.007974283
MS4A2	0.803429224	0.700051787	0.922072524	0.001842803	0.020317423
FKBP3	1.403507687	1.133807979	1.737361055	0.001849421	0.001186965
SPRR1B	1.080731456	1.029153836	1.134893967	0.001859854	0.000547737
MRPL13	1.377564414	1.125862412	1.685537855	0.001861093	0.001849643
RAD23A	1.641868548	1.201027714	2.244521335	0.001881958	5.00E-05
FIBCD1	1.184357876	1.064465865	1.317753462	0.001888486	0.002949627
CD79B	0.827647899	0.734475165	0.932640173	0.001906771	0.009528175
NCAPG2	1.26871228	1.091679873	1.474453169	0.001909563	0.000800245
ZIC2	1.189028886	1.065889789	1.326393879	0.001909755	0.004507787
LIPK	1.248329962	1.085039545	1.436194378	0.001928534	1.02E-05
SNRPA1	1.45858232	1.148737743	1.852000071	0.001947997	0.02757263
CISD2	1.62527334	1.195250489	2.21000824	0.001951967	9.59E-05
RTRAF	1.596045418	1.187244819	2.145607153	0.001955848	0.001571768
ARRB1	0.770328845	0.653051815	0.908666841	0.001957896	0.014539381
NUF2	1.192536555	1.066714978	1.333199087	0.001966534	0.002491926
ST3GAL4	1.258949474	1.088112038	1.456608991	0.001968987	0.001539285
ANKRD44	0.755034285	0.631894364	0.902171002	0.001979423	0.005083862
TFAM	1.522803047	1.166459162	1.98800712	0.001988052	0.031446934
N4BP2L1	0.734675668	0.604142584	0.893412171	0.002006885	0.000698924
TMEM130	0.856872958	0.776879235	0.945103475	0.0020076	0.000188613
HMGCLL1	0.64619136	0.489821242	0.852480941	0.002007981	0.010539115
TOP2A	1.175687528	1.06090567	1.302887904	0.00201538	0.00388822
ATP2B1	1.283724138	1.095439075	1.504371809	0.002026092	0.0117812
PPM1G	1.520093068	1.16510709	1.983236525	0.002027943	0.00019311
EIF2S2	1.613562012	1.190623196	2.186739159	0.002035652	0.00097809
VSIG2	0.89575898	0.835238567	0.960664632	0.002040181	0.023602279
MVB12B	0.760467452	0.638962035	0.905078415	0.002050556	0.007721266
GAB3	0.743405963	0.615574508	0.897783158	0.002069968	0.000642174
MYBL2	1.146500056	1.050971346	1.250711908	0.002070229	0.025586249
MOCS1	0.750902856	0.625746931	0.901091274	0.00207339	0.023236246
TMX2	1.604763504	1.187448109	2.168739741	0.002083579	0.001890867
PKP3	1.344788728	1.113461536	1.6241753	0.002098802	0.015371701
BAIAP2L2	1.162316116	1.056038577	1.279289206	0.002109023	0.0423406
HPGDS	0.830315473	0.737403679	0.93493402	0.00213234	0.026633851
SPATS2	1.431746684	1.138565623	1.800421975	0.00214043	0.000996376
AGMAT	1.27482527	1.091714436	1.488648876	0.002146901	0.003280686
KLHL32	0.682599864	0.534864973	0.871140564	0.002150921	0.012087999
KLRB1	0.796517299	0.688755327	0.921139601	0.002158368	0.025635448
SUSD2	0.89874619	0.83948324	0.962192782	0.002159818	0.013439626
HCN2	1.255056063	1.085439359	1.451178004	0.002164641	0.001521536
GPRIN2	0.845944783	0.760140285	0.941434878	0.002169958	0.026889982

ANOS1	0.838703476	0.749494252	0.938530907	0.002172281	0.038779178
CDKL2	0.841307189	0.753307745	0.939586498	0.002173526	0.010297328
PSMB6	1.515204791	1.161660873	1.976347495	0.002174121	0.007292901
IRAG2	0.776610989	0.660668151	0.912901018	0.002179569	0.003776281
CNNM1	1.249705012	1.083553323	1.441334343	0.002195528	0.017697188
PWP1	1.708045499	1.212514655	2.406090034	0.002197311	0.002713287
NCBP1	1.560777534	1.173618194	2.075655033	0.002209193	0.030221903
TNNT1	1.103949862	1.036191962	1.176138537	0.002213037	0.028361165
SLC4A5	0.688171537	0.541661206	0.87431047	0.002215962	0.00395065
NMB	1.211693847	1.07143812	1.370309635	0.00221834	0.000476021
PPP1R14B	1.367213954	1.118941232	1.670573879	0.002219726	0.046936538
MAL	0.827850214	0.733460235	0.934387366	0.002223018	0.003545763
CCNE1	1.181144445	1.061612468	1.314135093	0.002226151	0.002382032
LURAP1	0.685039274	0.537554605	0.872988162	0.002227764	0.019265935
BTK	0.802520454	0.696905219	0.924141564	0.002245117	4.72E-05
TUBB6	1.262717531	1.087152015	1.466635338	0.002258369	0.018893339
WDFY4	0.800651593	0.694109868	0.923546838	0.002276122	0.003034111
KLHDC7A	0.849801537	0.765427612	0.943476093	0.00228434	0.001410699
CCL20	1.111773675	1.038604644	1.190097417	0.002284866	0.004103168
TXNDC11	0.685606214	0.537909706	0.873856476	0.002293971	0.000701436
UNC13B	0.813800626	0.712833439	0.929069012	0.002299684	0.006804513
SAMD13	1.282103955	1.092774866	1.504235321	0.0023018	0.002339982
KIAA1328	0.59619718	0.427483569	0.831496466	0.002309989	0.009109375
LLPH	1.499071016	1.155283584	1.945162161	0.002319062	0.000351556
USP14	1.502640297	1.1562313	1.952834058	0.002321568	0.049908753
DENND1C	0.72762215	0.59282177	0.893074479	0.002352146	0.007611318
MS4A4E	0.679130121	0.529261863	0.871435776	0.002352208	0.005808612
RARS1	1.532392064	1.163849245	2.017637119	0.002357911	0.002143322
CBX3	1.468912181	1.146377378	1.882192582	0.002366189	0.003034102
PFDN1	1.669846348	1.199625348	2.324381382	0.002376738	0.002032475
ACTB	1.54117135	1.165854533	2.037311741	0.002384441	0.008218513
C5orf15	1.518029923	1.159587175	1.987271761	0.002386475	0.028386638
SPIN4	1.3103954	1.100623444	1.560148581	0.002388373	0.006585178
EIF4H	1.572725455	1.174147084	2.106606055	0.002392893	0.001995253
ORMDL3	0.731790474	0.598157067	0.895278726	0.00240376	0.003692435
PGPEP1	0.712936767	0.572938083	0.887144438	0.002416996	0.027218659
PSMD14	1.487757824	1.150943198	1.923138646	0.002418253	0.008807816
AGPS	1.504400151	1.155471676	1.958697786	0.002419138	0.000669625
SERPINH1	1.357513418	1.114215583	1.653937269	0.002419632	0.005924868
HNRNPR	1.563743066	1.171381628	2.087528366	0.002420345	0.049736161
MYEOV	1.10927401	1.037272914	1.186272978	0.002455913	0.004295389
PABPC1	1.3699849	1.117482458	1.679541914	0.002456675	0.018499965
FUCA1	0.755964023	0.630758269	0.906023165	0.002460018	0.028095097

SNX7	1.340872348	1.10883248	1.621470048	0.002481627	0.018760925
NOL11	1.535696346	1.162809396	2.028159795	0.002503987	0.005624683
ANKRD2	1.394088126	1.123826654	1.729342952	0.002513593	0.022425314
GHRL	0.64900301	0.490277436	0.859115424	0.002518227	0.00071986
C11orf21	0.718990428	0.580444421	0.890605917	0.00252139	0.01790375
CPSF2	1.588997776	1.176307413	2.146474557	0.002542163	0.00854816
VWA2	0.844205689	0.756273091	0.942362294	0.002546247	0.014575875
OPA3	1.46413572	1.142975584	1.875537357	0.002547261	0.019072062
GCDH	0.617301134	0.451217311	0.844517001	0.002554735	0.003541222
KLF10	1.389659238	1.122171105	1.720907614	0.00255571	0.013410611
CHADL	0.760558721	0.636644937	0.908590542	0.002557684	0.042738981
B3GALT2	0.792161142	0.680843364	0.921679357	0.002565113	0.007164463
CLUL1	0.776817188	0.659198551	0.915422133	0.002570522	0.013086935
SMAD9	0.786000839	0.672097981	0.919207224	0.002572782	0.00094726
RASGRP2	0.76762987	0.646345058	0.911673433	0.002579384	0.003787264
TPBG	1.271120217	1.08749966	1.48574447	0.002580669	0.042662252
SNN	0.711726812	0.570486575	0.887935103	0.002586081	0.001459539
FAM98B	1.495110897	1.150892136	1.942281578	0.002589796	0.022587102
HSP90AA1	1.433862321	1.13400073	1.813015726	0.002608455	0.004974857
FCRLB	1.3919472	1.122317871	1.726353165	0.002608825	0.002273211
FANCD2	1.339844189	1.10747013	1.620975956	0.002609588	0.003161986
CASS4	0.777841847	0.660468764	0.916073509	0.002609855	0.005112017
STIP1	1.458356711	1.140689307	1.86449043	0.002611596	0.018329248
POC1A	1.286386017	1.091809111	1.515639472	0.00261516	0.000370472
POLA2	1.407819653	1.126687866	1.7590996	0.002617	0.004750848
GNA14	0.82841792	0.732809703	0.936499951	0.002625319	0.025793304
ALYREF	1.381556656	1.119233385	1.705362634	0.002625771	0.004128939
ZNF831	0.668475705	0.514160482	0.869105625	0.002633288	0.007061717
NUP50	1.545962841	1.163852503	2.053525769	0.002634395	0.030664644
BTBD9	0.797032671	0.687481104	0.924041513	0.002637362	0.023915619
NUMBL	1.329863543	1.104358469	1.601415748	0.002638237	0.00884181
PRR15	1.115994049	1.038934835	1.198768849	0.002644734	0.007296073
TESPA1	0.753304192	0.626258416	0.90612308	0.002646885	0.00051144
FDCSP	0.899180156	0.838977572	0.963702702	0.002650241	0.005831923
SIGLEC6	0.726838511	0.59030322	0.89495399	0.002652282	0.042483514
TDG	1.380639186	1.118600016	1.704062698	0.002667391	0.016322525
ATG12	1.739667865	1.212149031	2.496759229	0.002667526	0.015712758
MALSU1	1.54334979	1.162720731	2.048581838	0.00266993	0.003783456
GPR31	0.250602032	0.101526584	0.618570779	0.002682811	0.005489023
PNPLA7	0.787481068	0.673719512	0.920451942	0.002689006	0.000647767
ST6GALNAC6	0.721042308	0.5823191	0.892812909	0.002700495	0.034582889
IL11	1.235908617	1.076167315	1.419361179	0.002704184	0.007528
ITPKC	1.360385586	1.112541516	1.663442591	0.002706363	0.032201951

BRIP1	1.283841339	1.090421047	1.511570772	0.002708939	0.029315722
APCDD1L	1.203328181	1.066213409	1.358075877	0.002711616	0.037404874
CCDC86	1.398802808	1.123228527	1.741986825	0.002717301	0.001816665
SMIM17	0.572761613	0.397861675	0.82454754	0.002720197	0.018236104
VPS18	1.445321217	1.135952234	1.83894477	0.002724344	0.025286369
MGP	0.844866579	0.756681611	0.943328773	0.002724378	0.006232803
INHA	1.097739249	1.032796685	1.166765421	0.002725379	0.024164521
SLC27A4	1.434965395	1.133075548	1.81728896	0.002729681	0.034869143
ACAT2	1.345927301	1.108237593	1.634595607	0.002730252	0.004489591
WDR3	1.426501116	1.130705373	1.799677868	0.002735153	0.001869299
TRIM16	1.250957009	1.080503585	1.448300088	0.002735814	0.018050469
ACTN4	1.363161866	1.113051382	1.669473938	0.002739176	0.005169692
SELENOK	0.603502445	0.433674291	0.839835815	0.002742399	0.007498681
NBDY	1.436934879	1.133417145	1.821731616	0.002749313	0.000265767
ZBTB18	0.81629482	0.714699819	0.932331611	0.002760838	0.017723789
CHCHD4	1.557948414	1.165346338	2.082817082	0.002763531	0.016890883
LSM5	1.466753824	1.1413336	1.884958772	0.002763918	0.03428138
RAMAC	1.554936639	1.164561136	2.076170909	0.002764111	0.016239285
KIF18B	1.175258008	1.057302646	1.306372769	0.002766753	0.000242246
CYP27A1	0.821480831	0.722208769	0.934398451	0.002766794	0.002543577
MTHFD2	1.267703807	1.085290341	1.480776971	0.002766807	0.001040861
BLK	0.791186015	0.678568512	0.922493896	0.002792182	0.010137444
HHIPL2	1.112900091	1.037440714	1.193848088	0.002826325	0.011487115
P2RY8	0.789153083	0.675551385	0.921858206	0.002826983	0.001847017
PYGL	1.24206578	1.077310866	1.432016932	0.002830327	0.008178775
GPR87	1.098446528	1.032772071	1.168297254	0.002834563	0.019951268
LILRA4	0.735732696	0.601465199	0.899973266	0.002834949	0.000192218
SP6	1.175349429	1.057031307	1.306911415	0.002840106	0.000276426
ENPP1	1.230124673	1.07368697	1.409355569	0.002840686	0.001737644
KLK11	0.908352827	0.852769998	0.967558498	0.002848352	0.010483174
NMD3	1.455523234	1.137437116	1.862562646	0.002849423	0.004251557
RANBP1	1.353078069	1.109189746	1.650592487	0.002864723	0.008477049
FRZB	0.822628444	0.7235303	0.93529954	0.002870422	0.007513192
NLN	1.421585782	1.128019839	1.791551944	0.002875948	0.016261408
ADRB2	0.808513663	0.703008779	0.929852319	0.002888047	0.011545164
GAPT	0.774148814	0.654145984	0.916166116	0.002893599	0.002495922
INCENP	1.280614136	1.088234672	1.507002678	0.002900812	0.002061563
TEDC2	1.243574415	1.077325739	1.435477934	0.002908934	0.000312755
B4GALT6	1.246567341	1.078113772	1.441341514	0.002926519	0.027121408
HAVCR1	1.148587787	1.048411744	1.25833568	0.002926631	0.024203753
CCDC181	0.750514669	0.621199709	0.906749085	0.002934438	0.041214676
MTPN	1.468406511	1.139941255	1.891516489	0.002941166	0.01948211
PRPF4	1.507432335	1.150130313	1.975734594	0.002945804	0.017066797

TIPIN	1.465475521	1.138958145	1.885599143	0.002961768	0.035290652
TMEM25	0.768086523	0.645392731	0.914105287	0.002965026	0.029844116
STYX	1.530402294	1.155842636	2.026340878	0.002966182	0.002449274
DNASE1L3	0.79701196	0.686210331	0.925704606	0.002970128	0.000576488
CCDC90B	1.389275467	1.118181844	1.726093421	0.002992369	0.004246841
FAM76A	0.631138233	0.465722384	0.855306687	0.002998878	0.01403469
RBM42	1.401474218	1.12135393	1.751570071	0.003010228	0.034702544
GAR1	1.502735412	1.148177894	1.966780348	0.003013897	0.000801423
KIAA1549L	1.325930398	1.100348925	1.597758112	0.003026834	0.025138495
PELO	1.480477151	1.142125983	1.919063772	0.003038884	0.006176407
GRPEL1	1.498244644	1.146717945	1.957531949	0.003041956	0.008040753
RELB	1.364373042	1.110942502	1.67561669	0.003041981	0.025066926
RRM1	1.385769645	1.11670772	1.71965992	0.003054644	0.016518073
CEBPB	1.336145824	1.102961535	1.618629125	0.003061999	0.009949503
CD1E	0.85157636	0.765615223	0.947188972	0.003083172	0.027093111
CES4A	0.816625196	0.71408144	0.933894474	0.003087014	0.020609706
WDR76	1.286305776	1.088713268	1.5197597	0.003088012	0.009762034
CCDC137	1.40815247	1.122439459	1.766592722	0.003093954	0.004877953
FERMT1	1.204776655	1.0648757	1.363057479	0.003095943	0.038777625
ENO1	1.426603731	1.12735691	1.805282948	0.003096676	0.023123231
ZMAT1	0.814311888	0.710656627	0.933086143	0.00310706	0.041208224
CCT2	1.22922611	1.072055945	1.409438412	0.003108705	0.003243482
FCER2	0.778997471	0.660137087	0.919259152	0.00311048	0.044618164
MS4A15	0.900606042	0.840222465	0.965329157	0.003111704	0.002951533
SERPINE1	1.164932752	1.052697307	1.289134405	0.003141664	0.003473575
SULT2B1	1.176893385	1.056316863	1.311233483	0.003142722	0.009868165
DPEP2	0.77824771	0.658872309	0.919251712	0.003167637	0.00393103
G3BP1	1.568845754	1.163078508	2.11617443	0.00318446	0.011788124
LMAN1	1.422073108	1.125346203	1.797039809	0.003188287	0.043245242
EIF4A3	1.419351876	1.124456752	1.791584909	0.003207714	0.009456402
MAPRE3	0.78208487	0.664067739	0.92107583	0.003229353	0.003899542
C9orf40	1.328808169	1.099707749	1.605636727	0.003236309	0.003173391
AUNIP	1.232231608	1.072311902	1.41600101	0.003236384	0.00051555
GJB4	1.201965448	1.063393588	1.358594744	0.003245779	0.011271325
CIP2A	1.225005542	1.070086006	1.402353239	0.003261891	0.00581427
CAND1	1.337726375	1.101976177	1.623911563	0.003264328	0.014277596
SLC3A2	1.369154636	1.11054433	1.687987024	0.003265016	0.000766869
CDC27	1.546491497	1.156604283	2.067808311	0.003265712	0.003231664
AZIN2	0.740912673	0.606712527	0.90479686	0.00326911	0.038910589
SEC14L3	0.614195129	0.443844361	0.849927791	0.003270919	0.039709228
PDCD5	1.367931957	1.110108344	1.685635324	0.003279079	0.001732423
SNRPD2	1.37410359	1.11154306	1.698684239	0.003309922	0.017076898
RPS6KL1	0.732024351	0.594469647	0.901407924	0.003310202	0.004543134

ARHGAP15	0.737591263	0.602010938	0.903705956	0.0033141	8.82E-05
ZMYND15	0.754509597	0.625170448	0.910607235	0.003324703	0.017274697
ADAM12	1.171848665	1.054118819	1.302727235	0.003328784	0.021396047
PHF5A	1.444902232	1.129995264	1.847567442	0.003342367	0.000635029
TM6SF1	0.776232608	0.65537362	0.919379486	0.003352942	0.001974086
RFLNA	1.163198378	1.051414319	1.286867073	0.003362141	0.011239577
TNFRSF13B	0.748857254	0.617227536	0.908558277	0.00336516	0.002772718
RELA	1.694873836	1.191041765	2.411835926	0.003375792	0.000961433
RUBCNL	0.749035563	0.617264671	0.908936314	0.003420929	0.001723807
PRRG1	1.297386805	1.089808168	1.544503492	0.003425198	0.005954249
YWHAE	1.561167888	1.158463778	2.10385963	0.003430037	0.00316159
PAOX	0.677750667	0.522204437	0.879628617	0.00345427	0.002824869
ZNF101	0.643877747	0.479336591	0.8649007	0.003456488	0.000454822
ZNF367	1.266241472	1.080914482	1.483343495	0.003459488	0.00111264
FBXO5	1.305566101	1.091813322	1.561166923	0.003468305	0.031689521
CIITA	0.827721382	0.729153263	0.939614098	0.003469184	0.041166858
LRRC59	1.466704688	1.13424424	1.89661324	0.003495462	0.005138979
CCT5	1.326094493	1.097204237	1.602734064	0.003505296	0.001875262
DNAJC28	0.670012142	0.512052146	0.8767003	0.0035092	0.037104153
STK38L	1.362967254	1.107107323	1.677958132	0.003509886	0.03293653
HPSE2	0.684588802	0.53074852	0.883020507	0.003523554	0.004903149
CHCHD3	1.526774105	1.149003532	2.028748478	0.003526975	0.000529659
ABCA8	0.796723864	0.683914586	0.92814063	0.003530976	0.01730532
TMEM170B	0.731409029	0.592716941	0.902554206	0.003548961	0.018078987
RAB27B	1.180868341	1.055827626	1.320717516	0.003599639	0.003865131
INTS8	1.455688008	1.1305042	1.874409292	0.003603634	0.010222541
CDC73	1.537220582	1.150711164	2.053553656	0.003613745	0.025147071
SCML4	0.663887607	0.503783299	0.874873692	0.003621779	0.000685858
FAM189B	1.409867354	1.118563017	1.777035291	0.003628439	0.008357957
SELENOP	0.841710367	0.749395641	0.945396935	0.003645455	0.009713539
PRIM1	1.267917605	1.080393842	1.487989835	0.003650434	0.02543718
MRPL47	1.380209415	1.110628548	1.715225162	0.003657093	0.003006812
NUDT15	1.433071375	1.124277157	1.826679082	0.003660473	0.021295245
C6	0.814017026	0.708529453	0.935209843	0.00366192	0.038456171
ATAD2	1.229359031	1.069522325	1.413082824	0.003663445	0.015420075
CAPS	0.853603258	0.767155462	0.949792523	0.003666717	0.014840343
CHRNA6	0.455723256	0.268197449	0.774368609	0.00366911	0.005504308
KCTD3	1.295700269	1.087925521	1.54315636	0.003672574	0.005622359
TROAP	1.164628196	1.050775788	1.290816604	0.003689022	0.001423932
CERKL	0.782141667	0.662597795	0.92325328	0.003689869	0.005033487
COL22A1	1.189317733	1.057957284	1.336988451	0.003690877	0.043011759
ARL6IP1	1.440199792	1.125807177	1.842389605	0.003695881	0.017348253
ATP5MC3	1.452620433	1.128869933	1.869219881	0.003705784	0.032206334

UBC	1.616154545	1.168630436	2.235056895	0.003707816	0.014502776
LRFN3	1.359500295	1.104760224	1.67297936	0.003718972	0.005785121
MTHFD1L	1.388323562	1.112254957	1.732914113	0.003725915	0.002540894
KHDRBS2	0.822750174	0.721086993	0.938746441	0.003740204	0.010265507
KLF5	1.242674441	1.072841437	1.43939236	0.003759	0.034005348
ATP11B	1.379910402	1.109791123	1.71577577	0.003765141	0.03017607
HSPA2	1.196222549	1.059535049	1.350543701	0.003802633	0.00302203
PTGDR2	0.588336157	0.410774409	0.842650921	0.003803875	5.56E-05
ADHFE1	0.806449406	0.697051756	0.933016293	0.003826657	0.025615833
LRP10	1.380603887	1.109369193	1.718153979	0.003852337	0.024518377
PARPBP	1.257091145	1.076384291	1.468135646	0.0038577	0.009182545
TNNC2	0.892952944	0.826942434	0.964232729	0.003858595	0.022429166
GCLC	1.131762749	1.040591437	1.230922025	0.003870781	0.030671647
AP1AR	1.482738366	1.13491905	1.937154074	0.003878639	0.002781102
PTTG1IP	1.43223436	1.122221101	1.827888692	0.003895677	0.023780938
SFTPC	0.948244837	0.914628808	0.983096382	0.003905518	0.028871058
CTSG	0.827431681	0.727532312	0.941048495	0.003907769	0.001906866
DUSP26	0.698219639	0.547026509	0.891201168	0.003913315	0.000700973
IGSF10	0.775046405	0.651841882	0.921537793	0.003913524	0.0378106
CNR2	0.672887311	0.51410598	0.880708162	0.003914128	0.006977707
C1QBP	1.371312903	1.106542136	1.699437388	0.003915299	0.001570586
PEBP1	0.714056183	0.567997624	0.897673178	0.003920266	0.003378095
HLA-DMB	0.842856183	0.750401945	0.946701364	0.003927764	0.002277835
GPN1	1.485059435	1.135050791	1.942998095	0.003930154	0.019346618
TM9SF3	1.462536422	1.129481239	1.893801076	0.003933418	0.014023042
ARPC1A	1.400120281	1.113734153	1.760147874	0.003944703	0.002231799
FTSJ3	1.49982322	1.138515463	1.975791955	0.003945994	0.003228371
HLA-DRB5	0.884004431	0.812906522	0.96132066	0.003950669	0.006080805
MIS18A	1.308655286	1.089816229	1.57143802	0.003961863	0.0064382
DDX54	1.430994528	1.121398903	1.826063262	0.00396288	0.041198473
SEC14L6	0.849773256	0.760664706	0.949320483	0.00397485	0.01596994
NR3C2	0.825835869	0.72498245	0.940719163	0.003982447	0.04845403
TUBA4A	1.279139174	1.081745853	1.512552161	0.003991947	0.000279026
COL7A1	1.139943491	1.042669247	1.246292788	0.004000456	0.018803541
GDF10	0.835774268	0.73955112	0.944517028	0.004044981	0.016422487
MUSK	0.486641617	0.297723497	0.795436255	0.004067659	0.016009606
MRPL12	1.307680066	1.088966261	1.570321521	0.004070417	0.003182956
MDH2	1.442132883	1.123321431	1.851426665	0.004075372	0.003471501
DCUN1D5	1.39446036	1.11136619	1.749666053	0.004079066	0.000506076
PDIK1L	0.713904094	0.567159245	0.898617204	0.004098547	0.01858989
ZNF10	0.718917941	0.573850509	0.900657921	0.00410655	0.041595432
PTGFRN	1.297643047	1.08596818	1.55057718	0.004135078	0.012692858
GRAP2	0.742123495	0.605209841	0.910010453	0.004154004	0.011135178

OTUD6B	1.364608536	1.103240142	1.687897662	0.004161183	0.000962589
RRAS	1.355099064	1.100779492	1.668175584	0.004165296	0.04643107
HGF	0.796838897	0.6821871	0.930759653	0.004166737	0.019337683
SLC15A2	0.847636669	0.756984265	0.949145123	0.004178339	0.001806659
CD1B	0.822665399	0.719794188	0.940238711	0.004182199	0.008634177
EIF3H	1.440651892	1.122131612	1.849585066	0.00418543	0.002154937
DNTTIP2	1.488412929	1.133684528	1.954135379	0.004192503	0.003097974
GGH	1.159761196	1.04785985	1.283612529	0.004196229	0.018342618
FANCL	1.435380522	1.120715226	1.838394979	0.004201676	0.000152897
TRIP10	1.395788678	1.110857694	1.75380343	0.004204165	0.002751649
KBTBD8	0.729273425	0.587426786	0.90537194	0.004226309	0.008949751
DNAJC1	1.520995625	1.141047656	2.027459309	0.004239933	0.012607745
KCNJ14	1.365203484	1.102820142	1.690013158	0.004253607	2.07E-05
NIBAN3	0.70647175	0.556704479	0.89653012	0.004256553	0.000451558
PAK4	1.359679593	1.10137613	1.678562433	0.004260083	0.004746487
KLF15	0.84802361	0.757363001	0.949536803	0.004268947	0.001672878
DDX23	1.521096406	1.140765403	2.028229706	0.004275711	0.002431583
TAF1A	1.42579744	1.117779166	1.818694069	0.004282053	0.017911525
BLZF1	1.532893159	1.143432665	2.055006393	0.004287591	0.005984467
ANP32E	1.239554966	1.069676687	1.436412078	0.004295279	0.00935565
ACAP1	0.788010783	0.66912349	0.928021514	0.004300948	0.008198387
POLE2	1.244795907	1.071068793	1.446701521	0.004301129	0.001709342
GPC6	1.163700674	1.048634747	1.291392701	0.004318121	0.016384395
DDIAS	1.245939297	1.071273668	1.449083254	0.004325926	0.014909782
SMARCD3	0.790223595	0.672200241	0.928969214	0.004334882	0.015667891
PIK3CG	0.799505594	0.685562216	0.932386851	0.004339377	0.006656085
CLDN2	0.914915522	0.860663021	0.972587867	0.004356167	0.020441114
STEAP1	1.14945302	1.044427705	1.265039445	0.004383821	6.39E-07
KREMEN2	1.23014753	1.066674309	1.418673847	0.004410794	0.000372424
CCR4	0.825042436	0.722676031	0.94190895	0.004435543	0.039147111
B3GNT3	1.172171325	1.050673803	1.307718542	0.004436279	0.012108673
INMT-MINDY4	0.137235809	0.034923572	0.539282376	0.004450289	0.008038283
CLEC10A	0.842194056	0.748189353	0.948009784	0.004453469	0.001470731
RAD51AP1	1.203489688	1.059269218	1.367345906	0.004454068	0.001184443
FCRL3	0.779202083	0.656060916	0.925456571	0.004474195	0.000972419
CIDEB	0.576047805	0.393725983	0.842796991	0.004499249	0.018506403
ADA2	0.826613606	0.724846355	0.942668813	0.00450063	0.000583593
SAAL1	1.448319648	1.121570308	1.870261533	0.004518901	0.002762933
OGT	0.78546731	0.664869303	0.92794011	0.004520678	0.020707339
SSRP1	1.442347084	1.120112736	1.8572819	0.004522159	0.021821755
ST3GAL6	0.765584793	0.636656385	0.920622314	0.004525771	0.002668644
AC002398-9	2.821813926	1.378588166	5.775933687	0.004533545	0.010661753
TOMM22	1.486401501	1.130328413	1.954643798	0.004557322	0.039326797

TSPAN11	0.876207762	0.799684311	0.960053902	0.004593036	0.004788094
ZNF589	0.747750197	0.611578481	0.914241384	0.004594933	0.007696984
ZNF287	0.727062122	0.583211583	0.906393743	0.004601077	0.039743578
ABC7-42404400C24-1	0.625833964	0.452521555	0.865523744	0.004612351	0.011446646
GPC4	0.852633953	0.763552525	0.952108249	0.00463097	0.045898164
RIC3	0.766139008	0.63712276	0.921280821	0.004634136	0.015202186
MIEF1	1.519906965	1.137478393	2.030910825	0.00463959	0.029165962
MRPL37	1.488078008	1.130035656	1.959563086	0.004647478	0.024185554
DPY19L1	1.232800188	1.066478339	1.425060641	0.004649174	0.003860293
FNDC4	1.171906945	1.049970646	1.30800408	0.004657285	0.031638734
KARS1	1.533059127	1.14024517	2.061197318	0.004670431	0.026113726
ASAH2B	1.520234159	1.137080986	2.032495422	0.00469871	0.020742009
STK17B	0.739775826	0.600238765	0.911750966	0.004709151	0.043687411
SF3B6	1.517692573	1.136085753	2.02747965	0.004750886	0.017783394
MTBP	1.345734551	1.095017705	1.653855892	0.004759435	0.004103121
GPR55	0.582205579	0.399885251	0.847651509	0.004767227	0.006570561
CIB2	1.20138577	1.057622704	1.364690604	0.004779984	0.04656401
BRCA1	1.238375722	1.067418698	1.436713101	0.004791307	0.006849516
MAGEH1	0.759452843	0.627274962	0.919482931	0.004796378	0.02468897
CD22	0.827539638	0.725498805	0.943932433	0.004812312	0.018074633
HNRNPL	1.757665919	1.18750053	2.601589984	0.004818799	0.015359673
VTA1	1.475679398	1.125867411	1.934179518	0.004820888	0.027941753
BAK1	1.436300061	1.116623978	1.847495582	0.004821414	0.013230283
HPF1	1.495146166	1.130148512	1.978025042	0.004850859	0.004878445
CDH24	1.213363485	1.060589791	1.388143615	0.004851612	0.022124113
NFKB2	1.37115879	1.100748452	1.707998248	0.004855972	0.034376868
SAMD9	1.219913523	1.062302018	1.400909514	0.004859138	0.032187257
XXYLT1	1.337983385	1.092547896	1.638554746	0.004862217	0.012867139
ZFP2	0.694483141	0.538821501	0.895114306	0.004867118	0.017672813
HK2	1.19201809	1.054824829	1.347055063	0.004869701	0.026227395
ZNF146	1.340630352	1.093026783	1.644323605	0.004896259	0.031038347
GCSAML	0.658554194	0.492281357	0.880987306	0.004901839	0.016806679
COX6B2	1.278930498	1.077479137	1.518046302	0.004903685	0.00482446
LETM1	1.418654593	1.111851111	1.810117232	0.004912073	0.032800804
TMEM185B	1.40829031	1.109368767	1.787756837	0.004914236	0.000744846
EFCC1	0.793918822	0.675972767	0.93244451	0.004917808	0.010510125
PXK	0.699496928	0.545119608	0.897593748	0.004967234	0.002994847
EPHA2	1.185800197	1.052848074	1.33554132	0.004973503	0.022776909
BTLA	0.732331117	0.589194731	0.910240429	0.004992126	0.005630183
PARM1	0.87050852	0.790155515	0.959032835	0.005008274	0.030122518
S100A11	1.330994008	1.089977374	1.625304426	0.005027564	0.008187971
PLIN5	0.798086565	0.681710841	0.934328937	0.005036955	0.002245001

SMG9	1.482407868	1.125844958	1.951896727	0.005041653	0.046895593
NDST2	0.532690409	0.342988638	0.827313329	0.005048256	0.030292606
SNRPA	1.502375085	1.130322402	1.996891235	0.005050834	0.001750658
AGER	0.905381738	0.844590477	0.970548583	0.005063925	0.003380867
KCNK3	0.858120557	0.771028983	0.955049561	0.005074393	0.00220155
CRYL1	0.73745019	0.595929412	0.912579195	0.005087533	0.021277339
GALNT3	1.198830812	1.055978868	1.361007648	0.005088706	0.007070991
UBE2H	1.409300566	1.108518306	1.791696244	0.005093337	0.001252968
THAP10	1.391687817	1.10430378	1.753860683	0.005099608	0.014648852
SSBP1	1.514860475	1.132713048	2.025934338	0.005108227	0.008523984
ECE2	1.277249859	1.076181216	1.515885223	0.005109497	0.011521914
RFC4	1.23411869	1.065028871	1.430054136	0.005142821	0.026106485
KLK6	1.092332243	1.02678134	1.162067992	0.005158212	0.015486535
SLC39A10	1.32543714	1.087963356	1.614745204	0.00515961	0.021680098
IQGAP3	1.182385805	1.051377462	1.329718624	0.00517146	0.003062296
LRIF1	1.360647934	1.096457278	1.688495154	0.005173845	0.00428167
ORMDL2	1.511828374	1.131426256	2.020127268	0.005190695	0.045040546
CCM2	1.349735756	1.093715605	1.665685854	0.005194293	0.035673587
PEBP4	0.914109214	0.858298647	0.973548843	0.005206366	0.023267141
MFSD14B	1.582628897	1.146733234	2.184217	0.005223494	0.028952344
CENPI	1.230226332	1.063700781	1.422821957	0.005235976	0.014292811
ZPR1	1.503163053	1.129153679	2.001055486	0.005236892	0.014215596
CALU	1.303085122	1.082093307	1.569209258	0.005237218	0.042063045
RIMKLA	0.829342645	0.727128591	0.945925152	0.005297686	0.013984858
MSX2	1.185650637	1.051848541	1.336473247	0.005313946	0.048711816
COX5A	1.420920044	1.109740171	1.819357202	0.005342648	0.011938381
TCOF1	1.396897237	1.10406393	1.767399367	0.005357136	0.027191606
REXO4	1.563586253	1.141446718	2.141845023	0.005370137	0.040761569
COL6A6	0.809526526	0.697604382	0.939405217	0.005380471	0.013139607
RTCA	1.492749275	1.125766652	1.97936259	0.005388132	0.008235435
CCDC28A	0.703473876	0.549077692	0.901285011	0.005401601	0.007208262
PSTPIP2	0.765470567	0.634071229	0.924099947	0.005411682	0.03579827
DIP2B	1.362216255	1.095543448	1.693801491	0.0054211	0.014229926
WNT7A	1.151950878	1.042633218	1.27273024	0.005425203	0.025247392
CD226	0.662315184	0.495308351	0.885632965	0.00544902	0.001266155
ANK2	0.760062949	0.626356895	0.922310733	0.00544914	0.027879424
PLA2G1B	0.902088787	0.838858886	0.970084712	0.005450788	0.01118884
KL	0.774593112	0.646870053	0.927534805	0.005466258	0.008350628
PHLDB2	1.215698687	1.05920501	1.395313734	0.005468384	0.0491284
CAT	0.771406858	0.642298314	0.926467542	0.005482273	0.023859141
ZDHHC5	1.603129308	1.148990986	2.236765656	0.005482519	0.002696542
FAM72C	1.389095181	1.101530182	1.751731775	0.005485176	3.22E-05
S100P	1.071780452	1.020584762	1.125544276	0.00550513	0.023127449

JAML	0.819608864	0.712208227	0.943205463	0.005505218	0.000657646
ACP7	1.29783155	1.079607243	1.560166204	0.005512628	0.023231167
CPA3	0.892223834	0.823101558	0.967150848	0.005574731	0.028502615
OAS1	1.193273096	1.053037356	1.352184397	0.00560334	0.034067229
PINLYP	0.720875794	0.571803784	0.908811597	0.005624603	0.031275799
ROM1	0.733534716	0.588979061	0.913569285	0.005654211	0.038314054
XXbac-BPG32J3-22	2.268784932	1.269868851	4.053477697	0.00566004	7.73E-05
TMED1	1.451857797	1.114717197	1.890964873	0.005683818	0.006084073
DDX10	1.409595949	1.105140171	1.797926444	0.005688572	0.004629728
NR0B2	0.891532745	0.821840595	0.967134795	0.005698625	0.012777521
MFAP4	0.87808305	0.800738027	0.962898997	0.005716997	0.008447842
FAM13B	0.731450977	0.585851103	0.913236364	0.00575565	0.046654183
LRRC27	0.6856788	0.524527897	0.896340155	0.005770168	0.004702992
LAMC1	1.290477133	1.076721407	1.546668637	0.005779911	0.028683919
PPIAL4A	1.814129863	1.188444423	2.769222603	0.005780359	0.003059248
SEMA3A	1.138515579	1.03831496	1.248385869	0.005782287	0.020010217
RALB	1.350099871	1.090893494	1.670896079	0.0057836	0.000186874
BMP1	1.26723956	1.071029965	1.499394185	0.005789396	0.039542228
CSGALNACT1	1.263111686	1.069924989	1.49118036	0.005815049	0.018604562
MRPS2	1.460960877	1.115847451	1.912812259	0.005829835	0.044685392
UBE2F	1.584412918	1.142246897	2.197742276	0.005840722	0.008255202
DNM1L	1.385218055	1.098567634	1.746664477	0.005875307	0.012111244
BTNL9	0.817469609	0.708246277	0.943536992	0.005883362	0.035215477
NHEJ1	2.019375532	1.224551419	3.330099067	0.005892531	0.031880139
SARM1	0.69627328	0.538037624	0.901045687	0.005921208	0.02319445
PAX5	0.806584831	0.69197602	0.940175773	0.005979977	0.015546655
ASAP3	0.808034505	0.694079518	0.940698789	0.005993446	0.012138824
PNMA8C	0.510061459	0.315535701	0.824511114	0.006005829	0.024340682
ATAD3C	0.842642636	0.745734719	0.952143696	0.006020211	0.015155726
DBP	0.729377331	0.582270898	0.913649116	0.006037339	0.006925057
MAIP1	1.505550102	1.124201719	2.016258355	0.006040877	0.042138618
CSE1L	1.327144863	1.084255759	1.624444669	0.00606343	0.02873644
EFNA5	1.188682833	1.050613906	1.344896417	0.006074538	0.005972492
LRRC10B	0.865494602	0.780634359	0.959579728	0.006076752	0.030914135
MFAP3L	1.268022158	1.070136249	1.502500448	0.006088232	0.023398035
ALDH2	0.81778013	0.708157047	0.944372925	0.006155861	0.003252119
TYRO3	1.262278989	1.068409668	1.491327057	0.006186181	0.015361065
GINS2	1.196585443	1.052279759	1.360680666	0.006197506	0.045684859
RAB21	1.422406462	1.105216499	1.830627885	0.006198426	0.027873165
C1orf105	1.53161717	1.128636224	2.078482955	0.00620423	0.00548758
ATP5MK	1.40442361	1.101076776	1.791342548	0.00622821	0.009799542
PLD4	0.797646412	0.678354846	0.937915905	0.00622961	0.002609555
NCR3	0.793674064	0.672550255	0.936611822	0.006237111	0.008059221

NIFK	1.402647241	1.100507766	1.787737755	0.006262027	0.002801408
PES1	1.46617584	1.11414528	1.929435624	0.006304182	0.000128458
IL11RA	0.751856072	0.612712176	0.922598857	0.006304523	0.009313574
LCA5	0.778049104	0.649826354	0.931572573	0.006306489	0.016453602
KIT	0.891262896	0.820577007	0.968037786	0.006324509	0.009423587
PSPH	1.231898476	1.06052899	1.430959333	0.006353959	0.017140102
NLRC3	0.765373885	0.631615781	0.927458118	0.006364577	0.003404478
MASTL	1.30533068	1.077898348	1.580750342	0.00637319	0.006355603
AK4	1.157079085	1.0419156	1.284971652	0.006379571	0.022059997
REP15	1.335444748	1.084808197	1.643988938	0.006381032	0.000831613
CREBRF	0.703444466	0.546308254	0.905778219	0.006387579	0.019034253
HNRNPU	1.509469324	1.122807008	2.029286979	0.006388686	0.01573199
SNTG2	0.36054537	0.173197705	0.750546684	0.006390252	0.010273
LCN1	1.369296098	1.092350866	1.716455639	0.00640806	0.006597444
ARHGAP9	0.806914027	0.691567811	0.941498775	0.006412537	0.000954137
PLA2G4F	0.856814055	0.766612252	0.957629262	0.006473462	0.027908779
FBXO9	0.703842534	0.546596077	0.90632614	0.006481233	0.002475189
HLA-DPB1	0.862883033	0.775927258	0.959583673	0.006504347	0.000563365
ETF1	1.50264825	1.120654317	2.01485126	0.006505755	0.03064341
TTC16	0.666433868	0.49748747	0.892754344	0.006519171	0.00918425
ACAD8	0.802560097	0.684945585	0.940370626	0.006520715	0.020769681
SLC22A10	0.062843922	0.008554983	0.461644221	0.006534562	0.005970701
IER3	1.183453984	1.048101996	1.336285341	0.006565727	0.01167286
CCDC69	0.809915818	0.695671836	0.94292107	0.00657676	0.040996201
CH25H	0.881539821	0.804881288	0.965499469	0.006600325	0.004720123
FCRL2	0.811229369	0.697536399	0.943453402	0.00661716	0.001190423
ZNF75D	0.70919239	0.553381655	0.908873364	0.006630395	0.004460847
NTRK3	0.396473191	0.203291489	0.773229571	0.006635894	0.047473724
P2RY14	0.774886473	0.644560886	0.931562959	0.006637692	0.006715091
DENND1A	1.347793111	1.086484028	1.671949356	0.006641878	0.020107927
SLC2A7	1.979389322	1.208889717	3.24097561	0.006647407	0.013253056
KRT17	1.090834168	1.024444501	1.161526253	0.006651908	0.00343586
BORCS5	1.365591806	1.090384545	1.710259916	0.006656977	0.015987213
PDGFB	1.231297539	1.059407435	1.431077016	0.006683331	0.003535449
TEX30	1.293971352	1.074022545	1.558963421	0.006703596	0.001532917
PRKCB	0.802245222	0.684093174	0.94080371	0.006715268	0.000454681
LAX1	0.832262187	0.728782147	0.950435396	0.006720585	0.01257913
SLC9B2	1.304201824	1.076232996	1.580459253	0.006739677	0.049657864
USP4	0.680121664	0.514591834	0.898897819	0.006748623	0.001020875
HLA-DQA1	0.874957575	0.794348108	0.963747191	0.006753451	0.017208664
HSPBP1	1.376124594	1.092080227	1.734047418	0.006794986	0.00760787
ARHGEF39	1.264242512	1.066778808	1.498257293	0.006809868	0.001106217
ARF3	1.468180092	1.111537702	1.939252965	0.006835826	0.005363911

NOP16	1.369441361	1.09041393	1.719869481	0.00683981	0.006261603
PDCD10	1.388346242	1.094506946	1.761071773	0.00684636	0.020284245
RNF103-CHMP3	1.590761034	1.136168189	2.227241259	0.006862955	0.000403963
DHX37	1.377541815	1.092044367	1.737677982	0.006870934	0.002777516
CENPW	1.182940822	1.04723641	1.336230268	0.00688467	0.003767357
SNF8	1.540421802	1.125832742	2.107683708	0.0069157	0.00135245
SLC24A4	0.432184482	0.235111075	0.794447586	0.006917735	0.004421275
DNAJC27	0.67406174	0.506244166	0.897510055	0.006929688	0.018828683
SELENOW	0.771842721	0.6395716	0.931469104	0.006930771	0.00763315
DHDH	0.829422908	0.72412874	0.950027698	0.006932742	0.020180672
KIF15	1.206696106	1.052835424	1.383041887	0.006938197	0.03735195
VSTM5	1.348350835	1.085349372	1.675082716	0.006938597	0.002997592
SERPIND1	0.901113435	0.835495055	0.971885372	0.006950117	0.048385827
MAP3K8	0.762316111	0.625961085	0.92837377	0.006952477	0.029871808
COX16	1.572986337	1.131977862	2.185807778	0.006966269	0.016680584
AP3B1	1.480933009	1.113390692	1.969805023	0.006976774	0.008763443
ABHD4	1.321625128	1.079260761	1.618416089	0.006977479	0.027925368
EIF3D	1.525408299	1.122300902	2.073303581	0.006999463	0.03287655
STXBP5	1.384676318	1.09299764	1.754192722	0.007001374	0.011931451
RIPOR2	0.8143847	0.701451031	0.9455007	0.007023287	0.003676852
BACH1	1.353500902	1.086041955	1.686826814	0.007043322	0.00815174
SNAI1	1.203281353	1.051721678	1.376681727	0.007056969	0.026083018
HCCS	1.507847996	1.11814203	2.033378156	0.007104185	0.001334079
SEC14L4	0.854023166	0.761320883	0.958013349	0.007110575	0.014535525
PARL	1.467991956	1.109932351	1.941560116	0.007121728	0.017922431
LYPD5	1.204475461	1.051840776	1.379259265	0.007123369	0.013137571
TNFRSF19	0.852318028	0.758656319	0.957542966	0.007136323	0.000304455
MRTFA	1.44712295	1.105287249	1.894679265	0.007186761	0.008937598
MCM2	1.200071181	1.050617123	1.370785616	0.007196643	0.027883102
SLC7A11	1.119853613	1.031117786	1.216225859	0.007199263	0.04074833
C19orf33	1.145015756	1.03724942	1.263978612	0.007249966	0.001950971
WARS2	1.504155146	1.116406803	2.026575525	0.007276514	0.02259629
GADD45G	0.849240923	0.753700801	0.956891838	0.007283138	0.016683057
KLRG1	0.721528488	0.568492687	0.915760873	0.007285794	0.001110236
HOXB7	1.129095414	1.033257424	1.23382269	0.007299137	0.00423057
SLBP	1.389375957	1.092433058	1.767033261	0.007349006	0.01976077
SPATA18	0.861504516	0.772527791	0.960729232	0.00735645	0.046696271
AIMP2	1.373979414	1.089040904	1.733469719	0.007377985	0.015290129
RGL4	0.636911305	0.457846646	0.886008479	0.00739353	0.029543796
FAM83F	1.182603421	1.045952233	1.337107764	0.007426407	0.04386539
LBR	1.364755439	1.08679458	1.71380815	0.007444713	0.011912599
C1QL1	1.174516173	1.043981211	1.32137267	0.00745064	0.023958012
TAX1BP3	1.417713929	1.097844739	1.830780541	0.00746176	0.004621081

NMUR1	0.695518507	0.533014901	0.90756561	0.007488118	0.047137357
RBCK1	1.419851739	1.098137424	1.835816643	0.00749344	0.002655119
SNX9	1.371990842	1.088058375	1.730016435	0.007509849	0.003186844
IGFALS	0.826704443	0.719018897	0.950517766	0.007525005	0.000271729
RNF180	0.79494731	0.671814381	0.940648554	0.007527985	0.027196888
SYT16	1.339509845	1.080963125	1.659896238	0.007551506	0.036786656
PCBP2	1.564051424	1.126493171	2.171568297	0.007555057	0.011186764
GAL	1.106610191	1.027333138	1.192004881	0.007563205	0.012531338
IARS2	1.403949042	1.094435781	1.800994581	0.007582348	0.037714276
SDK1	0.864300662	0.776546885	0.961971065	0.007591348	0.00839235
MRPS16	1.529548117	1.119562848	2.089670486	0.007599647	0.00976502
CA4	0.845787277	0.747896086	0.956491326	0.007612899	0.003557822
CD2AP	1.320854145	1.076705597	1.620364634	0.007613779	0.011330878
UAP1	1.383499566	1.089863889	1.756247793	0.007654359	0.022723882
PPIF	1.342468863	1.081173165	1.666913966	0.007661465	0.046476296
ZNF835	0.717608194	0.562255541	0.915885184	0.00767961	0.017285905
PGM5	0.825713055	0.71726043	0.950564147	0.00768348	0.002030374
BCL9L	1.239530488	1.058455981	1.451582171	0.007697724	0.002422819
MYO19	1.282951022	1.068064599	1.541070949	0.007721755	3.09E-05
HOXA1	1.175284828	1.043581798	1.32360916	0.007734531	0.023573288
SASH3	0.831507053	0.725934041	0.95243361	0.007734589	0.005272461
WDR91	0.781851563	0.652343136	0.937071049	0.007735289	0.038396834
TFEB	0.758723901	0.619210471	0.929670903	0.007735929	0.031761922
SOD1	1.393797403	1.091600568	1.779653894	0.007747729	0.017101244
CD52	0.849728641	0.753749798	0.957928965	0.007748774	0.008324902
MYL12A	1.433327884	1.099641903	1.868270768	0.007757628	0.003914567
NDRG2	0.822466939	0.712238868	0.949754213	0.007764464	0.015975238
GATA1	0.564066017	0.370014145	0.85988732	0.007775193	0.02844244
MTCL1	1.205224254	1.050400459	1.382868304	0.007793258	0.008302513
KIRREL3	1.331595353	1.078345494	1.64432104	0.007796099	0.001705699
GPR174	0.808331641	0.691042698	0.945527741	0.007808681	0.014188985
RSPO1	0.685582052	0.519118528	0.905424725	0.007812442	0.00408808
CTD-2568A17-1	1.668928639	1.144233517	2.434225845	0.007823332	0.011092583
KCNA2	0.380303893	0.186499324	0.775504424	0.007830407	0.041159236
CTF1	0.834692586	0.730590113	0.953628717	0.007847506	0.000729714
ABCC6	0.835114328	0.731059601	0.953979595	0.007957594	0.044594196
C4orf48	1.189918701	1.046497878	1.352995113	0.007965434	0.003351847
RAB1A	1.655944459	1.140861704	2.403579717	0.007973234	0.04891821
XIRP1	1.318773533	1.074963747	1.617881195	0.007976521	0.017376624
DNASE2B	0.776565935	0.644237749	0.936074692	0.007977152	0.003806252
ABCE1	1.409903621	1.093771353	1.817407465	0.008004075	0.002089485
SYMPK	1.336084989	1.078492276	1.655202488	0.00801464	0.03732917
FGFBP1	1.090878381	1.022934386	1.163335263	0.00802398	0.001657935

PRRT3	0.795641692	0.671879439	0.942201332	0.008045713	0.027770562
BPNT2	1.351216468	1.081479797	1.688229356	0.008063013	0.00510275
SORD	1.289807085	1.06846049	1.557008734	0.008065545	0.049405145
EAF1	1.487976992	1.108855338	1.996721713	0.008082656	0.011545025
TIMELESS	1.231524871	1.055597362	1.436772735	0.008098628	0.00344988
SLC35E3	1.322685858	1.075354146	1.626903924	0.008101798	0.018431061
LMOD3	0.397737588	0.200979931	0.787119335	0.008113724	0.014745422
P2RY12	0.814629568	0.699874555	0.9482004	0.008131221	0.000381674
CEACAM19	1.207299008	1.050003238	1.388158475	0.008167848	0.042019521
ADAMTS4	1.164334682	1.040165665	1.303326284	0.008183668	0.003136881
HDGF	1.355125159	1.081808734	1.697494334	0.008189503	0.002159627
CD164L2	0.849153314	0.752155511	0.95865993	0.008238037	0.003911779
LDHD	0.860039231	0.769004717	0.961850379	0.00825733	0.033457015
HLA-DRA	0.868803673	0.782698197	0.964381706	0.008265133	0.002635708
MAOB	0.8602451	0.769309706	0.96192941	0.008269104	0.029537858
TRIP13	1.167457838	1.040723815	1.309624883	0.008271378	0.00053381
SNX8	1.274022883	1.064378634	1.524959497	0.008287618	0.026319278
RNF4	1.520088832	1.113896498	2.074402837	0.008291885	0.018379292
ASAP1	1.29388666	1.068567395	1.566716987	0.008307223	0.00781798
ITGAL	0.835142867	0.730563295	0.954692925	0.00830985	0.028211258
FUBP3	1.511927352	1.112192894	2.055330806	0.008322208	0.009531652
CRBN	0.635978328	0.454406323	0.890103006	0.00832226	0.040241154
SLC22A31	0.91598964	0.858168619	0.977706482	0.008347981	0.020616977
CGNL1	0.832266161	0.726101633	0.953953182	0.008363533	0.03623257
LPAR6	0.778718381	0.646615857	0.937809227	0.008365903	0.041478316
CCDC51	1.45851774	1.101640454	1.931005702	0.008387576	0.010162199
MEI1	0.797984492	0.67471386	0.943776745	0.008392597	0.032356345
NDUFA12	1.462467768	1.102335151	1.94025562	0.00840217	0.023141942
ANKRD55	0.573653175	0.379386407	0.867395244	0.008430617	0.001130107
IGF2BP2	1.149632268	1.036275846	1.275388552	0.008469897	0.005934177
CATSPERE	0.636034813	0.454095292	0.890870905	0.008485042	0.045232191
THG1L	1.435440883	1.096552088	1.879063066	0.008518931	0.0346996
SLC39A9	1.542203643	1.116720887	2.129799939	0.008532636	0.019872341
TCP10L2	0.113201552	0.022321851	0.574082822	0.008540484	0.002173545
IRF8	0.835454406	0.730682089	0.955249998	0.008548158	0.003144237
POU2AF1	0.865822393	0.77759712	0.964057603	0.008601003	0.01913061
OR51E1	1.299582027	1.068820013	1.580166375	0.008607826	0.011586191
COL11A1	1.089944838	1.02210089	1.162292061	0.008623241	0.019487343
MEIS3	1.274203957	1.063415997	1.526773838	0.008629906	0.010513542
TRAF7	1.42940441	1.094879543	1.866138591	0.008631349	0.040172066
TMSB10	1.288512576	1.06635044	1.556959697	0.008657329	0.011845948
SYNE1	0.818943726	0.705457504	0.95068636	0.008679438	0.016714141
SCPEP1	0.812740065	0.696106954	0.94891512	0.00870554	0.010442898

PSMA1	1.506697765	1.108975059	2.047059703	0.008755971	9.28E-06
ACSS3	0.783988039	0.653530366	0.94048766	0.008774956	0.03068985
XRCC2	1.204401238	1.047949103	1.384210683	0.008801769	0.009670897
HNRNPA2B1	1.46966075	1.101808396	1.960325159	0.008803572	0.005348668
SIAH2	1.410594028	1.0903785	1.824848447	0.008829535	0.003288522
TEX10	1.472586415	1.102237533	1.967371538	0.008829601	0.042545858
GALNT4	1.297700296	1.06773564	1.577193824	0.008830849	0.035251011
THYN1	0.699600086	0.535419117	0.914125524	0.00884639	0.047180437
DOK1	0.745334144	0.59808398	0.928837764	0.008862102	0.029947257
TTC21A	0.758718356	0.616968165	0.933036058	0.008875136	0.020696081
ARPC5	1.497357859	1.106639602	2.026025957	0.008876769	0.016175406
B3GALNT1	1.219773706	1.051123902	1.415482886	0.0088788	0.045337034
RAD54B	1.377901097	1.083436044	1.752398254	0.008968449	0.03134206
PHACTR1	0.805935892	0.685525928	0.947495398	0.008969577	0.026314381
COLGALT1	1.317181084	1.07127073	1.619540197	0.008976915	0.021886459
CASZ1	0.794812815	0.669034691	0.944237152	0.008981278	0.002653037
CPAMD8	0.878410478	0.796998203	0.968138905	0.008989249	0.004747858
TMEM243	0.778306443	0.644879982	0.939339002	0.008996817	0.019899374
YY1	1.656737618	1.134213684	2.4199845	0.009017165	0.009778854
ERH	1.43658648	1.094543103	1.885517994	0.009025919	0.013037463
TBC1D10C	0.816380616	0.701017771	0.950728125	0.009054214	0.039079061
ARF6	1.449693839	1.096703945	1.916298593	0.009098446	0.0230546
OR2A7	0.751210625	0.605895689	0.931377155	0.009104559	0.01188149
LARP6	1.214671367	1.049477803	1.405867304	0.00912159	0.016436755
RBP5	0.795284111	0.669456155	0.944762121	0.009145118	0.008492694
SLC52A3	1.201934268	1.046669281	1.38023157	0.009152778	0.023406808
CDHR2	1.145731634	1.034310042	1.269156175	0.00915454	0.01756831
CST5	0.823088509	0.710978471	0.952876523	0.009158136	0.035659922
CD69	0.846167368	0.746272156	0.959434449	0.009159545	0.019003817
EPHB2	1.177364401	1.041270901	1.331245242	0.009180674	0.000819877
NIP7	1.479785274	1.101769078	1.98749856	0.009216422	0.018049263
ARHGEF6	0.809876423	0.690993726	0.94921241	0.009228297	0.003323006
BRCA2	1.327664781	1.072539283	1.64347712	0.009236751	0.012736995
NDUFS2	1.452679798	1.096639083	1.924314597	0.009240141	0.028339161
TAF2	1.365923942	1.080054571	1.727457357	0.009247798	0.020227789
AMIGO1	0.796434333	0.670980425	0.945344488	0.009250306	0.018320138
TNFRSF11A	1.262225857	1.059155505	1.504230594	0.009264082	0.026941885
HSDL1	0.734743514	0.582514587	0.926754529	0.009264931	0.002542617
TTLL10	0.755472314	0.611580697	0.933218493	0.009293164	0.001880678
YBX1	1.437916573	1.093503297	1.890807351	0.00932763	0.025777388
EME1	1.213267785	1.048724953	1.403627056	0.00932887	0.006664012
TESK2	0.719262841	0.5608879	0.922357274	0.009406956	0.001200422
METTL21A	1.463081361	1.097792706	1.949919194	0.009415314	0.038985243

CKS2	1.207726403	1.047194916	1.39286683	0.009495392	0.027262231
UBE2E2	1.282947502	1.062733311	1.548793357	0.009508166	0.036455592
RNF175	0.74684592	0.598938846	0.93127843	0.009535249	0.002155575
TSKU	1.175578084	1.040199081	1.328576286	0.00956057	0.020388625
GFI1B	0.318923635	0.134360356	0.757011134	0.009565716	0.000312758
NCKAP1	1.413874571	1.08799019	1.837370705	0.009574482	0.037324218
RWDD4	1.632276745	1.126687977	2.36474288	0.009579645	0.026652494
ADAMTSL5	1.197752077	1.044836949	1.373046808	0.009615617	0.007117167
STIL	1.229011682	1.051375781	1.436660175	0.009626571	0.019065377
ZNF526	1.478191647	1.099570436	1.987185608	0.009634271	0.008919572
CD6	0.816363878	0.700076321	0.951967609	0.009659521	0.046821916
ENPP4	0.804393465	0.682131994	0.94856839	0.009662436	0.009054253
WDR1	1.494780096	1.102369934	2.026876338	0.009674206	0.035639953
HPRT1	1.299502309	1.065544046	1.584830076	0.009686476	0.014394747
TUBG1	1.278184857	1.061278161	1.539423488	0.009687774	0.00476817
PITX1	1.110824408	1.02578084	1.202918612	0.009700349	0.012905057
MRPL11	1.37135683	1.079356928	1.742351864	0.009735794	0.016389987
PSMD7	1.429303771	1.090131097	1.874003296	0.009756139	0.027576844
POLQ	1.223981783	1.050009663	1.426778683	0.009770967	0.007588786
TSC22D2	1.269748548	1.059264883	1.522056854	0.009806463	0.019499758
HLA-DOA	0.874970963	0.7906102	0.968333303	0.009821831	0.007194223
FAM83H	1.238954072	1.052971725	1.457785767	0.009824468	0.023018025
SLIRP	1.384897143	1.081500135	1.773407174	0.009852196	0.02488052
MRPS10	1.375502404	1.079593732	1.75251746	0.009890728	0.014049799
LRRC3B	0.257564553	0.091884648	0.721986757	0.009897766	0.003336842
AK6	1.483524913	1.099269543	2.002098739	0.009915281	0.000891993
MIX23	1.3142226	1.067641033	1.617754459	0.009956971	0.004597904
ABCD1	1.303743607	1.065568407	1.595155582	0.009966402	0.012429418
TATDN1	1.350946133	1.074623712	1.698320476	0.009983784	0.011193516
LRP11	1.288891886	1.062567761	1.563422453	0.009995973	0.015948532
NPB	11.3839836	1.788223143	72.47142686	0.01001223	0.003931862
CCDC153	0.80377242	0.680643941	0.949174838	0.010029255	0.004020846
TBK1	1.488729539	1.099634616	2.015501885	0.010040168	0.02374749
ZFAND2A	1.274282134	1.059537582	1.5325506	0.010048439	0.015727913
PNP	1.297236287	1.064032198	1.58155175	0.010058502	0.011645133
RIMS2	1.227787612	1.050151973	1.435470731	0.010062382	0.002181741
FAM91A1	1.383640043	1.080499861	1.771827872	0.010064737	0.0291322
MRPS9	1.438859831	1.090626859	1.898282256	0.010065235	0.010010623
LSM3	1.576805555	1.114679995	2.230519765	0.010068136	0.000931577
CDA	1.095289677	1.021901242	1.173948545	0.010104899	0.01485079
VRK2	1.421094891	1.087204843	1.857525473	0.010115799	0.00237175
WBP2NL	0.586618092	0.390672232	0.880842706	0.010120126	0.036463849
PCSK9	1.11588182	1.026355181	1.213217664	0.010180997	0.027039677

GOLT1B	1.28944001	1.062136427	1.565387926	0.010192633	0.000318578
OLFM1	0.855513494	0.759466446	0.963707274	0.010217094	0.003728087
PSCA	1.072937237	1.01680911	1.13216365	0.010227999	0.024582319
RXFP1	0.673243999	0.497649962	0.910795775	0.010289682	0.002451747
RHOH	0.808739926	0.687649835	0.951153095	0.010313903	0.00251813
INVS	1.509902075	1.102081842	2.068634279	0.010315828	0.002202983
COASY	1.418209186	1.085905248	1.85220331	0.010318103	0.003361401
DNAH1	0.786871989	0.655152694	0.945073619	0.010336712	0.021726357
SLC16A13	1.293424867	1.062373119	1.574727236	0.010389743	0.028630528
DMAC2	1.467503755	1.094237284	1.968098968	0.010426661	0.03980327
TDRD10	0.870265873	0.78245322	0.967933508	0.010451282	0.020621966
GINS1	1.183633597	1.040292995	1.346724913	0.010475	0.021265947
TMTC3	1.331227768	1.069334643	1.65726172	0.010475129	0.0028855
FANCC	1.388998802	1.080022173	1.786368577	0.010477867	0.007648188
ZNF552	0.76445585	0.622342307	0.939021402	0.010481391	0.018597256
NPM1	1.360175507	1.074568937	1.721692622	0.01052681	0.004436937
TRIM15	1.149910779	1.033150526	1.279866551	0.010559457	0.006708719
BCL2L12	1.329462817	1.068730999	1.653803795	0.010565168	0.001422042
BEND6	1.241186272	1.051621265	1.464922223	0.010612724	0.022840801
STK3	1.429037402	1.086608409	1.879377962	0.010641842	0.019143228
LRRC61	1.305858594	1.063974853	1.602732117	0.010671274	0.011040046
L3MBTL3	1.307147536	1.06420186	1.605555059	0.010677332	0.03312034
BOP1	1.239551999	1.051148963	1.461723515	0.010680157	0.004009165
RFC2	1.322935681	1.067133701	1.6400558	0.010691178	0.001209746
HAT1	1.441826186	1.088500449	1.909840966	0.010734735	0.023208663
GART	1.378561004	1.077195394	1.764239294	0.01074792	0.011299668
CORO1A	0.82645082	0.713796492	0.956884723	0.010790257	0.023863935
NRL	0.607461358	0.41400007	0.891326664	0.010832913	0.00058406
CCL14	0.781802731	0.646926055	0.94479965	0.010844947	0.01627478
MPLKIP	1.457502791	1.090739604	1.947590769	0.010857681	0.004416501
POGLUT2	1.257486381	1.054198564	1.499975482	0.010876981	0.004252389
MICU3	0.749448016	0.600256669	0.935720264	0.010878331	0.030358643
SNX22	0.754821915	0.607889758	0.937268833	0.010881161	0.011021345
BEST3	1.524503432	1.10191844	2.10914949	0.010898144	0.000169729
STIMATE	0.576731678	0.377496828	0.881118471	0.010919486	0.024110406
TES	1.331672559	1.068037621	1.660383276	0.010935686	0.042433127
GLCCI1	0.833659622	0.724603035	0.959129802	0.010980498	0.002652394
TLR7	0.817874079	0.70044686	0.954987518	0.011009737	0.000836556
ACRV1	1.486333741	1.094979638	2.017560796	0.011023858	0.01443039
KPNB1	1.363016795	1.07328356	1.730963608	0.011085483	0.00073996
HSD17B13	0.847408039	0.745734175	0.962944181	0.011117104	0.011962062
STX16-NPEPL1	0.760022897	0.614930125	0.939350312	0.011122124	0.011435305
FAAP24	1.277547215	1.057347213	1.543605417	0.011158644	0.041151132

AKNA	0.79066919	0.659488678	0.947943141	0.011163411	0.019361065
PFDN2	1.322588228	1.065695563	1.641406496	0.011167448	0.035910133
RAB9B	0.705298397	0.538510953	0.923743195	0.011207387	0.030066719
IL24	0.800535462	0.673999623	0.950826978	0.011264914	0.019620902
U2SURP	1.372267277	1.07417372	1.753084668	0.011322923	0.007419746
EREG	1.078097356	1.017124519	1.142725289	0.011354532	0.001130296
ITK	0.806913208	0.683409684	0.952735878	0.011366904	0.031276685
DHRSX	1.290799691	1.059274236	1.572929641	0.01137685	0.000542733
TKFC	1.349897796	1.069951563	1.703090236	0.011401417	0.006203553
HOXC6	1.160609761	1.034081056	1.30262034	0.011438966	0.019394582
RAB3B	1.150352302	1.031907176	1.282392884	0.011520413	0.009813356
STRN4	1.370818468	1.073196234	1.750978257	0.011549334	0.016606117
ARVCF	0.816981799	0.698296468	0.955839376	0.011606139	0.020072094
H2BC12	1.180081502	1.037687926	1.342014604	0.011607787	0.003455462
HSD17B6	0.890092329	0.81314012	0.974326976	0.011612192	0.00254182
WDR75	1.463389956	1.088686776	1.96705812	0.011635206	0.045787887
CHIT1	0.908179339	0.842707613	0.9787377	0.011637882	0.010204874
UFD1	1.395932932	1.077241801	1.808905624	0.011646947	0.00113692
SLC6A8	1.153380419	1.032335626	1.288618116	0.011651395	0.045620889
RFTN1	0.830371554	0.718695536	0.959400585	0.011656142	0.005373613
DNPEP	1.568504627	1.105403553	2.225618652	0.011693106	0.012125916
ACTR3	1.441965231	1.084850911	1.916635463	0.011705265	0.014457998
ZCCHC9	1.544107442	1.101421197	2.164719362	0.011722665	0.006912926
TSPAN7	0.88560925	0.805716426	0.973424046	0.01179042	0.035411399
SYT15	0.544938664	0.339714579	0.874140132	0.011807007	0.016477167
RALGPS2	1.263922781	1.053246349	1.516739932	0.011816422	0.002891414
PTPRC	0.85686444	0.759754251	0.966387049	0.01183311	0.001119641
COX8A	1.390254175	1.07564504	1.7968815	0.011835222	0.030225037
ARPC5L	1.489150254	1.092109059	2.030537574	0.011840233	0.033913917
PDLIM5	1.234907168	1.04773018	1.455523324	0.011871571	0.028007139
ARPC1B	1.334964942	1.065870905	1.671995538	0.011890537	0.014253113
ZNF154	0.790926403	0.658806869	0.949541669	0.011897254	0.011211616
KCNQ1	0.87995664	0.796487514	0.97217304	0.011904081	0.000421738
HEMK1	0.709893407	0.54351553	0.927201931	0.011913896	0.040677936
GMFG	0.813970156	0.693298066	0.955645844	0.011933506	0.011318425
NT5C1A	0.763556706	0.618681782	0.942356571	0.011969426	0.022529184
UBE2Z	1.530393755	1.098033746	2.132999147	0.012002882	0.004678215
ZCRB1	1.481580345	1.090013094	2.013810964	0.012060658	0.00679377
PSMA3	1.431309879	1.081719943	1.89388019	0.012081802	0.010568856
HLA-DRB1	0.878463571	0.793910218	0.97202206	0.012089481	0.001744904
CHIA	0.894533168	0.81994081	0.975911405	0.012112761	0.004927174
P3H4	1.20083874	1.040823846	1.385454114	0.012129705	0.002495948
RBM8A	1.459477887	1.086127887	1.961164729	0.012140782	0.047330946

PHB2	1.37135945	1.071362698	1.755359547	0.012168312	0.035887246
MRPS27	1.455299275	1.085305716	1.951428017	0.012179233	0.032419868
ATG9A	1.480456256	1.089281406	2.012106984	0.012202545	0.017671334
AATF	1.408963539	1.077554887	1.842298965	0.012213969	0.036311818
ABCC12	0.329692468	0.138420129	0.785269629	0.012214864	0.005994648
HELLS	1.218312584	1.043867128	1.421910427	0.01226337	0.00019169
RAB1B	1.491067081	1.090732854	2.038336914	0.012264948	0.040635853
BOLA3	1.322588375	1.062599269	1.646189736	0.012290817	0.015333858
SSB	1.416952526	1.078613477	1.861421635	0.012293479	0.01320845
CXCR6	0.814334503	0.693292418	0.956509354	0.012365627	0.031125315
SLC25A32	1.427228553	1.080055212	1.885997419	0.012366483	0.002118992
ENTPD7	1.261718018	1.051530032	1.513920011	0.01240564	0.027303477
RCSD1	0.811181563	0.688375193	0.95589663	0.012471966	0.001842835
SULT1A1	0.819369485	0.700820614	0.957971754	0.012474079	0.014871438
DDX59	1.51571214	1.093614992	2.100724029	0.012513225	0.041578646
ARHGAP44	0.84273228	0.736830291	0.963855184	0.012515681	0.010140126
CHAF1A	1.264416248	1.051755805	1.520075707	0.012523609	0.009076027
SRP72	1.399591453	1.074938865	1.82229548	0.012538055	0.012147719
ANKRD29	0.874491204	0.787011383	0.971694798	0.012634524	0.005866205
ORC6	1.196685157	1.039087064	1.378186116	0.012697641	0.033490297
TPRKB	1.404082067	1.075060695	1.833800139	0.012729498	1.47E-05
TMEM252	0.659866773	0.475741096	0.915254456	0.012757597	0.019346027
KCTD5	1.317615117	1.060487345	1.637086576	0.012768053	0.006541316
OGDH	1.366837838	1.068783894	1.748010692	0.012773987	0.047788522
PITPNC1	1.194137737	1.038441194	1.37317832	0.012804357	0.00582403
TIMM22	1.548050436	1.097305546	2.183949733	0.012817006	0.003439291
EIF4E	1.538528462	1.095614092	2.160495968	0.012878336	0.013153802
ABL1	1.36723624	1.068403076	1.74965327	0.012926619	0.022440885
FCER1A	0.902679757	0.832630856	0.978621845	0.012980528	0.042479345
AHCTF1	1.371404295	1.068903852	1.759512548	0.012990259	0.035974515
MRPL17	1.408403407	1.074911432	1.845361484	0.012994254	0.002137957
KCP	1.182188811	1.035898575	1.349138246	0.01301864	0.00447485
FNDC3B	1.302098261	1.057202116	1.603723503	0.013019398	0.008167522
CRYBA4	0.526275834	0.317074841	0.873504352	0.013024219	0.020901116
NME1	1.26650021	1.050996501	1.526192313	0.013041794	0.014114188
CD74	0.874141667	0.786068791	0.972082422	0.013045234	0.006327289
PIFO	0.873268774	0.784657868	0.971886452	0.013052613	0.003836912
SIT1	0.83990106	0.731762936	0.96401957	0.013099514	0.020960042
TEX15	1.302133637	1.056928234	1.604226242	0.013135059	0.027353629
SRM	1.347603121	1.064537749	1.705936847	0.013144524	0.004236493
CNOT9	1.501785779	1.088964213	2.071106192	0.013151116	0.005176475
DCPS	1.395089388	1.072224291	1.815174695	0.013167235	0.017165817
PLA2G12B	0.903832666	0.834386281	0.979059109	0.013182777	0.028070659

NKIRAS2	1.388057595	1.070975153	1.799018288	0.013205168	0.015516151
MCOLN2	0.82077854	0.702067831	0.959561715	0.013217956	0.004855971
LHFPL5	1.387589516	1.070848108	1.798018457	0.013222205	0.000462085
MAFF	1.282745322	1.053377922	1.562056246	0.01323679	0.010242576
ZAP70	0.834554522	0.723262438	0.962971688	0.013261992	0.049517506
TSNARE1	0.802885108	0.674828948	0.955241322	0.013267785	0.036094794
TLE2	0.837169049	0.727297919	0.963638144	0.013287703	0.043504355
TMEM65	1.251046912	1.047777315	1.493750965	0.013291508	0.026513116
CEACAM21	0.818896738	0.699089981	0.959235414	0.013299199	0.001001224
CD27	0.860563672	0.764081722	0.969228569	0.013319256	0.020783381
FRMD8	1.407041595	1.073517995	1.844185249	0.013365053	0.004280381
MAP4K1	0.826115879	0.710070262	0.961126642	0.01338555	0.016388398
MRPS5	1.478457939	1.084150436	2.016175805	0.013493795	0.025273429
IGFBPL1	0.876967318	0.790206242	0.973254368	0.013510994	0.017376078
LEO1	1.403603136	1.072403433	1.837090132	0.013548565	0.047773682
NDUFB3	1.438146136	1.077771274	1.919019701	0.013554936	0.001997849
C1orf112	1.313745034	1.057683496	1.631798189	0.013626575	0.04717848
LAD1	1.211812149	1.040245129	1.411675618	0.013642811	0.000706189
TTC23L	0.649805888	0.461315995	0.915311189	0.013654627	0.001640024
MPC1	0.733604582	0.573305261	0.93872448	0.013791954	0.02275852
LINGO3	0.639940661	0.448578556	0.912937199	0.013799089	0.003040394
DNAJC22	1.138850012	1.026886516	1.263021112	0.013799297	0.015606333
FGD2	0.806660019	0.679840831	0.957136372	0.013817638	0.009118062
AFAP1	1.281868157	1.051887951	1.562130234	0.013839986	0.014806373
LYRM9	0.741934226	0.584941777	0.941061858	0.013864734	0.008306713
PCNA	1.257181272	1.047609313	1.508677644	0.013899631	0.011496352
CHPF	1.245001559	1.045523295	1.48253883	0.013907715	0.040194561
E2F8	1.191013261	1.036099742	1.369088835	0.013940587	0.005097113
RTN4	1.428202824	1.074960806	1.897523422	0.013948588	0.032828637
SNRNP27	1.529929237	1.09004129	2.147334685	0.013954999	0.038871335
TYRP1	0.830944583	0.716813287	0.963247911	0.01402245	0.007128522
ID1	1.134648025	1.025832455	1.255006249	0.0140577	0.014390766
ARMT1	1.374580588	1.066279358	1.772023231	0.014079905	0.02163916
IMP4	1.493308351	1.08423722	2.056717655	0.014082846	0.044918669
CD2	0.852070372	0.7498219	0.968261821	0.014109292	0.008033559
FKBP5	1.169669238	1.032065106	1.325619982	0.014119313	0.004814704
EI24	1.417373402	1.072739144	1.872726816	0.014129679	0.026068206
TMEM158	1.147643101	1.02809688	1.281090054	0.014140204	0.02163399
SEPTIN10	1.34302279	1.061000924	1.700008146	0.014193585	0.046781424
HSF1	1.343864144	1.061082901	1.702007295	0.014213448	0.015507278
PTBP3	1.382382861	1.067062713	1.790881034	0.014231931	0.005152827
TAPT1	0.773696901	0.630171074	0.949911729	0.014253711	0.018529322
ATL3	1.401244748	1.069862607	1.835270091	0.014266509	0.005419729

CPED1	0.818802143	0.697806951	0.960777115	0.014269018	0.028026194
BLVRB	1.220443783	1.040605674	1.431361625	0.014311811	0.023051965
PSRC1	1.207738527	1.038425454	1.404657737	0.014315973	0.004890778
AMFR	1.350843565	1.061802126	1.718567228	0.014359547	0.027878965
PAQR4	1.21022266	1.038756033	1.409993145	0.014374812	0.003442877
FIGN	1.238256913	1.043448895	1.469434862	0.014406803	0.041057988
GAS2L3	1.254710863	1.046166333	1.504827005	0.014420938	0.032862162
TAGAP	0.829487467	0.714105321	0.963512578	0.014430644	0.033940609
PLPP4	1.13655234	1.025715238	1.259366317	0.01448732	0.003335178
PRPF19	1.412799014	1.070878633	1.863891009	0.014511759	0.037682076
RHOQ	0.693240713	0.516652096	0.930186271	0.014589479	0.032160813
CCDC152	0.712499648	0.542772439	0.935301264	0.014615295	0.006307895
RFC3	1.247772069	1.044633846	1.490412302	0.014622062	0.025969787
LRRN3	0.786043695	0.647898118	0.953644828	0.01463542	0.017171153
ARRDC5	0.671662521	0.48789677	0.924643429	0.014672904	0.020007699
TNFSF8	0.80165396	0.671215003	0.95744146	0.014689142	0.001363472
TMEM273	0.815597314	0.692403612	0.960709862	0.014698641	7.36E-05
ZC3H12C	1.27804582	1.049357232	1.556572985	0.014731538	0.005724266
PCDHB8	1.157292999	1.029078928	1.301481402	0.014751397	0.015600357
CD300LF	0.852770186	0.75030175	0.969232699	0.014751437	0.006598888
HLA-DQB1	0.894157421	0.81721489	0.978344255	0.014815496	0.002171747
OTUB1	1.465664431	1.077572421	1.993529329	0.014850325	0.000740164
UPK2	1.131424767	1.02438295	1.249651806	0.014890196	0.018140183
HDGFL3	1.23995223	1.042827266	1.474339597	0.014904699	0.01346054
HNRNPD	1.417469165	1.070231019	1.877369275	0.014958231	0.013788622
KLHL35	0.831442184	0.716564345	0.964736957	0.014967397	0.013447006
SNX20	0.821845272	0.701652389	0.962627167	0.015011055	0.017458155
CNGA3	0.879262808	0.792651191	0.975338326	0.015019068	0.001116072
TMEM44	1.264139682	1.046519057	1.527013889	0.015028793	0.0326888
MTDH	1.342836249	1.058693106	1.703240706	0.015092425	0.021325725
COPS6	1.37816934	1.06394045	1.785203984	0.0151241	0.016833327
ZNF671	0.792689284	0.657199308	0.956112238	0.015130478	0.026200173
SLC35F2	1.224668594	1.039814321	1.442385564	0.015198372	0.008942155
SH2D3A	1.265826435	1.046432138	1.531218802	0.015210887	0.00054047
MARK2	1.369167853	1.062308149	1.764667449	0.015231948	0.02212176
RAD54L	1.167075865	1.030098464	1.322267843	0.015287051	0.024134774
PRIMPOL	1.424701086	1.070298386	1.896455429	0.015287276	0.044702163
TPSB2	0.903087991	0.831648968	0.980663658	0.015335872	0.012566739
MTA2	1.454962477	1.074458106	1.97021717	0.015339623	0.013317257
SLC14A1	0.754307546	0.600484364	0.94753487	0.015388499	0.004154175
PSMD9	1.546947885	1.086778646	2.20196428	0.015438358	0.006561841
CNTN3	0.822679909	0.702465184	0.963467298	0.015447454	0.007593614
BANK1	0.825911559	0.707454147	0.964203696	0.015458429	0.033082968

CABLES1	0.840654193	0.730385119	0.967571017	0.01554234	0.002458672
FETUB	1.248734608	1.043003765	1.495045535	0.015590479	4.80E-06
RNPEP	1.381725888	1.063195683	1.795686777	0.015593855	0.03596017
TAS1R1	0.784083919	0.643784607	0.954958515	0.015600213	0.028960904
ADAMTS15	1.18940022	1.033347298	1.369019773	0.015644998	0.036409334
CCNF	1.22047765	1.03839783	1.434484598	0.015646574	0.037629953
SEC13	1.493295991	1.078669821	2.067298885	0.015679288	0.046475729
FYTTD1	1.405669695	1.066313317	1.85302693	0.015717343	0.042696708
CENPC	0.679564481	0.496661469	0.929824261	0.015744475	0.024951847
SUMO2	1.478078242	1.076291272	2.029855064	0.015769085	0.006304311
TBL2	1.419250653	1.068097721	1.885850306	0.015769128	0.002867491
ACSBG1	0.536620463	0.323735629	0.889495922	0.015773399	0.044140791
DNAJC10	1.318259225	1.053354892	1.64978337	0.015773719	0.019474616
CIBAR2	0.876529724	0.787584746	0.975519602	0.015780055	0.045723638
EXOSC2	1.409399802	1.066612574	1.862351757	0.015799487	0.044587472
FLT3	0.71183701	0.540123854	0.938140253	0.015806999	0.002154879
MCM8	1.236461103	1.040539714	1.469272185	0.015889109	0.004306749
NCKAP5	0.814551283	0.689443527	0.962361334	0.015913253	0.038330757
ACER1	0.267123488	0.091295785	0.781579981	0.015958727	0.008637159
ATP8B2	0.825198794	0.70580204	0.964793259	0.015977151	0.000741478
ITPKA	1.092702662	1.016660297	1.17443271	0.015999278	0.021447203
ECRG4	0.872112023	0.780218442	0.974828765	0.016008585	0.044340128
RBM15	1.487142745	1.076674705	2.054096315	0.016027874	0.03122255
EPB41L5	0.802828304	0.671410959	0.959968373	0.016044229	0.007659951
ARHGAP25	0.795944379	0.66096001	0.958495893	0.016082391	0.008538342
RAB39B	0.775494926	0.630471256	0.953877555	0.016084631	0.001988818
PRKCE	0.747262204	0.589437706	0.947344893	0.016091506	0.04930662
SNRPD1	1.332392882	1.054641811	1.683292633	0.016128222	0.006562944
RAI2	0.833756687	0.718951992	0.966893786	0.016156159	0.027829528
LRRC56	0.827105959	0.708467788	0.965610969	0.016263201	0.024227079
PSMB1	1.373062292	1.060153356	1.77832768	0.016277334	0.036196191
CLEC12B	0.45914663	0.243311455	0.866443495	0.016286602	0.000496648
YDJC	1.262723341	1.043775782	1.52759842	0.016352845	0.004429977
MTIF2	1.422548943	1.066857449	1.896828388	0.016359172	0.031874899
ABI3BP	0.856188616	0.754237147	0.971921032	0.016383869	0.006979824
OASL	1.154754769	1.026711415	1.298766679	0.016414044	0.024559175
KCNK16	0.606963229	0.403679072	0.912616945	0.016422611	0.007780777
KLF4	1.151939161	1.02623277	1.293043712	0.016431792	0.021905237
KLK13	0.88972245	0.808708346	0.978852318	0.016450291	0.004262322
SPATA6L	0.772417696	0.625482314	0.95387045	0.016455587	0.014750448
TFF1	1.049952035	1.008952513	1.092617602	0.016461423	0.03779228
PHF1	0.728547005	0.562395479	0.94378557	0.016482779	0.007514088
TERT	1.259323627	1.042958669	1.520574155	0.016517472	0.017996252

FBL	1.352975175	1.056649406	1.732402265	0.016536642	0.012970201
STX18	1.527572436	1.080069239	2.160488848	0.016599553	0.012029795
MTMR8	0.685135105	0.502791318	0.93360823	0.016616199	0.016234456
ERVFRD-1	0.521598105	0.306193476	0.888538144	0.016629816	0.033377787
SLC39A1	1.391216394	1.061634159	1.823116786	0.016686884	0.028291975
SHISA5	1.446036174	1.06901066	1.956033455	0.016714481	0.039119273
KRT80	1.149692078	1.025473044	1.288958185	0.016796053	0.005779214
HAGH	0.724141209	0.555787015	0.943491799	0.016810556	0.03366526
CAMTA1	0.695875801	0.516867992	0.936879701	0.016863076	0.023611341
TMEM241	0.753362917	0.597176026	0.950399313	0.016888523	0.021002084
PMAIP1	1.145309125	1.02465184	1.28017434	0.016906796	0.00553465
SASS6	1.252585737	1.041251471	1.506812785	0.016907216	0.000775552
CAMK2N1	1.13520944	1.023001151	1.259725341	0.016930482	0.020786568
TTLL2	0.645779379	0.451046536	0.924585322	0.016932133	0.001755097
MRPL16	1.511159605	1.076777416	2.120775675	0.016951162	0.006047554
TRIM22	0.839691192	0.727493134	0.969193062	0.016960578	0.01770099
SLC7A8	0.867152766	0.771381679	0.974814337	0.016979217	0.002183876
SLAMF9	1.169602797	1.028378501	1.330221025	0.01702428	0.029635904
HMCN2	0.702090127	0.524995571	0.938923249	0.017083291	0.035037841
TEPP	0.842930623	0.732489129	0.970023999	0.017092338	0.001179684
ICAM5	0.879200067	0.790900449	0.977357845	0.017122174	0.020291661
FCAMR	0.691448417	0.510531621	0.936476594	0.017124294	0.001299247
SEMA4G	1.152564666	1.02557443	1.29527928	0.017127935	0.016216811
PRICKLE2	0.797582567	0.662232505	0.960596084	0.017141717	0.024548579
LSM12	1.384816464	1.059539441	1.809953047	0.017156107	0.004659875
CLEC12A	0.845374036	0.736213083	0.970720674	0.01725519	0.00456037
CD48	0.858607099	0.757329674	0.973428317	0.017288664	0.006644702
LSM11	1.442718557	1.06673677	1.951218796	0.017343376	0.043734819
TMED10	1.456991824	1.068375756	1.986964945	0.017415668	0.021990722
ASB14	0.646420895	0.451031704	0.926453661	0.017503144	0.019202364
SCN2B	0.724050553	0.554722625	0.945065479	0.017517321	0.011529653
GTPBP4	1.301106237	1.047127731	1.616686666	0.017521101	0.035173185
ZNF662	0.815781317	0.689562978	0.965102796	0.017589576	0.044044008
NIPA2	1.354497653	1.054341257	1.740104431	0.017598116	0.004611703
CMTM5	0.185612783	0.046196134	0.745778975	0.017628068	0.013625961
RASGRF1	0.874290238	0.782463373	0.976893546	0.017650042	0.012914839
DRC3	0.801939075	0.668256855	0.96236391	0.017678849	0.004917895
ARHGAP30	0.833317137	0.716746252	0.968846994	0.017711363	0.045581005
MZT2B	1.293253217	1.04554024	1.599655201	0.017765539	0.020534706
TIMM9	1.397025445	1.059569409	1.841955872	0.017780992	0.009665049
FA2H	1.125130987	1.020604939	1.240362151	0.01779086	0.019599878
DAD1	1.43373939	1.064273129	1.931467198	0.017803922	0.0115263
ASTL	0.554278311	0.340222076	0.903011496	0.017804859	0.014419692

FRMPD2	0.649373255	0.454318159	0.928172506	0.017839031	0.03598591
TONSL	1.19524426	1.031191863	1.385395766	0.017897528	0.017065798
C12orf29	1.374179301	1.056245366	1.787812579	0.017906372	0.001002363
LATS2	1.305768863	1.046905935	1.628639467	0.017953305	0.035881932
FRS3	0.777295883	0.630906334	0.957652281	0.017961968	0.015630681
P2RX2	0.68728014	0.503777635	0.937623979	0.017963256	0.010468516
SGPP1	1.254868137	1.039565461	1.514761793	0.01807847	0.043493615
KIAA0408	0.411584196	0.197130669	0.859336353	0.018099329	0.012432255
ARHGAP10	1.297262732	1.045408261	1.60979271	0.018118115	0.030922112
PDE6B	0.832002418	0.714242661	0.969177649	0.018174867	0.001866256
SYNGR4	1.184302617	1.029190863	1.362791626	0.018192152	0.009934338
LRMDA	0.757448921	0.6014587	0.953895701	0.018218496	0.013688791
DNAH5	0.855913664	0.752166332	0.973971008	0.018274127	0.030018399
HCFC2	0.716350914	0.542986291	0.945067383	0.0182942	0.006235655
ELP4	1.503964877	1.071455958	2.111062367	0.018329216	0.04441779
PDAP1	1.357344649	1.053002197	1.749649241	0.018341046	0.003134883
PTX3	1.162362003	1.025699227	1.317233542	0.018394665	0.011788987
SLC35D1	1.3212064	1.047998106	1.665638843	0.01844299	0.015886508
TNFRSF13C	0.855405062	0.751202056	0.974062589	0.018449179	0.010953165
TMEM33	1.362783718	1.053420292	1.762999512	0.018467737	0.028154756
HLA-DPA1	0.885160613	0.799729151	0.979718334	0.018490609	0.00167253
CHORDC1	1.310312022	1.046437332	1.640726628	0.018493428	0.006358359
ARHGAP45	0.804380957	0.671121393	0.964100877	0.018493852	0.00592523
STAM	1.412231709	1.059648678	1.882131731	0.018507517	0.033465541
FHL2	1.148304616	1.023428011	1.28841841	0.018562402	0.001980258
SLC25A1	1.30784537	1.045921776	1.635360837	0.018586149	0.033159572
PRDM2	0.75520765	0.597716172	0.954196357	0.018628522	0.004698132
CD28	0.814387729	0.686341626	0.966322525	0.01864884	0.022172138
UBE2D2	1.60433207	1.082020569	2.378773069	0.018661277	0.004978581
SPN	0.851813194	0.745252605	0.97361044	0.018663748	0.045217306
RNASEK-C17orf49	1.863661011	1.109374589	3.130802161	0.018666005	0.042879308
SLC6A14	1.097235108	1.015586781	1.18544757	0.018673455	0.04769495
HTRA4	0.833143042	0.715568235	0.970036531	0.018677616	0.004743945
FAM222B	1.334753595	1.04923646	1.697965355	0.018705252	0.03650421
KCNJ11	0.86301393	0.76319651	0.975886334	0.018814612	0.037856584
MTX2	1.42423512	1.060283156	1.913116948	0.018836871	0.013884286
TAC4	0.859858898	0.75795265	0.975466377	0.01898125	0.037960841
RCC1L	1.436234605	1.061345714	1.943541876	0.01898946	0.015322268
HMGB2	1.212138319	1.032136525	1.423531936	0.018995663	0.038837087
SCN1A	0.784676399	0.640737096	0.960951153	0.019016931	0.021541013
ITGA4	0.830644238	0.711322127	0.969982267	0.019018901	0.004432313
PNO1	1.374375755	1.05355809	1.792885208	0.019045403	0.013642432
RIIAD1	0.804080147	0.670077807	0.964880311	0.019061521	0.005639413

SPNS3	0.764233613	0.610368438	0.956886004	0.019068577	0.022915379
SLC22A23	0.863028845	0.763010467	0.976158022	0.01908177	0.002646764
SPRR3	1.095431545	1.015007463	1.182228028	0.019137366	0.032016112
MC1R	1.237728272	1.035456364	1.479513119	0.019145942	0.023198838
GIMAP1	0.796451243	0.658297151	0.963599161	0.019209398	0.04617325
GIMAP7	0.83574315	0.719146057	0.971244444	0.019255222	7.78E-05
CYP4Z1	0.770304992	0.619076987	0.958474943	0.0192681	0.001678325
SKAP2	1.238875942	1.035374856	1.482374803	0.019300645	0.019349833
FHL3	1.315339913	1.045306553	1.655130816	0.019391898	0.021446167
CD37	0.854700346	0.74926802	0.974968452	0.019420611	0.022092097
PIP4P2	1.262833806	1.03833386	1.535873272	0.019458496	0.002641096
FLRT1	1.364493204	1.051314357	1.770965736	0.019485147	0.037850497
SLC2A11	0.753718717	0.594546768	0.95550415	0.019489738	0.029336904
MTRNR2L12	1.179429914	1.026843192	1.354690698	0.019558615	0.014475731
DSP	1.120541909	1.018413447	1.232912011	0.019586872	0.035050434
PIK3IP1	0.802918722	0.667755484	0.965440926	0.019599684	0.009469863
PIGR	0.942657079	0.897029532	0.990605478	0.019656363	0.031683385
RP11-12J10-3	0.511099812	0.290757857	0.898421181	0.019692465	0.017266672
PHKA1	1.241157537	1.035077977	1.488266649	0.019693883	0.02544443
PPP1CC	1.433841192	1.059089076	1.941197025	0.019733288	0.015670882
ST6GALNAC4	0.820822888	0.695157071	0.969205727	0.019864819	0.020552687
CARMIL3	0.762003178	0.60619576	0.957856986	0.019865178	0.041249971
DLD	1.359787983	1.049774741	1.761352493	0.019917039	0.022402859
ANGPTL6	0.672571318	0.48143635	0.93958875	0.020058012	0.004329475
NTAQ1	1.290261483	1.040825015	1.599476062	0.020070904	0.041106833
KCNA5	0.730309144	0.560272152	0.951950661	0.020120103	0.026241262
SLC13A5	1.217981781	1.031349577	1.4383868	0.020141105	0.039106366
EIF1AD	1.458124973	1.060757931	2.004348378	0.020162347	0.016082422
GOPC	1.391429267	1.052945567	1.838723164	0.020193626	0.046979594
PACS1	1.293633055	1.040931088	1.607682297	0.020247665	0.007282657
AIG1	1.344222166	1.047088663	1.72567357	0.020287377	0.022644132
GALNT13	1.164810958	1.024002126	1.32498218	0.020298722	0.029229417
NDUFA9	1.370947672	1.050227573	1.789609764	0.020319819	0.018925586
RUSC1	1.29311844	1.04070164	1.606757629	0.02033912	0.028900698
FAT1	1.168305245	1.024362491	1.33247474	0.020407478	0.01535144
MLLT6	0.80505943	0.670221089	0.967025205	0.020423254	0.015415041
MCM7	1.207384375	1.029567815	1.415911617	0.020425347	0.023627826
NFIX	0.869361499	0.772318447	0.978598166	0.020438158	0.042084389
TAP1	1.180098471	1.025879482	1.357500979	0.020474352	0.047515105
DEFB1	1.072429395	1.010845136	1.137765586	0.020479239	0.011607983
PRMT5	1.297818927	1.04094541	1.61808098	0.020527027	0.015305311
CACNA1D	0.847820496	0.737294896	0.974914646	0.020534001	0.043073699
ZKSCAN4	0.715385403	0.538792331	0.949858128	0.020578953	0.019594072

PLEKHD1	0.684660642	0.496798868	0.943561316	0.020614903	0.001806804
TIMM17A	1.412248409	1.054284186	1.891753281	0.020646053	0.006360538
EDA2R	0.851909244	0.7437679	0.975773974	0.020665291	0.044216284
FOLR1	0.927576963	0.870307416	0.988615064	0.020771579	0.010050191
MYO1H	0.484520052	0.262117542	0.895627506	0.020798387	0.023811151
NUP210L	0.803778735	0.667870586	0.967343477	0.020817422	0.025830956
ATP6V1FNB	1.20002714	1.028094385	1.400712968	0.020825194	0.00281211
SLC27A1	0.800792143	0.663274962	0.966820842	0.020834636	0.041973795
RP11-403P17-5	0.280890064	0.095680689	0.824609739	0.020836845	0.019458082
MADCAM1	0.660473551	0.464574206	0.938978758	0.020849023	0.005572998
SAT2	0.748807414	0.585818425	0.957143919	0.020904992	0.044265817
CAPN13	0.91130468	0.842223804	0.986051706	0.020932624	0.013872737
FCHSD2	0.730887507	0.560041888	0.953851057	0.021011675	0.026848092
RASSF2	0.845416079	0.733010631	0.975058636	0.021057577	0.005778817
RNF26	1.303852249	1.040699102	1.6335468	0.02106576	0.011829395
SEPTIN4	0.766710731	0.61178292	0.960872437	0.021081484	0.042289637
HOXD10	1.186589222	1.025945812	1.372386304	0.021161715	0.030523261
SQLE	1.173966865	1.024294414	1.345509828	0.021170923	0.002000924
SCML2	1.190966806	1.026446597	1.381856531	0.02121677	0.024687842
NDUFA8	1.393254854	1.050762771	1.847380913	0.0212249	0.043384292
CARNMT1	1.385326516	1.049784379	1.828117844	0.021262005	0.030278066
RASAL3	0.834063249	0.714686674	0.973379705	0.021317196	0.022262075
CENPL	1.288160379	1.038213122	1.598281825	0.021408797	0.020822175
ZNF554	0.704699749	0.522955586	0.949605949	0.021463075	0.044674344
ZNF709	0.350618351	0.14344757	0.856990662	0.021538535	0.002752743
PPIC	1.327024246	1.04253462	1.689146159	0.021543081	0.039875437
SLIT3	0.868118217	0.769434755	0.979458275	0.021614239	0.004419745
CD207	0.920767731	0.858144742	0.987960624	0.021618124	0.027425957
TMEM117	1.251781237	1.033363888	1.516364452	0.021708541	0.049292124
ELAVL1	1.573425094	1.068408756	2.317152975	0.021732477	0.013695198
NAIP	0.572096979	0.35508125	0.921746655	0.021744181	0.022931446
UGP2	1.459677694	1.056580568	2.016560815	0.021804882	0.04053286
PHF19	1.26903325	1.035183179	1.555710546	0.021864791	0.030207704
TNS1	0.859168764	0.754594694	0.978235032	0.021889432	0.046247249
EFNA2	1.159171922	1.021600761	1.315268739	0.021933918	0.001111953
CD160	0.609419643	0.398868652	0.931114289	0.022022	0.029176
STEAP1B	1.163898748	1.022014263	1.325480813	0.022122098	0.008011865
GGT2	0.793666092	0.65111851	0.967421224	0.022144922	0.029541801
S1PR4	0.825832213	0.700941171	0.972975867	0.022170261	0.04691797
ACKR1	0.904163991	0.829384341	0.985685988	0.022178514	0.027199087
RTN1	0.831356771	0.709637589	0.973953595	0.022212306	0.005927594
ARCN1	1.347116735	1.043479499	1.739107953	0.022220801	0.006957183
UBA6	1.332918634	1.041832947	1.705332981	0.022256475	0.006484787

KSR2	0.711706082	0.531676321	0.952695329	0.022274694	0.025822637
EMC7	1.453638873	1.054496183	2.003863084	0.022374464	0.011591173
GPR18	0.802463059	0.664338603	0.969305348	0.022404332	0.011785688
GPIHBP1	0.87012923	0.7721749	0.980509566	0.022431687	0.033406587
CBLC	1.157576197	1.020899373	1.312551154	0.022452788	0.004218029
DUSP2	0.842416411	0.727048274	0.976091182	0.022489447	0.031026727
IGSF6	0.859493001	0.754672682	0.978872346	0.022503142	0.00549893
FANCA	1.214511549	1.027577291	1.435452414	0.022668642	0.014350286
FEZ2	1.405405991	1.048786607	1.883286825	0.022670864	0.030935006
CDK7	1.375076337	1.045571632	1.808422181	0.022679031	0.010352296
TWIST1	1.128419876	1.017001181	1.252045169	0.022738854	0.041152956
FGF5	1.191343298	1.024655527	1.385147316	0.022805021	0.035708901
MPZL1	1.416198465	1.049588768	1.910860858	0.022809703	0.022229048
HGSNAT	1.229825058	1.029174405	1.469595111	0.022821894	0.00945993
P4HA2	1.257100072	1.032260176	1.530913067	0.022860207	0.004348871
COPS7A	1.431049112	1.050807661	1.948883355	0.022937898	0.008595655
TIFAB	0.702326055	0.517928147	0.952375133	0.022966827	0.037640651
PRAM1	0.836552826	0.717270924	0.975671266	0.022980449	0.030062456
TRIAP1	1.403977192	1.047761261	1.881298756	0.023060678	0.008368228
RAB30	0.760640703	0.600733858	0.963112485	0.023080871	0.034697768
CHRAC1	1.335564885	1.04051835	1.714274008	0.023097981	0.003841353
OPHN1	0.819467413	0.690129198	0.97304511	0.023102177	0.014473142
PYHIN1	0.80723579	0.671022517	0.971099486	0.023148592	0.024856464
TMC7	1.214746024	1.026956962	1.436874141	0.023182597	0.016059566
ZNF691	0.702296907	0.517413806	0.953242723	0.023380092	0.027192512
IFI35	1.205968673	1.025695039	1.417926757	0.023385626	0.017592198
SMG7	1.352609442	1.041669046	1.756366197	0.023434036	0.008830701
PSMC3	1.457394867	1.052043143	2.018928418	0.023508759	0.003360113
MLF2	1.315469169	1.037513866	1.667890126	0.023570894	0.037713317
QRFP	1.407159966	1.046938421	1.891323433	0.023574259	0.014590429
IMMT	1.406117492	1.046708614	1.88893678	0.023630928	0.001196371
LSAMP	0.828714411	0.704267583	0.975151478	0.023632533	0.033520813
GCN1	1.280992719	1.033715743	1.587421259	0.023636188	0.047582484
CLIC5	0.893247329	0.810032952	0.985010288	0.023655709	0.0438781
CHAD	0.878058707	0.784505725	0.982767962	0.023674759	0.028105325
FAM98A	1.41286403	1.047194523	1.906221551	0.023713474	0.006618759
CYTIP	0.846530669	0.732696496	0.978050499	0.023748404	0.022888719
PRCD	0.541214575	0.31782386	0.921621227	0.023791022	0.015165784
PCDH15	0.415110732	0.193642662	0.889870639	0.023829368	0.01189311
FAM47E	0.687903122	0.497285294	0.951587972	0.023839878	0.03603364
CLEC9A	0.688216608	0.497669001	0.95172112	0.023874308	0.023593569
C19orf84	1.207418417	1.024966646	1.422347975	0.024133814	0.003787856
XPNPEP3	1.393018163	1.044311544	1.858161592	0.02413865	0.037101526

CLCN6	0.79763921	0.655298822	0.970897991	0.024167977	0.009056378
NDEL1	1.452660613	1.049976144	2.009781716	0.024171041	0.006946081
IKZF5	0.71446222	0.533341718	0.957090449	0.024197841	0.01021949
HPS3	1.316912776	1.036535948	1.673129872	0.024212449	0.037916014
P2RY10	0.851681654	0.740694509	0.979299335	0.024221528	0.042588499
TXNDC9	1.365409875	1.041404121	1.790221576	0.024227373	0.022537076
SLC20A1	1.222069699	1.026432734	1.454994857	0.024254702	0.005394354
SAMD4B	1.297314331	1.034371319	1.627098937	0.024297783	0.002798057
GPR15	0.853145844	0.742907186	0.979742619	0.024457116	0.032278862
POC1B	1.277800672	1.032040698	1.582083497	0.024490227	0.014417313
NDRG1	1.165322133	1.019824006	1.331578454	0.024547714	0.016048708
SLC26A5	0.756006799	0.592370406	0.964846106	0.024606705	0.005354662
RFFL	1.331265425	1.037271705	1.708585727	0.024615218	0.015074947
CADM1	0.886452752	0.797991487	0.984720381	0.024638455	0.022541035
DONSON	1.233134379	1.027126689	1.480460407	0.024643332	0.002455612
SCCPDH	1.231956422	1.026924953	1.477923601	0.02470206	0.040685938
MRPL21	1.298079556	1.033784446	1.629943785	0.024703029	0.000967804
PRDX6	1.332792437	1.037203876	1.712619594	0.02473681	0.019718014
COL21A1	0.871512892	0.772865469	0.982751528	0.024842408	0.044505727
DPP3	1.319449882	1.035676055	1.68097735	0.024852847	0.032427462
MRPL28	1.379325135	1.041303963	1.827072495	0.024951963	0.019917895
KCNJ15	0.902914132	0.825774424	0.987259846	0.025002498	0.048399881
FAM89A	1.176470162	1.020588105	1.356161251	0.025027968	0.01235646
MS4A7	0.86616944	0.763880316	0.982155821	0.025040451	0.043224096
ZNF281	1.26213765	1.02953321	1.547294865	0.025090275	0.014486287
ITGB7	0.823326768	0.694477751	0.976081617	0.025171625	0.024266578
ARPC3	1.41701515	1.044349747	1.922662346	0.025176946	0.036708766
TPSAB1	0.901235098	0.822781179	0.987169763	0.025230261	0.022899795
NLRX1	1.325494385	1.035600552	1.696537685	0.025236363	0.006270149
RPS19	1.256922612	1.028782668	1.535654227	0.025244339	0.014677519
FAM177B	1.110322706	1.013003735	1.216991083	0.025350656	0.009908598
ABCD2	0.71266965	0.529565889	0.959083734	0.025371753	0.030142407
ZNF581	1.305116852	1.033377898	1.648312781	0.025377559	0.010216105
SLC25A47	1.867289332	1.079945506	3.22865314	0.025400747	0.02271961
KANK1	0.793831368	0.648295635	0.972038383	0.02545527	0.024729247
CTD-2192J16-20	0.497755441	0.269884071	0.918025576	0.025494486	0.031193356
CTB-50L17-14	0.738654929	0.566244615	0.963560782	0.02550456	0.049693713
DCLRE1B	1.324594781	1.034950946	1.695299028	0.025559549	0.019054888
METAP1D	1.292571039	1.031812337	1.61922845	0.02558986	0.032616549
GINS4	1.183400124	1.020651773	1.372099564	0.025696996	0.005749049
VDAC3	1.26553629	1.028998765	1.556447058	0.025697766	0.002970372
PITPNB	1.40496972	1.042021169	1.894337631	0.025752921	0.045811188
CA9	1.067909647	1.007966565	1.131417502	0.025801694	0.000605035

ZNF90	0.87063205	0.770781846	0.983417255	0.025812969	0.017700886
PSTPIP1	0.825567484	0.697509797	0.977135625	0.025819276	0.001165365
MTERF3	1.32307989	1.034344653	1.692414991	0.025827683	0.021283506
IKZF1	0.843195798	0.725727765	0.979677487	0.025867271	0.005046545
GCSAM	0.761664087	0.599442358	0.967786433	0.025885925	0.00811017
MOV10	1.280633357	1.030106997	1.592088782	0.025945385	0.017341921
CSF2RB	0.859759262	0.75268804	0.982061558	0.025966546	0.000998177
FAM83G	1.240425949	1.026087377	1.499537532	0.02601163	0.008267219
STMP1	1.389167755	1.039877608	1.855782869	0.026107767	0.001403124
H4C9	1.170496336	1.01887544	1.34468024	0.026138223	0.00503795
TNIK	0.865556925	0.762112744	0.98304194	0.026192834	0.013460253
ATP6V1D	1.451498004	1.04506458	2.015996424	0.026219759	0.027379701
RNF121	1.401010494	1.040629082	1.886195993	0.026252603	8.17E-05
CHRNA5	1.124991753	1.013985055	1.248150986	0.026284588	0.002962242
RNF217	1.23993232	1.025682537	1.498935686	0.026285631	0.000628636
C3orf33	1.335261037	1.034606837	1.723284608	0.026327284	0.012704672
C14orf39	0.669315937	0.469569653	0.954030611	0.026405763	0.007719004
APOBEC3H	0.833843149	0.710233557	0.978965849	0.026442985	0.037580524
PHLDA2	1.147702253	1.016216883	1.296200135	0.026480406	0.02800675
PAPLN	0.847417518	0.732123121	0.980868422	0.026497024	0.022708056
RASAL2	1.252468766	1.026387557	1.528348623	0.026664449	0.006184043
FAM210A	1.317203734	1.032326736	1.680694316	0.026702249	0.011225288
MRPL9	1.339315065	1.034243623	1.734373608	0.026741761	0.010662421
HOXA11	1.150783427	1.016282109	1.303085515	0.02678415	0.026273537
CKAP5	1.254606387	1.026367696	1.533599695	0.026828515	0.044435781
AVPR2	0.752448112	0.584946425	0.967914558	0.026842979	0.00888574
NCS1	1.200896358	1.021200673	1.412212213	0.026853521	0.003845632
LIMD1	0.805457678	0.664930691	0.975683752	0.026993468	0.035044992
MDH1	1.458364595	1.043852869	2.037478032	0.027000013	0.002770929
ZNF704	0.852073993	0.739298897	0.982052176	0.027105791	0.030732833
KMT5A	1.337142738	1.03338279	1.730192063	0.027124814	0.008105039
NDUFB5	1.320410709	1.031633554	1.690023007	0.027293724	0.027362369
YEATS4	1.164684433	1.017156901	1.333609227	0.027374242	0.006559947
DNAJB1	1.265751162	1.026508574	1.560752676	0.027473024	0.03775193
SHISAL2A	0.746266614	0.575270263	0.968090818	0.027510362	0.027495317
DHX33	1.298758458	1.029213137	1.638896231	0.02762425	0.001253566
NDUFAB1	1.376428593	1.035805807	1.829064541	0.027631277	0.018040264
CYRIA	0.833888023	0.709395433	0.980227955	0.027662299	0.031367335
MRM3	1.374330088	1.035539442	1.823960646	0.027680285	0.01290482
UBE2N	1.401856945	1.037776464	1.893666857	0.027690725	0.003920495
PRELP	0.889396027	0.801213991	0.987283424	0.027792893	0.042586738
LTB	0.8849248	0.793587129	0.986774952	0.027843269	0.021087997
RHOBTB2	0.878156644	0.782130585	0.9859723	0.027873444	0.031382156

UBXN11	0.83093166	0.70437082	0.980232859	0.028036668	0.027308599
GIMAP8	0.836669659	0.713592487	0.98097462	0.028051062	0.002379163
H2BC5	1.152659636	1.015350773	1.30853718	0.028137082	0.037236994
PTCH1	0.799132729	0.65401106	0.976456146	0.02830884	0.01200902
RTN3	1.350300603	1.032376011	1.766131429	0.028337345	0.023549145
CBX1	1.273843362	1.02580193	1.581861823	0.028486768	0.022791877
ZNF69	0.814485893	0.677851814	0.9786612	0.02851049	0.042840788
FBXO22	1.420735874	1.037300094	1.945907878	0.028658764	0.034649861
UBE2D3	1.554149497	1.047003622	2.306945848	0.028678362	0.030233117
ZNF80	0.564478454	0.338182148	0.942202085	0.028689431	0.040613076
CEP152	1.277124823	1.025462825	1.590547969	0.028924484	0.000855549
CLDN12	1.257616011	1.023745617	1.544913115	0.028997546	0.02023886
SEC14L2	1.187181515	1.017700737	1.38488644	0.029019685	0.007812148
TWSG1	1.24592581	1.022738812	1.517817752	0.029020157	0.023162402
FAM136A	1.313576877	1.028268158	1.678048862	0.029029992	0.005894897
SAMD3	0.674910879	0.474123148	0.960730764	0.029085645	0.010007068
STEAP2	1.132501683	1.012692064	1.266485744	0.029180842	0.000250471
SUOX	0.766386386	0.603381291	0.973427751	0.029205944	0.025629599
DDX60L	1.199785788	1.018559651	1.413256393	0.029252423	0.016827195
PTPRN	1.131795179	1.0125656	1.265064037	0.029270271	0.015865424
GIMAP6	0.838192798	0.715157842	0.982394551	0.029311756	0.001980145
ORC2	1.404156392	1.034378175	1.906126039	0.029501668	0.008365616
LCN10	0.426093789	0.197643455	0.918603234	0.02951196	0.007437807
MFNG	0.811730537	0.672709069	0.979482061	0.029535698	0.002736198
CXCR5	0.592983086	0.370345295	0.949462423	0.02956356	0.010898517
HMBS	1.276626531	1.024494451	1.590809301	0.02959026	0.047283776
C1orf131	1.425921264	1.035775317	1.963023657	0.029594242	0.040520136
CDK15	0.625643616	0.410017169	0.954667178	0.029619954	0.015452733
SMIM35	0.645488429	0.435052586	0.957712527	0.02965957	0.027416669
HCLS1	0.861041078	0.752411668	0.985353856	0.029675295	0.01389927
PCBP1	1.440239406	1.036609309	2.001033107	0.029685943	0.012347176
RCE1	1.334019791	1.028796433	1.729796824	0.029695159	0.000370934
SNUPN	1.453331562	1.037363486	2.036096949	0.029765703	0.007038604
KAT2B	0.803557084	0.659666447	0.978834062	0.029820456	0.000913883
ATP1B3	1.262071201	1.022742458	1.55740451	0.030038726	0.038707735
UPK1A	1.183892653	1.01644292	1.378928209	0.030039036	0.005952957
PDF	1.297449884	1.025288358	1.64185635	0.030168162	0.010931626
TUBB	1.233836855	1.020341246	1.492004161	0.030181541	0.014921924
C8orf76	1.263596583	1.022594922	1.561396689	0.030242187	0.003963021
TOX	0.87053	0.767896619	0.986880866	0.030288922	0.003137857
PIK3CD	0.834111303	0.707797531	0.98296707	0.03038638	0.006562903
RILPL2	0.781057422	0.624487346	0.976882398	0.030397463	0.012217432
RORA	0.800535027	0.654424247	0.979267399	0.030485661	0.014276268

EBAG9	1.361961203	1.029486701	1.801808919	0.03050425	0.010261739
PRMT8	0.798376901	0.651000948	0.979116355	0.030567144	7.57E-05
FUBP1	1.338561167	1.027608527	1.743607561	0.030627574	0.007422752
LASP1	1.26941283	1.022523471	1.575913883	0.030632844	0.017478325
RSL1D1	1.352452587	1.028585637	1.778294323	0.030636018	0.019573072
TRIM62	0.762860206	0.596840952	0.975059925	0.030644248	0.040255908
UTP6	1.424909834	1.033413084	1.964720659	0.030734553	0.036309216
DEF6	0.805178191	0.661441518	0.980150023	0.030787998	0.020831225
MGMT	0.826014731	0.694334162	0.982668538	0.030983361	0.014333268
PTPN12	1.337222186	1.026790668	1.741507036	0.031073757	0.036294377
PSMD13	1.403904463	1.031325513	1.911082113	0.031084951	0.002227914
ARPC2	1.476235961	1.035948023	2.103650534	0.031130384	0.017227572
NPHP1	0.752178742	0.580551966	0.974543009	0.031153505	0.005973588
HNF4G	1.134960712	1.011520369	1.273465031	0.031166386	0.031078508
RECQL	1.22825315	1.018752635	1.48083622	0.031187094	0.015278095
FYCO1	0.818890578	0.682786004	0.982125842	0.031205738	0.049377917
TBCD	0.779149795	0.620899522	0.977733726	0.031212028	0.02749741
DIABLO	1.412993653	1.031415473	1.93573891	0.031353094	0.002699063
GLP2R	1.243992563	1.019671483	1.517662818	0.031399106	0.043404625
C4A	0.877492233	0.779002005	0.988434707	0.031439118	0.035429903
KCTD9	1.316807239	1.024753714	1.692095653	0.031469315	0.011664741
TMEM213	0.884067286	0.79014056	0.989159404	0.031542614	0.025661294
RHEX	0.845298118	0.725222715	0.985254451	0.031555	0.022874967
ATP8B4	0.752137506	0.580130172	0.975144639	0.031559711	0.000297166
NRBP1	1.383051801	1.02901331	1.858899459	0.031591725	0.047839051
DCAF13	1.284189618	1.022231788	1.613276944	0.031644963	0.042259353
HSD11B1L	0.77939267	0.620877588	0.978377937	0.031683195	0.044808236
HACD4	0.811503808	0.670688814	0.981883724	0.031715342	0.029792433
KNDC1	0.88409616	0.790032833	0.989358907	0.03184428	0.009878218
TTC9	1.158564056	1.012850403	1.325240793	0.031860962	0.032555772
LDHB	1.164309179	1.013283574	1.337844509	0.031862873	0.003140346
TNFRSF10C	0.858897962	0.747468325	0.986939092	0.031921054	0.007453777
RPL39L	1.12595468	1.010258523	1.254900516	0.031996105	0.009957507
CLDN23	0.830520711	0.700863452	0.984164104	0.032010267	0.040847333
RPL4	1.317383607	1.023826683	1.695110704	0.032110447	0.038829888
LPXN	0.818000424	0.680646202	0.983072691	0.032193909	0.005198341
VCP	1.389120997	1.028322383	1.876509911	0.032195267	0.017619808
NRIP2	0.757417582	0.58735788	0.976715242	0.032228685	0.017675818
METTL8	1.380739658	1.027695316	1.855065381	0.032251188	0.001778521
PSMC3IP	1.229035369	1.017570512	1.484445471	0.032292488	0.027234716
RSPH9	0.812883408	0.672368432	0.98276392	0.032393468	0.017121839
GALNT14	1.098670129	1.007926665	1.197583211	0.032397248	0.003818883
PLCB2	0.855420383	0.741366275	0.987020932	0.03244399	0.033936471

FAM214A	0.783598981	0.626583019	0.979961705	0.032567064	0.001357292
MTFP1	1.264430793	1.019665014	1.567951443	0.032567867	0.015102797
LST1	0.870438428	0.766348504	0.988666451	0.032730494	0.004866964
C16orf91	1.323438323	1.023272904	1.71165384	0.032739617	0.030770647
NAA20	1.311611023	1.022459043	1.682535342	0.032780717	0.004492029
CT83	1.058657649	1.00467303	1.115543051	0.032797979	0.006474274
NUP54	1.373783086	1.026221564	1.839057017	0.032851921	0.003570529
SLC5A7	0.527526296	0.293169621	0.949225203	0.032857392	0.022171823
BID	1.222386845	1.016422281	1.470087412	0.032925772	0.023935797
GPSM2	1.209784001	1.015488182	1.44125491	0.033004572	0.019197563
ITPKB	0.822381868	0.686970386	0.984484849	0.033146059	0.006117298
ANP32D	0.252098767	0.070953006	0.895716637	0.033153324	0.019122632
RPS17	1.299837769	1.021047755	1.654749464	0.033248199	0.008804811
IL7R	0.884156158	0.789350631	0.990348371	0.033373969	0.004476381
PPP2R1B	1.225605705	1.016039046	1.478397263	0.033480675	0.023793275
BIRC2	1.335193957	1.022833825	1.742944806	0.033502743	0.000760597
HSFX1	0.156272541	0.028226685	0.865178008	0.033517909	0.019843879
SEC11C	0.860540118	0.749253605	0.988355997	0.033525744	0.017604546
TMEM98	0.866310175	0.75888046	0.988948008	0.033629785	0.036509834
TCAF1	1.283679616	1.019363793	1.616531182	0.033753383	0.038119484
ACAT1	0.802905666	0.655609235	0.983295346	0.033764529	0.031443334
CRHBP	0.54647487	0.312654577	0.955158843	0.033922149	0.002121911
PARVG	0.841327958	0.717170167	0.986980168	0.03393571	0.045504458
ZNF182	0.760286526	0.590105873	0.979545583	0.034021512	0.048687725
CRY2	0.829660925	0.698082724	0.986039658	0.034045321	0.003106769
PDCL3	1.345014419	1.022336244	1.769538933	0.034192071	0.028814131
UBLCP1	1.340106154	1.022049125	1.757141079	0.034197647	0.021352984
C12orf45	1.326058639	1.021167755	1.721981042	0.034251036	0.011315312
KDELR2	1.298682256	1.01955034	1.654234749	0.03427833	0.030402563
SNRPB	1.2531344	1.016650306	1.544627306	0.03445364	0.034522414
BCL2L2-PABPN1	1.352121334	1.022247629	1.788443475	0.034500861	0.000726285
EIF2B1	1.407355204	1.025094391	1.932162236	0.034580304	0.011709297
ZNF630	0.745438289	0.567593661	0.979007133	0.034641365	0.042486665
EN1	1.322194766	1.020257853	1.713487424	0.034721409	0.034102348
ERG	0.817924844	0.678670822	0.985751897	0.034800815	0.045090833
TM4SF1	1.151480974	1.01011408	1.312632365	0.034811632	0.020877713
IL23R	0.385835567	0.159266807	0.934715071	0.034900464	0.009597102
BAG2	1.172076798	1.011309176	1.358401618	0.034913194	0.033036644
MPDU1	1.313841036	1.019445907	1.693251458	0.034965803	0.039093378
AIMP1	1.463126568	1.02696697	2.084526004	0.035091028	0.002882715
RPA3	1.257487114	1.016138514	1.556159736	0.035099618	0.015127512
CYLD	0.771274417	0.605623754	0.982233973	0.035267474	0.037422874
NOB1	1.341902739	1.0204812	1.764562602	0.035283702	0.023266855

MMP1	1.070549994	1.004688522	1.140728956	0.035347825	0.038861584
TMEM233	0.854808334	0.738561661	0.989351771	0.035421482	0.010571075
POLR2H	1.295084289	1.017784691	1.647935296	0.035434268	0.025689727
CRP	1.210481454	1.01307508	1.446354154	0.035467634	0.006975193
PRDM8	1.175479406	1.011013783	1.366699305	0.035518079	0.033790429
THSD1	0.799510859	0.64897172	0.984969906	0.035531531	0.043797511
SAP30BP	1.40180449	1.023078336	1.92072861	0.035557386	0.011716719
CYC1	1.229379188	1.014012501	1.490487726	0.035590561	0.003345525
CRYM	0.923989202	0.85827523	0.994734573	0.035709676	0.044582317
MCRS1	1.420586336	1.023700477	1.971343752	0.035721335	0.008232354
EFNA3	1.164243365	1.010182601	1.341799603	0.03574133	0.047231657
GIMAP5	0.771676551	0.605846055	0.98289771	0.035754459	0.016192022
LY86	0.877625162	0.776860386	0.991459905	0.035922941	0.00845572
UQCRQ	1.262639009	1.015379479	1.570109797	0.035974109	0.033028735
ERMN	0.726745737	0.539302348	0.979338154	0.035980874	0.028202437
DUSP14	1.247271607	1.01423665	1.533849582	0.036266468	0.009163802
RHBDF2	1.230663502	1.013299873	1.494653947	0.036330298	0.020200826
PBXIP1	0.832097025	0.700481491	0.98844219	0.036412178	0.042015599
NRDC	1.341689752	1.01873558	1.76702515	0.036431089	0.014741342
LYPLA1	1.194706015	1.011264535	1.411423434	0.03646834	0.036592954
ZNF384	1.282168896	1.015746181	1.618472319	0.036489857	0.032867728
TIMMDC1	1.373395023	1.019982755	1.84926057	0.036589554	0.032036085
INPP5A	0.771365881	0.604714565	0.98394409	0.036590943	0.042051794
SYT2	0.853863464	0.736196142	0.990337729	0.036770294	0.031147856
CDC7	1.166892019	1.009473374	1.348858741	0.03684409	0.026614516
UGGT2	1.32422799	1.017011128	1.72424836	0.037050018	0.014384167
C1GALT1	1.196583368	1.010830154	1.416471156	0.037058961	0.005175313
DMTN	0.863747435	0.752627525	0.991273381	0.037095528	0.037144758
PI3	1.065501582	1.003800558	1.130995208	0.037105605	0.005064607
CRHR2	0.805408124	0.657043576	0.987274315	0.037227967	0.004392469
TNFAIP8L2	0.863742445	0.752528317	0.991392609	0.037261537	0.004446406
NME8	0.680215076	0.473324899	0.97753689	0.037273166	0.038044743
C3orf62	0.760110609	0.587080331	0.984138127	0.037407684	0.009574852
RBPMS	0.855704789	0.738890601	0.990986601	0.037445817	0.005617995
CCNE2	1.175907112	1.009448502	1.369814838	0.037459481	0.005930198
RASGRP4	0.804852304	0.655997071	0.987484944	0.037464496	0.0099713
SELP	0.8726467	0.767538287	0.992148895	0.037494888	0.003855432
MRPS18C	1.522759198	1.024604099	2.263113702	0.037507232	0.032208865
HOXC8	1.146271332	1.007838473	1.303718803	0.037629833	0.020194048
ELMO1	0.831549222	0.698688047	0.989675022	0.037818829	0.001951193
FAM78A	0.826032254	0.689701657	0.989310781	0.037825654	0.011289109
CSDE1	1.326020937	1.015938275	1.730746413	0.037864936	0.025902875
CPSF3	1.345829141	1.016685203	1.781530871	0.037930639	0.017548915

UTP15	1.371340681	1.017498097	1.848234674	0.038089926	0.030328058
C15orf48	1.088609087	1.004664809	1.179567288	0.038113388	0.038820211
BCAS2	1.357536624	1.016779333	1.812493257	0.038190623	0.018239273
COQ2	1.347282883	1.016351216	1.785968411	0.038196375	0.012264109
RIF1	1.289523037	1.013901143	1.640070805	0.038220036	0.036988282
DLEC1	0.855986702	0.738912194	0.991610693	0.038243685	0.010542285
CFAP221	0.888608137	0.794659644	0.993663674	0.038316125	0.015943035
MYRIP	0.827204158	0.691221846	0.989937925	0.0384212	0.013540996
NSUN2	1.232317418	1.011168373	1.501833186	0.038450332	0.036596163
MTFR1L	0.710304348	0.513740589	0.982075931	0.038510117	0.041013779
CD180	0.848821632	0.726756788	0.99138828	0.038532108	0.030530062
FKBP10	1.114169515	1.005630431	1.234423373	0.038703025	0.007079909
NANOS1	0.827102008	0.690859979	0.990211841	0.038727117	0.007122979
GPR19	1.22086451	1.010302646	1.475310549	0.038820548	0.04299772
PLBD2	1.263874466	1.012027943	1.578393834	0.038883857	0.025583445
HOMER1	1.214698605	1.00994843	1.460958457	0.038916384	0.038720879
MAN1C1	0.835150973	0.703889523	0.990890082	0.038935888	0.010786585
FFAR4	0.840233389	0.71225761	0.991203377	0.03894523	0.041986525
WDR5	1.330774361	1.014586228	1.745500135	0.038962149	0.034976842
AKT2	1.332609458	1.014505669	1.750456426	0.039069255	0.006709093
SBK1	0.900352749	0.814895405	0.994771927	0.03911429	0.031206909
C11orf86	1.079263459	1.003779927	1.160423299	0.039212558	0.005608879
DLX1	1.32604877	1.013991496	1.734142098	0.039259018	0.008675135
PHF23	1.41504276	1.017138889	1.968606288	0.039317974	0.017258573
F5	1.095223239	1.004460932	1.194186758	0.039321263	0.015321647
UHMK1	1.303067585	1.012869366	1.676410787	0.039451657	0.042723791
GPLD1	0.765738163	0.593931255	0.987243776	0.039494304	0.042537193
C10orf143	0.771102649	0.602074161	0.987584809	0.039501951	0.047951087
GNAO1	0.817665946	0.675070654	0.990381665	0.039512278	0.0156894
FHL1	0.888407714	0.793680032	0.994441381	0.039699674	0.031176321
FXYD5	1.168184384	1.007322275	1.354735013	0.039736425	0.015688976
TIMM23	1.361574372	1.01410097	1.828106691	0.040061948	0.034826755
WASF3	0.855363824	0.736803753	0.993001554	0.040148393	0.035665839
IFT57	0.873903313	0.768283599	0.994043086	0.040279176	0.021391555
ADH1C	0.930909031	0.869327934	0.996852384	0.040340518	0.010159604
ACP1	1.376578626	1.014115007	1.868593501	0.040377897	0.027432204
TNFSF12	0.81607777	0.671883614	0.991217695	0.040473057	0.007258726
GEMIN7	1.276863047	1.010597823	1.613281965	0.040529181	0.025503633
CD33	0.832445376	0.698407808	0.99220727	0.040628529	0.003262415
H2AZ2	1.329034588	1.012168812	1.745097178	0.040657516	0.034824384
CD83	0.862428706	0.74841381	0.993812865	0.040780919	0.041606078
CHMP5	1.322837106	1.011715711	1.729634115	0.040844527	0.003276733
ADAR	1.281797469	1.010284673	1.626279003	0.040932202	0.03679912

ATG16L1	1.384566409	1.01323589	1.891982073	0.041101703	0.045294319
GLOD4	1.405197775	1.013760254	1.947778857	0.041151525	0.047389741
GPSM3	0.847478153	0.722995938	0.993393161	0.041176657	0.038751423
ATRIP	0.266207867	0.074703712	0.948635977	0.041221672	0.049012181
MPEG1	0.877780995	0.774504773	0.994828568	0.041236081	0.002103441
SLC46A2	0.898440419	0.810616587	0.995779262	0.041295089	0.02953928
AFF3	0.853081632	0.732288813	0.99379952	0.041369488	0.038360394
SCIMP	0.86059501	0.744982694	0.994148961	0.041381219	0.002909018
CD99L2	0.825536641	0.686539049	0.992675866	0.041538911	0.001006574
ZNF596	0.733845819	0.544921376	0.988270436	0.041584411	0.030637111
KNTC1	1.176453598	1.006206551	1.375505921	0.041595598	0.016481175
PATL1	1.258288812	1.008748447	1.569559526	0.041630165	0.038596953
TM9SF1	1.381534198	1.012273218	1.885495639	0.041665916	0.031700689
CHCHD5	1.289516823	1.009561466	1.647104899	0.041733466	0.042569632
ENTPD1	0.782467522	0.617863221	0.990923885	0.041788001	0.027674522
RCC2	1.242186057	1.008059746	1.530689234	0.041820736	0.042026465
NBPF1	0.775845514	0.607476998	0.990879101	0.042014877	0.02973334
PCDHAC1	1.158496159	1.005286756	1.33505524	0.042070757	0.041730079
TMEM223	1.284963627	1.008969529	1.636453307	0.042118831	0.003622146
SLC38A1	1.149234179	1.004933966	1.31425471	0.042167798	0.00848616
TSR3	1.352428752	1.010710888	1.809680246	0.04218809	0.024280616
ST8SIA1	0.749894212	0.568052467	0.989946108	0.042226882	0.006474872
CCDC189	0.799465657	0.644109175	0.992293484	0.042342457	0.007475413
PFN2	1.1207022	1.003918703	1.251070845	0.042394947	0.032210666
SEPTIN7	1.377854527	1.010676827	1.878427451	0.04264911	0.015488038
GS1-114I9-3	0.632834159	0.406567915	0.985023799	0.042682237	0.002111092
RNF2	1.318369768	1.009090841	1.722440412	0.042733078	0.03709151
CD84	0.87347074	0.766134954	0.995844309	0.043154261	0.021666848
NPIPB7	0.299056375	0.092804383	0.96369064	0.043185318	0.020353138
PUF60	1.289763812	1.007734324	1.65072346	0.043263087	0.006351322
WASHC2C	0.747344755	0.563455412	0.991248234	0.043283528	0.002854462
NABP2	1.307164005	1.007725368	1.695578766	0.043597523	0.020167554
VAMP8	0.795558802	0.637080985	0.99345895	0.043605445	0.041263813
SLC14A2	0.906741779	0.82437692	0.997335848	0.043918362	0.038011429
EIPR1	1.339282328	1.007724056	1.779928883	0.044116417	0.029437071
ADAMTS17	0.770806497	0.598212521	0.993196624	0.044141293	0.029335088
SENP8	0.721674988	0.525276422	0.991506122	0.044157073	0.028286511
AHCY	1.255408782	1.005909611	1.566792077	0.044210914	0.003054443
SHISA4	0.833955141	0.698697876	0.995396153	0.044317808	0.011623572
UEVLD	1.371410275	1.00794874	1.865934316	0.04439324	0.013610991
DOCK8	0.850786533	0.726763494	0.995974249	0.04441603	0.036658821
CLEC4A	0.868610863	0.757118174	0.996521886	0.044465778	0.001710111
IMPA1	1.258486596	1.005601798	1.574965871	0.044561371	0.035324192

WDR37	0.760453026	0.582100835	0.993451256	0.044627828	0.014822922
FOXK2	1.303710692	1.00633486	1.68896223	0.04466775	0.003063907
HSPB7	0.85199203	0.728600663	0.996280207	0.044786833	0.024764251
FGF14	0.757130988	0.57688183	0.993699755	0.044907214	0.037798155
NAMPT	1.13891635	1.002944166	1.293322696	0.044932599	0.024628654
EVI2B	0.8696746	0.758677885	0.996910447	0.045029258	0.003629844
M1AP	0.768642566	0.594219539	0.994264502	0.045095095	0.007189413
CAMK1	0.795530937	0.636009928	0.995062253	0.045143482	0.011904316
ID2	0.839302257	0.707086099	0.996241165	0.04517485	0.010350874
RIC8A	1.417258633	1.007218021	1.994227657	0.045366799	0.034908872
ETFB	1.275053798	1.004908321	1.6178214	0.04547109	0.00984333
CDK20	0.779914129	0.611294954	0.995045099	0.045507967	0.034505447
JUP	1.187798408	1.003433372	1.406037608	0.045522851	0.014445212
PPP1R16B	0.866647508	0.753187579	0.997199003	0.045592857	0.008417239
SYP	0.829227997	0.690106381	0.99639576	0.045662583	0.018475624
ZNF442	0.670544673	0.45294617	0.992679017	0.045860697	0.011408466
ITIH4	0.720817452	0.522713931	0.99400029	0.045861349	0.041198375
ZHX1-C8orf76	1.288884762	1.00467549	1.653493038	0.045861697	0.011144119
TNPO3	1.285401206	1.004555749	1.644763133	0.045922873	0.008698671
HNRNPA1	1.371316257	1.005674025	1.869898426	0.045964042	0.02715934
MYO1F	0.848760558	0.722445972	0.99716036	0.046091636	0.014105245
KHSRP	1.288424228	1.004383708	1.652791636	0.046109679	0.002718767
GOLGA8B	0.886074127	0.786699106	0.998002098	0.046270706	0.048661324
CKMT2	0.824163779	0.681242628	0.997069042	0.046572068	0.026050542
OMG	0.876280521	0.769387101	0.998024987	0.046618602	0.039082328
FCRL4	0.719880483	0.520744771	0.995166804	0.046669423	0.015593534
TRIB3	1.15899789	1.002125445	1.340427106	0.046747828	0.023748286
AGO4	0.797800388	0.638497601	0.99684863	0.04684071	0.038342205
SLC11A2	0.790614645	0.627079192	0.99679837	0.046912892	0.012218984
LCN6	0.631978474	0.401715486	0.994228018	0.04714409	0.042362173
SPATA1	0.641895063	0.414249695	0.994639892	0.047254017	0.048645697
ATG101	1.323327953	1.003212165	1.745589751	0.047405364	0.047132609
RNF168	1.238098817	1.002425577	1.529179539	0.047428756	0.016609895
GPR34	0.88229624	0.779534221	0.998604852	0.047472371	0.007444068
PMEL	1.207952471	1.001997058	1.456240973	0.047604497	0.003724443
IDO2	0.687625985	0.474679191	0.996103272	0.047634775	0.013416384
PAIP2B	0.842712512	0.711386314	0.998282317	0.04771999	0.018120649
STOML2	1.283826892	1.00232466	1.644388843	0.04788924	0.031579139
MTFR1	1.19347179	1.001642646	1.422039006	0.047892329	0.038005174
BPIFB1	0.95266022	0.907957994	0.999563306	0.047953882	0.035113843
TCEANC	0.6887445	0.475855671	0.996875767	0.048092459	0.018021368
MEP1A	0.745559554	0.557202923	0.997588177	0.048130272	0.016074014
HEBP2	1.279641187	1.001905687	1.634366975	0.048243769	0.028284463

TTC39B	0.809929657	0.656952728	0.99852854	0.048410051	0.022496781
KCTD12	0.878849362	0.773037654	0.999144347	0.048490739	0.013355518
KLC4	0.799936904	0.640791705	0.998606951	0.048577556	0.013559841
GNG4	1.082037123	1.000480746	1.170241746	0.048611328	0.005343777
ATP5F1C	1.309598133	1.001639226	1.712240521	0.048616612	0.005286593
CDHR3	0.907262737	0.823596775	0.999427995	0.048660443	0.028989145
TMEM230	1.352337274	1.001719057	1.825677659	0.048703183	0.013092567
NUDT19	1.228783356	1.000973359	1.508440283	0.048922874	0.035933347
CSTF3	1.346886861	1.001405679	1.811557747	0.04892406	0.040975718
PLEKHG4B	0.896984256	0.804985708	0.999496945	0.048944181	0.042614484
ATP1A4	0.244737045	0.060256137	0.994026899	0.049028724	0.013284112
UQCC3	1.235620114	1.000846767	1.525465352	0.049086863	0.030977164
MECOM	0.88875984	0.790170672	0.999649925	0.049321653	0.002993305
APOBEC1	1.095091481	1.000261332	1.198912038	0.049342737	0.018683463
POLE4	1.253654797	1.000621905	1.570673542	0.049371536	0.030373954
ANO9	0.879011019	0.772891059	0.999701527	0.049470801	0.047083055
DOCK2	0.862875114	0.744796111	0.999674207	0.049494871	0.004665632
BICD1	1.230426994	1.000419383	1.513315928	0.049537612	0.036741535
EMG1	1.288382748	1.00017106	1.659646206	0.049845445	0.04610771

Supplementary Table S6: In-degree, out-degree and difference between in-degree and out-degree of GEO-HNS														
**CN**	**degree-out**	**degree-in**	**difference value**		**CM**	**degree-out**	**degree-in**	**difference value**		**DN**	**degree-out**	**degree-in**	**difference value**
ACTG2	0	17	-17		BTK	8	39	-31		ABCC9	5	5	0
AK1	14	12	2		CD19	18	3	15		ACE	1	0	1
AKT3	5	39	-34		CD247	24	37	-13		ACE2	1	0	1
ALDH1A1	11	3	8		EGF	25	12	13		ACHE	9	3	6
ALDH1A2	11	3	8		FCGR3A	16	29	-13		ACTL6A	2	0	2
ALDH2	16	19	-3		FGF10	12	26	-14		ADAM12	2	0	2
AOC3	7	9	-2		FGF2	13	26	-13		ADAM15	1	0	1
AOX1	10	7	3		FGFR4	12	27	-15		ADCY3	9	9	0
ATP2B1	29	7	22		FYN	40	36	4		AMY1A	1	0	1
ATP2B4	29	7	22		GAB1	29	36	-7		ANGPT1	3	1	2
BTK	8	39	-31		GAB2	27	32	-5		ANO1	3	2	1
CACNA1D	49	27	22		GNG11	24	1	23		ANO6	3	2	1
CACNA2D2	49	27	22		GNG2	37	10	27		AOC1	3	1	2
CALM1	27	47	-20		GUSB	15	27	-12		ARHGAP18	4	0	4
CATSPERB	29	7	22		HBEGF	19	4	15		ARHGAP24	4	0	4
CATSPERD	29	7	22		HLA-E	18	1	17		ARHGAP25	4	0	4
CD19	18	3	15		INPP5A	3	39	-36		ARHGAP29	4	0	4

CD247	24	37	-13		INPP5K	3	39	-36		ARHGAP31	4	0	4
CD79A	10	5	5		IRS2	19	3	16		ARHGAP44	4	0	4
CES1	2	5	-3		ITK	6	34	-28		ARHGAP6	4	0	4
CLIC2	29	0	29		KDR	20	5	15		ARHGAP8	4	0	4
CNGA1	36	31	5		KIT	22	2	20		ARHGEF16	4	0	4
CSF2RB	5	11	-6		KL	15	25	-10		ARHGEF3	4	0	4
CYP1B1	21	18	3		KLB	15	23	-8		ASPA	6	0	6
CYP2C18	23	18	5		KLRD1	18	1	17		ASS1	1	1	0
CYP2J2	23	17	6		LRRFIP 1	17	28	-11		ATP2B1	29	7	22
CYP3A5	20	15	5		NRG3	18	2	16		ATP2B4	29	7	22
CYP3A7	20	15	5		PDGFB	29	1	28		AURKB	10	8	2
DES	0	18	-18		PDGFR B	28	4	24		AXIN2	1	0	1
EGF	25	12	13		PIK3R1	30	34	-4		B3GAT1	9	0	9
EPHX1	9	7	2		PIP5K1 B	60	16	44		B4GALT7	8	0	8
FCGR3A	16	29	-13		TREM2	18	1	17		BCAT1	1	1	0
FGF10	12	26	-14		TYROB P	18	2	16		BIRC5	3	9	-6
FGF2	13	26	-13		WASF3	0	31	-31		BORA	1	4	-3
FGFR4	12	27	-15							BPNT1	12	5	7
FKBP1B	29	0	29							BRCA1	5	1	4
FYN	40	36	4							BST1	1	0	1
GAB1	29	36	-7							BUB1	4	4	0

GAB2	27	32	-5							CACNA1D	49	27	22
GNAI1	49	29	20							CASP1	3	0	3
GNAI2	53	30	23							CATSPERB	29	7	22
GNG11	24	1	23							CATSPERD	29	7	22
GNG2	37	10	27							CAV1	2	3	-1
GUSB	15	27	-12							CCNA2	10	6	4
HBEGF	19	4	15							CD274	2	0	2
HCK	11	7	4							CD38	1	0	1
HLA-E	18	1	17							CDC45	3	0	3
HSD17B2	10	5	5							CDC7	6	0	6
HSD17B6	10	5	5							CDK5	3	1	2
HSD17B7	11	5	6							CDKN2C	1	0	1
HTR3A	51	27	24							CEBPD	3	2	1
INPP5A	3	39	-36							CENPE	1	5	-4
INPP5K	3	39	-36							CEP70	1	0	1
IRS2	19	3	16							CEP72	1	0	1
ITGA1	2	18	-16							CERS6	1	0	1
ITK	6	34	-28							CETN2	1	0	1
ITPR1	29	13	16							CFLAR	1	1	0
JAK2	27	21	6							CHEK2	1	1	0
KDR	20	5	15							CHN1	4	0	4
KIT	22	2	20							CHRM3	1	0	1
KL	15	25	-10							CLIC2	29	0	29
KLB	15	23	-8							COLGALT2	21	0	21
KLRD1	18	1	17							CPA3	1	0	1

LMOD1	0	17	-17							CPB2	1	0	1
LRRFIP1	17	28	-11							CRTAP	22	0	22
MAOA	6	10	-4							CTPS2	5	2	3
MCOLN2	29	7	22							CTSE	1	0	1
MCOLN3	29	7	22							CTSK	4	0	4
MYBPC2	0	17	-17							CXCL11	3	0	3
MYH11	0	17	-17							CXCL13	3	0	3
MYL4	0	17	-17							DBF4	6	0	6
MYL9	0	19	-19							DCC	2	3	-1
NRG3	18	2	16							DEPDC1B	4	0	4
P2RX1	29	7	22							DEPDC7	4	0	4
PDGFB	29	1	28							DGAT2	1	0	1
PDGFRB	28	4	24							DLC1	4	0	4
PIK3R1	30	34	-4							DLG3	8	7	1
PIP5K1B	60	16	44							DPAGT1	1	0	1
PKM	20	12	8							DYNC1I2	2	0	2
PLA2G4A	6	32	-26							E2F7	2	0	2
PLN	0	21	-21							ECT2	4	1	3
PRKCB	4	25	-21							EDNRA	8	0	8
PRKCZ	1	23	-22							EFNA1	10	6	4
PXN	0	29	-29							EFNA3	1	0	1
RASGRP1	1	18	-17							EFNA4	1	0	1
RASGRP2	1	25	-24							EGLN3	2	0	2
RYR2	29	0	29							ENPEP	1	0	1
SLC24A3	31	28	3							EPHB2	8	6	2

SORBS3	0	17	-17							EPHB3	3	3	0
STX1A	25	24	1							EPHB6	3	3	0
TREM2	18	1	17							ESPL1	30	0	30
TRPC6	29	9	20							ETS1	4	1	3
TRPV2	29	7	22							ETS2	1	0	1
TYROBP	18	2	16							FAM13B	4	0	4
UGT2B15	2	14	-12							FEN1	5	0	5
UGT2B28	2	14	-12							FGD3	4	0	4
UNC13B	5	17	-12							FGD4	4	0	4
VAMP2	25	25	0							FKBP1B	29	0	29
VIM	0	20	-20							FLT4	1	0	1
WASF3	0	31	-31							FOSB	2	0	2
										FOSL2	4	2	2
										FOXA1	6	0	6
										FOXA3	2	0	2
										FUT8	1	0	1
										GALNT10	8	0	8
										GALNT12	8	0	8
										GALNT13	8	0	8
										GALNT14	8	0	8
										GALNT18	8	0	8
										GALNT2	8	0	8
										GALNT3	8	0	8
										GALNT6	8	0	8
										GALNT7	8	0	8

										GATA2	1	0	1
										GHR	2	1	1
										GMDS	3	1	2
										GNG11	24	1	23
										GRB14	2	0	2
										GRIA1	22	14	8
										GZMH	2	0	2
										HDAC1	3	2	1
										HGD	1	0	1
										HIF3A	2	1	1
										HLA-E	18	1	17
										HPSE	4	0	4
										HSD17B2	10	5	5
										HSD17B6	10	5	5
										HYAL1	1	0	1
										HYAL2	1	0	1
										IL1B	2	3	-1
										IL3RA	4	0	4
										IL6	4	5	-1
										IRAK3	9	0	9
										JUNB	8	0	8
										JUND	8	0	8
										KAT2A	4	0	4
										KAT2B	3	0	3
										KCNA5	5	5	0

										KCNJ8	5	5	0
										KCNK1	5	5	0
										KCNK17	5	5	0
										KIF11	1	0	1
										KIF15	1	0	1
										KIF18A	1	4	-3
										KIF20A	6	1	5
										KIF22	1	0	1
										KIF26A	1	0	1
										KIF4A	1	0	1
										KLF4	1	0	1
										KLRD1	18	1	17
										LGMN	1	0	1
										LIPG	1	0	1
										LPAR1	7	0	7
										MCM10	3	0	3
										MCOLN2	29	7	22
										MCOLN3	29	7	22
										MFNG	2	1	1
										MIF	2	2	0
										MME	1	0	1
										MMP10	2	0	2
										MMP11	1	0	1
										MMP3	13	0	13
										MND1	1	0	1

										MS4A2	6	0	6
										MTMR6	8	1	7
										MYBL2	4	0	4
										MYH2	2	0	2
										NCALD	8	7	1
										NEDD4L	2	0	2
										NEK2	1	1	0
										NEK7	3	0	3
										NET1	4	0	4
										NEU1	3	0	3
										NFYB	1	0	1
										NIT2	4	0	4
										NQO1	1	0	1
										NR2F2	1	0	1
										NR3C1	14	2	12
										NRG3	18	2	16
										NUP107	7	5	2
										NUP155	7	4	3
										OPTN	1	1	0
										ORC6	3	2	1
										P2RX1	29	7	22
										PAFAH1B3	5	1	4
										PAPSS1	11	5	6
										PARVB	1	0	1
										PDCD1LG2	2	0	2

										PDE1C	3	9	-6
										PDE2A	4	9	-5
										PDE3B	4	9	-5
										PDE4D	3	9	-6
										PDGFB	29	1	28
										PDZD11	8	6	2
										PF4	7	0	7
										PLA2G7	5	1	4
										PLCB4	7	1	6
										PLCH1	6	1	5
										PLK4	1	0	1
										PLOD2	21	0	21
										POLR2H	3	3	0
										PPARGC1A	4	0	4
										PPP2CB	3	5	-2
										PRKCE	4	0	4
										PRMT5	2	1	1
										PSENEN	7	0	7
										PSMB3	12	0	12
										PTPRC	4	0	4
										RAB11A	4	0	4
										RACGAP1	5	3	2
										RBBP8	1	0	1
										RELN	1	0	1
										RFC4	6	0	6

										RGS18	3	0	3
										RGS2	1	0	1
										RPS6KA2	1	0	1
										RYR2	29	0	29
										SACM1L	5	0	5
										SEC24A	4	0	4
										SEMA3E	2	0	2
										SFN	1	1	0
										SGPP2	2	0	2
										SH3BP5	3	0	3
										SH3GL2	2	0	2
										SHANK3	1	0	1
										SLA	3	0	3
										SLC10A2	8	6	2
										SLC12A7	7	6	1
										SLC1A1	10	8	2
										SLC22A4	8	6	2
										SLC26A2	3	0	3
										SLC28A3	8	6	2
										SLC35B2	4	3	1
										SLC38A2	8	6	2
										SLC38A5	8	6	2
										SLC5A3	8	6	2
										SLC5A6	8	6	2
										SLC7A11	1	0	1

										SLC9A3R1	9	1	8
										SLC9A3R2	9	1	8
										SPTBN2	2	0	2
										ST6GALNAC2	6	0	6
										ST6GALNAC3	7	1	6
										ST6GALNAC5	7	1	6
										STARD13	4	0	4
										STARD8	4	0	4
										TBC1D4	1	0	1
										TEK	5	0	5
										TNFAIP3	1	0	1
										TPSAB1	1	0	1
										TREM2	18	1	17
										TRIP10	5	0	5
										TUBG1	1	1	0
										TYROBP	18	2	16
										VEGFC	1	0	1
										VLDLR	3	0	3
